# Work participation in adults with rare genetic diseases - a scoping review

**DOI:** 10.1186/s12889-023-15654-3

**Published:** 2023-05-19

**Authors:** Gry Velvin, Brede Dammann, Trond Haagensen, Heidi Johansen, Hilde Strømme, Amy Østertun Geirdal, Trine Bathen

**Affiliations:** 1 TRS National Resource Centre for Rare Disorders, Sunnaas Rehabilitation Hospital, Nesoddtangen, Oslo, 1450 Norway; 2 Library of Medicine and Science, University of Oslo, Oslo, Norway; 3 Department of Social Work, Child Welfare and Social Policy, Faculty of Social Science, Oslo Metropolitan University, Oslo, Norway

**Keywords:** Scoping review, Rare diseases, Work participation, Work disability, Employment

## Abstract

**Background:**

Work participation is a crucial aspect of health outcome and an important part of life for most people with rare genetic diseases. Despite that work participation is a social determinant of health and seems necessary for understanding health behaviours and quality of life, it is an under-researched and under-recognized aspect in many rare diseases. The objectives of this study was to map and describe existing research on work participation, identify research gaps, and point to research agendas in a selection of rare genetic diseases.

**Methods:**

A scoping review was performed by searching relevant literature in bibliographic databases and other sources. Studies addressing work participation in people with rare genetic diseases published in peer reviewed journals were assessed using EndNote and Rayyan. Data were mapped and extracted based on the research questions concerning the characteristics of the research.

**Results:**

Of 19,867 search results, 571 articles were read in full text, and 141 satisfied the eligibility criteria covering 33 different rare genetic diseases; 7 were reviews and 134 primary research articles. In 21% of the articles the primary aim was to investigate work participation. The extent of studies varied between the different diseases. Two diseases had more than 20 articles, but most had only one or two articles. Cross-sectional quantitative studies were predominant, with few utilizing prospective or qualitative design. Nearly all articles (96%) reported information about work participation rate, and 45% also included information about factors associated with work participation and work disability. Due to differences in methodologies, cultures and respondents, comparison between and within diseases are difficult. Nevertheless, studies indicated that many people with different rare genetic diseases experience challenges related to work, closely associated to the symptoms of the disease.

**Conclusion:**

While studies indicate high prevalence of work disability in many patients with rare diseases, the research is scarce and fragmented. More research is warranted. Information about the unique challenges of living with different rare diseases is crucial for health and welfare systems to better facilitate work participation. In addition, the changing nature of work in the digital age, may also open up new possibilities for people with rare genetic diseases and should be explored.

**Supplementary Information:**

The online version contains supplementary material available at 10.1186/s12889-023-15654-3.

## Background

Work participation (WP) has been found to be beneficial for health status, as it improves functional outcomes, social integration, satisfaction with life and financial status [1, 2]. However, WP seems to be an under-recognized and under-researched aspect in many rare diseases (RDs), even though “the ability to work” is identified as an important research area for people worldwide with RDs [3, 4]. The United Nations (UN) recognizes that persons living with RDs are often disproportionately affected by poverty, discrimination and work-related challenges. Therefore, there is a particular need to address challenges in access to, retention of, and return to work for people living with RDs [3].

In Europe a disease is deemed to be rare when it affects no more than 1 in 2,000 persons [5–7], and in the USA when it affects fewer than 200,000 people at any given time [5, 7]. There are approximately 7,000 distinct RDs, affecting 18 to 30 million Europeans and 263 to 446 million people worldwide [5, 7]. An estimated 72% of RDs have a genetic origin [7] and 70% with childhood onset [7]. Approximately 95% of RDs currently have no approved treatment [5, 7] and RDs create significant challenges for affected individuals and society as a whole. The impacts are often unexplored and range from psychological and physical symptoms, seriously compromising participation in work and daily life (3,8.9,10). The combination of the severity of illness, diagnostic uncertainty, and lack of effective treatments also has a strong impact on persons with RDs [8–12]. Despite the heterogeneity of RDs, affected individuals seem to face many similar problems related to the rarity of the disease [3, 8, 10, 11], such as lack of information and competence [7–9], stigma, and being misunderstood and rejected by the health and welfare system [8, 12–14]. The United Nations acknowledge that people living with a RD may be psychologically, socially and economically vulnerable throughout their life course, facing specific challenges in several areas including, education, employment and leisure [3]. The French barometer survey [4] of RDs found that 50.7% did not work or had stopped working due to the disease. The consequences for both patients and families were income reduction, which added a hurdle to the daily life difficulties [4]. Studies also indicate that having a RD can impact work life balance, absence from work, hamper professional activity, and increase the economic burden [4, 8, 9, 15–17]. Being employed and working is generally the most important means for obtaining adequate economic resources, which are essential for material well-being and participation in society for people with RDs [1, 2, 8].

Studies have also investigated the socioeconomic costs of RDs, quantifying the economic burden of RDs, including the productivity loss due to work [9, 18–22]. It is estimated that the average productivity loss (work disability, absenteeism and decreased work productivity) for each person with RDs varied from 3,000 to over 30,000 euro each year [9, 18, 20–22]. Lack of WP seems to affect both the economic growth and the social inclusion levels in society, and has several consequences on the individual level for people with RDs [21, 23]. Work disability is linked to higher prevalence of depression and anxiety, lower quality of life, low income and dependency of social security income [1, 2, 23].

### The scientific rationale for this study in the context of the state of art

Despite that the right to work and being employed is a fundamental right enshrined in Article 27 of the UN [24], only 50% of individuals with disabilities are employed compared to 74.8% of persons without disabilities in European Union (EU) [25]. The research on WP in people with RDs is limited although it is recognized that persons with RDs have unique challenges related to the rarity of the disease, included work-related challenges [3, 4, 8, 13]. Considering the multifaceted nature of the challenges faced by individuals with RDs, more knowledge about the particular challenges and needs related to WP is important to promote wellbeing and full, equal, and meaningful participation in society for these patient groups [3]. A better understanding of the existing research on WP in RDs and effort to improve the inclusion of people with RDs in the workforce seems necessary.

To our knowledge an overview of the characteristics of the literature of WP in adults with RDs is lacking. A scoping review could serve as a precursor for systematic reviews with specific research questions within one or several diseases and of the elucidated themes. The findings could report on the range of evidence available and the types of evidence that address and inform practice in this field. A baseline for further studies is to have overview of how studies define and describe work-related aspects, the amount of primary research studies versus secondary studies (systematic reviews) and investigate if work-related aspects are primary outcome or not. Furthermore, an overview of the characteristics of investigated patient populations, different research questions and the methods used to investigate WP in RDs may be of importance. Therefore, the aims of this scoping review were:

1. To systematically identify, map, and describe the characteristics of pertinent research and present work participation outcomes of adults with genetic RDs published between 2000 and 2021.
2. To identify research gaps and point to research agendas concerning work participation in RDs.

## Methods

### Study design

This scoping review was conducted according to the Joanna Briggs Institute and Collaborating Centres’ guidance for conducting scoping reviews [26] and aligned with the PRISMA-ScR guidelines [27] (supplementary appendix 1), on peer-reviewed papers from 2000 and onward.

As the parameters for scoping review do not typically call for critique of the methodological quality of included studies or meta-analyses [28, 29], we only examined the extent, range and nature of research on WP in adults with RDs: determined the value and potential for undertaking full systematic reviews, summarized research findings, and identified research gaps in the existing literature [29, 30]. We extracted and presented some results from included articles but did not attempt to assess certainty or synthesize the results similar to what is done in systematic reviews [27, 31, 32].

We followed the iterative six stages process of Arksey and O`Malley [30] for scoping review: (i) identified research questions, (ii) identified relevant studies, (iii) selected pertinent studies, (iv) charting data, (v) summarized and reported the results, and (vi) consulted stakeholders and experts for informing and validating the study findings.

The study protocol is available on request.

### Stage I: research questions

Our review was guided by the question “What are the characteristics of research on work participation and work disability in people with RDs?”. Seven specific research questions were developed via relevant literature and research meetings:

1. What is the extent of secondary research articles (i.e., systematic reviews), and primary research articles on WP in people with different genetic RDs?
2. Where and when have the studies been conducted and published (i.e., country of participants, publication years)?
3. How much focus is given to work participation and to which extent is WP the main focus of the research?
4. What type of population groups are studied (i.e., diagnoses, sample sizes)?
5. What type of study design and assessment methods have been used (study specific, standardized work-related questionnaires, or qualitative methods)?
6. What type of research questions are being addressed (i.e., prevalence, associations, treatment effects, development or validation of assessment methods, experiences and perceptions or other aspects)?
7. What are the main results reported in the included studies?

### Stage II: identifying relevant studies

#### Eligibility criteria

Our eligibility criteria were based on a preliminary review of a subset of relevant literature on WP in people with disability and people with RDs. Due to the vast number of rare diseases, estimated to be around 7,000, it was not feasible to conduct comprehensive searches for all of them while ensuring efficient management of search results. Consequently, we made the decision to restrict our search to articles on rare genetic diseases only.

The framework for the search strategy (additional appendices 2,3) was developed in consultation with the medical librarian, underpinned by the key inclusion and exclusion criteria (see Table 1). These criteria were categorized according to the broad Population-Concept-Context (PCC) [33]: (i) Population: Studies of adults affected with RDs according to the Orphanet classification, including orphan-codes for each disease [34, 35]. (ii) Concept: Studies with at least one aim to describe WP and predictor variables or factors associated with WP. A work-related study was defined as any study addressing work-related issues. (iii) Context: All relevant articles written in English, German, French, Norwegian, Swedish, and Danish languages that had an English abstract were included. An English abstract was necessary so that the articles would be captured by our search terms.

**Table 1:** Inclusion and exclusion criteria

Inclusion criteria	Exclusion criteria
**Population** - Adults (≥ 18 y) with a rare genetic disease according to the Orphanet classification - Studies including a broader population were included if i) presenting separate data on at least 6 or more persons with a rare genetic disease Ii) the mixed population included ≥ 80% of the study population with a rare genetic disease. **Concept - Topic of interest** - Studies presenting at least one aim of investigating prevalence, associations, intervention/treatment, experiences and other aspects of work participation, employment, work disability, vocational situation, measured with any kind of questions/questionnaire **Context** - Studies from all countries included - Papers written in English, German, French and Nordic language, including an English abstract. **Type of publications** - Peer reviewed articles - Original research, primary studies - Secondary research studies: reviews - All types of study designs published between 2000 and onwards.	**Population** - People with rare genetic disease and cognitive affection, other non-genetic rare diseases, studies including less than 80% adults. - Family members/caregivers/professionals to people with rare diseases or paediatric patients with RDs - Studies with broader populations (i) presenting data on less than 6 cases (ii) or not separate results of ≥ 80% with genetic rare diseases of the study sample. **Concept – Topic of interest** - Studies including no information about work participation, employment, work disability, vocational situation. **Context** - No limitations - Any other language **Type of publications** - Conference abstracts, commentaries, essays, consensus statements, book chapter reports, economic analyses, articles dealing with legal or ethical issues, unpublished data (grey literature), study protocols or guidelines. - Papers published before 2000, due to the changes in work-related politics, work condition an attitudes to disability

Only peer-reviewed papers published from 2000 onwards were included due to changes in work-related policies, work conditions and attitudes towards disabilities before the millennium.

#### Search strategy

Systematic searches were performed in the bibliographic databases MEDLINE (OVID), CINAHL (EBSCO), APA PsychoInfo (OVID), AMED (OVID), Embase (OVID), ERIC (OVID), Cochrane Database of Systematic Reviews (Wiley), Cochrane Register of controlled Trials (Wiley), SveMed+, Scopus (Elsevier), and the following Web of Science databases: Science Citation Index Expanded, Social Sciences Citation Index, Arts & Humanities Citation Index, Conference Proceedings Citation Index-Science, Conference Proceedings Citation Index Social Science & Humanities, Emerging. The searches were run on 27th September 2021, by an academic librarian (HS). The search consisted of a combination of subject headings (where applicable) and text words for RDs and work. Complete search strategies are available in supplementary appendix 2 and 3. The search results were exported to EndNote software and duplicates were removed [36]. In addition, we conducted a grey literature search and hand-searched the reference lists of the included studies. Experts in the field were also asked for additional publications.

### Stage III: selection of publications

The Rayyan software [37] was used to screen the records, and the authors were blinded for each other’s decisions. A pilot screen was conducted of approximately 5% of the articles to ensure that all researchers understand the inclusion and exclusion criteria. At least two authors (GV/BD, GV/TH, GV/HJ) independently assessed the titles and abstracts of the identified records to evaluate eligibility against the selection criteria. Four authors (GV, BD, TH, HJ) assessed the articles in “conflict” after conducting the Rayyan blinding. Potentially relevant publications were retrieved and read in full text, assessed by two authors (GV/BD, GV/TH, GV/HJ) independently. Disagreement was resolved by discussions and involving a third author (TB or AØG), using the inclusion and exclusion criteria.

### Stage IV: charting data

Two authors (GV/BD, GV/TH, GV/HJ) independently charted and extracted study data into a priori data extraction form in a spreadsheet and the other authors (TB or AØG) checked and verified the accuracy. The following data were extracted from each study: Bibliographic data, nationality/country of participants, study aim, participants’ data (number, gender, age, diagnosis, and recruitment location), study design, methodology, and outcome measures for WP and which research questions on WP the study had investigated. In addition the WP-rate (prevalence of people working), and/or associations (variables associated to WP), and other aspects (patients’ views and experiences, intervention effects, development/validating outcome measures on WP), and how much focus the study had on WP (primary- or secondary aim/outcome). From papers that included other populations or themes in addition to WP in RDs, we only selected and presented data on WP in the RDs.

### Stage V: summarizing and presenting results

All publications were sorted according to diagnostic groups and specific diagnosis using EndNote [36]. Extracted data according to the prior form were presented descriptively in a spreadsheet for each diagnostic group and disease in the supplementary file 4. Descriptive statistics, including frequencies and mean value were presented in both text and figures using Microsoft Excel [38].

### Stage VI: consulting stakeholders for informing and validating study finding

The study results have been reviewed, discussed and validated with stakeholders and experts in the area of people with genetic RDs, and presented as digital poster and oral presentation at EURODIS conference in June 2022 included a discussion of the main results [39].

## Results

**Fig. 1:**
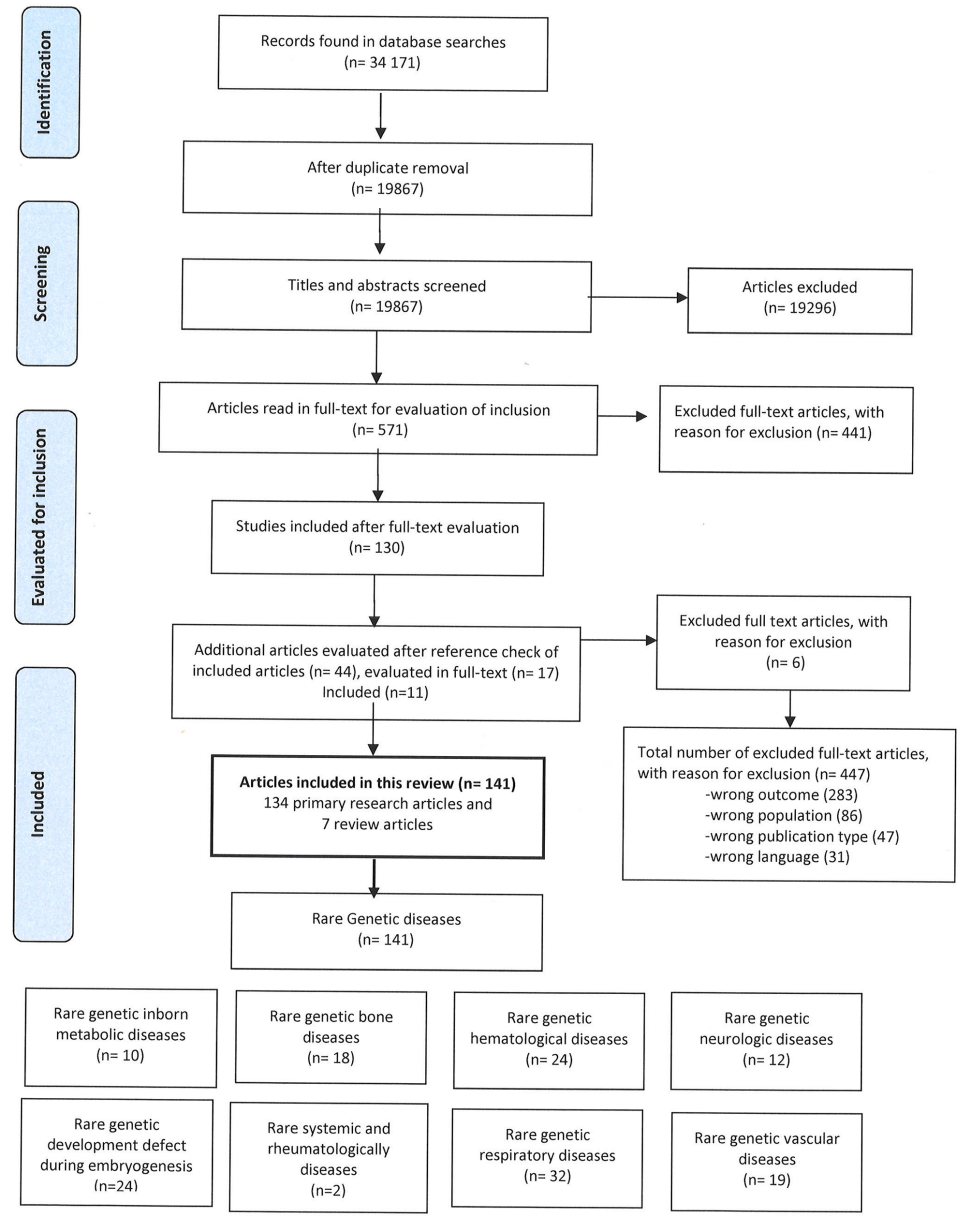
Flow chart of the search and selection process

The searches resulted in a total of 34,171 hits, reduced to 19,867 records after deduplication [40]. After screening the titles and abstracts, the blinding of Rayyan showed that 253 (1.3%) papers were in “conflict” and 427 included. After assessing the articles in “conflict”, 144 were included to be read in full text, the others were excluded. Thus, 571 articles were read in full text and 19,296 were excluded. After assessing the full text articles 130 (22.8%) were included. After reference check of included articles and grey literature searches, additionally, 11 articles were included, giving a total of 141 included articles: 7 secondary research articles (reviews) and 134 primary research articles (supplementary appendix 3 and 4). Figure 1 shows a flow chart of the screening and inclusion process with the distribution of included references according to the Orhpanet classification of different diagnostic groups [34, 35].

The 141 identified articles covering 33 different genetic RDs (Table 2). Key findings of each of the included articles identified in the literature search is given in Table 2, more detailed information is given in supplementary appendix 4 (included articles).

**Table 2:** Diagnostic groups and diseases reported in included articles

Rare genetic diseases	Number of articles	Number of respondents
*Rare genetic inborn metabolism disease*		
Fabry disease	1	184
Gaucher disease type 1	1	192
Glycogen storage disease type 1	1	34
Pompe disease	3	405
Porphyria	2	473
Familial chylomicronemia syndrome	2	203
*Rare genetic bone diseases*:		
Multiple osteochondromas	2	205
Osteogenesis imperfecta	4	180
X-linked hypophosphatemia	4 (1 review)	57
Primary bone dysplasia/short stature*	3	314
Achondroplasia*	3	257
Diastrophic dysplasia, (Diastrophic dwarfism) *	1	68
Fibrous dysplasia	1	56
*Rare genetic haematological disease*:		
Haemophilia	22 (2 reviews)	5588
Congenital factor VII deficiency	1	25
Chronic coagulation disorder	1	30
*Rare genetic neurologic diseases*		
Charcot-Marie-Tooth disease (hereditary motor and sensory neuropathy type 1)	4	332
Duchenne muscular dystrophy	1	65
Facioscapulohumeral muscular dystrophy	1	25
Limb-girdle muscle dystrophy	1	14
Muscular dystrophies (mixed population)	1	44
Myotonic dystrophy	4	674
*Rare genetic developmental defect during embryogenesis*		
Neurofibromatosis	9	1205
Spinal muscular atrophy type 2	4	303
Turner syndrome	9	1237
X-linked Emery-Dreifuss muscular dystrophy	1	24
22q11.2 deletion syndrome	1	144
*Rare systemic and rheumatologic diseases*		
Hereditary angioedema	2	259
*Rare genetic respiratory diseases*		
Cystic Fibrosis (2 review)	32 (2 reviews)	16661
*Rare surgical thoracic diseases*		
Marfan syndrome (2 review)	14 (2 reviews)	2448
Loeys-Dietz syndrome and vascular Ehlers-Danlos syndrome (mixed populations)	2	104
Rare disease with thoracic aortic aneurysm and aortic dissection (mixed populations)	3	439

### Characteristics of the secondary research articles (i.e., review articles)

Seven reviews [41–47] were identified, but in only one [43] the major outcome was to investigate WP. This was a systematic review about the impact of cystic fibrosis (CF) on work life, including 15 articles, all addressing WP. The review showed that a significant proportion of CF patients retained a paid job, both full- and part time schedules, with a global worldwide employment rate ranging from 44 to 86%. This systematic review emphasized the importance of interdisciplinary teams to carefully assess work function as part of the routine clinical management [43]. In the other reviews, WP was investigated as secondary outcome. One systematic review [46] of “quality of life in people with cystic fibrosis” included only two articles about work related aspects, nevertheless indicated that the disease had adverse impact on work absenteeism and productivity. Two reviews dealt with haemophilia disease. One systematic review [42] on “psychosocial aspects of haemophilia”, included three studies on work related aspects. The other was a snapshot review [47] of “the social burden of haemophilia”, which included eight studies dealing with work-related aspects. Both reviews indicated that people with haemophilia are less involved in full-time paid work, and many experience occupational disability, including productivity loss due to the disease. Two reviews dealt with psychosocial aspects of Marfan syndrome, one [44] included five articles and the other [45] eight articles on WP. Both indicated that Marfan syndrome impacts the ability to work, and that many people retire earlier compared to the general population. Workplace discrimination was also reported, and decreased WP was associated with depression and low self-esteem. The last review was a systematic review [41] of the “burden of having X-linked hypophosphatemia”, included three articles on work-related aspects. This review indicated that the disease impacts the patients’ possibility to work and many retire early. Work disabilities were associated with denial and psychosocial problems. More details of the reviews are presented in supplementary appendix 4 (included articles).

Table 3 shows an overview of the review articles.

**Table 3:** Included review articles

Disease	Review design	Total number of included articles	Number of articles on WP	Outcome level
Cystic fibrosis (43)	Systematic review –Quality Rating according to NOS*	15	15	WP was major outcome
Cystic firbosis (46)	Systematic review – quality assessmet of included articles	23	2	WP secondary outcome
Haemophilia (42)	Systematic review -no quality assessment of included articles	25	3	WP secondary outcome
Haemophilia (47)	Snap shot review	Not described	8	WP secondary outcome
Marfan syndrome (44)	Systematic review - quality assessment of included articles	20	5	WP secondary outcome
Marfan syndrome (45)	Literature review	40	8	WP secondary outcome
X-linked Hypophosphatemia (41)	Systematic review-Quality rating according to NOS	90	3	WP secondary outcome

*NOS- Newcastle Ottawa Scale

### Primary research articles

We identified 134 primary research articles presenting data on work-related aspects on 33 genetic RDs. Except from one publication in German [48], all articles were in English language. The most frequently studied diseases were cystic fibrosis with 32 (24%) articles, haemophilia with 24 (18%) and Marfan syndrome with 14 (10%) articles. These three diseases accounted for 52% of all articles included.

Eighteen (55%) of the 33 diseases had only one or two articles addressing WP.

#### Context and level of outcome

**Fig. 2:**
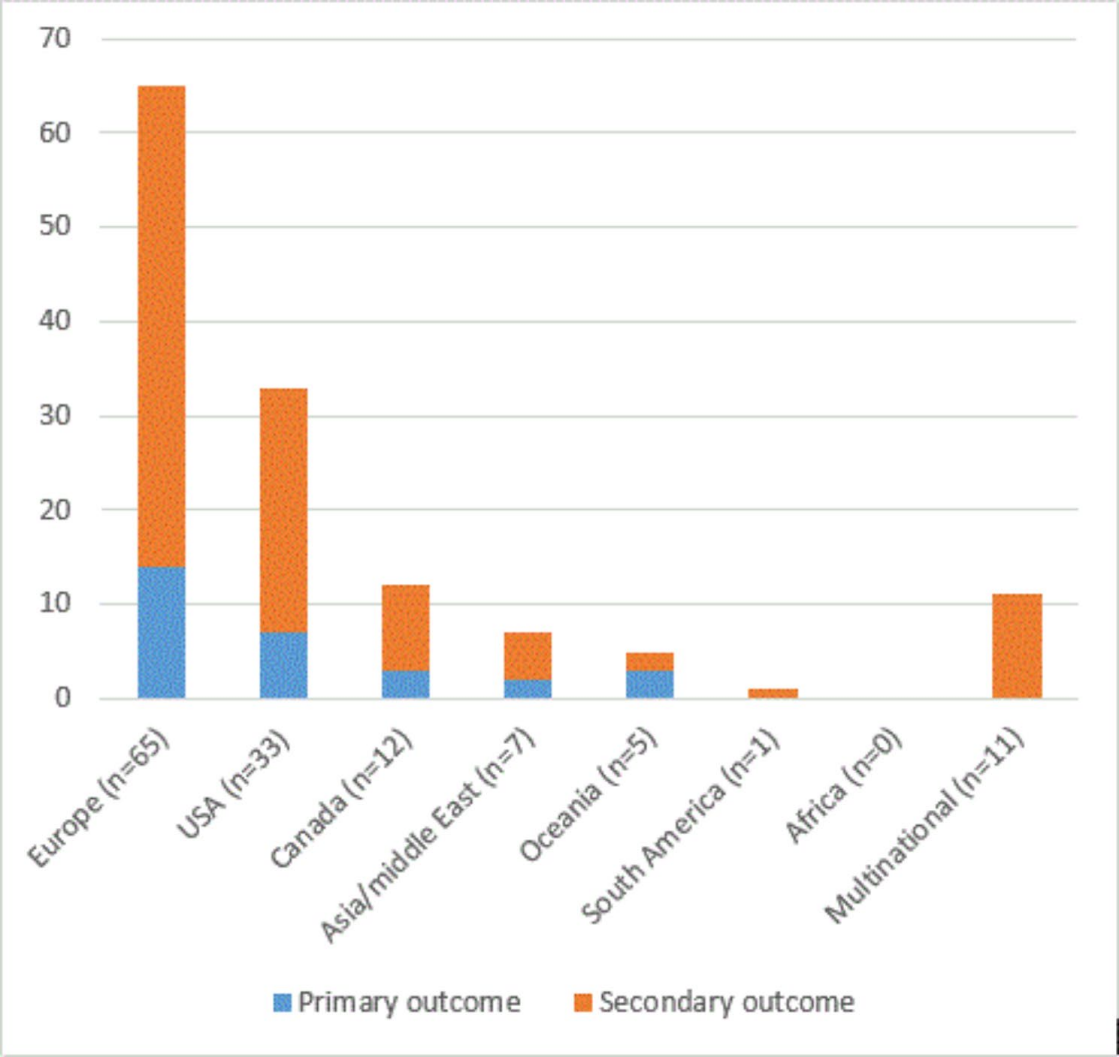
Context and WP outcome level of included articles

Only 11(8%) were international cooperation studies [49–59], the rest were based in a single country and reported national data from Europe (n = 65/48%), USA (n = 33/25%), Canada (n = 12/9%), Asia (n = 7/5%), Oceania (n = 5/4%) and South America (n = 1/1%), representing a total of 26 different countries. No studies from the African continent were identified. In 29(21%) of the primary articles [57, 60–87] the main aim/outcome were to investigate WP, most were from European countries. Figure 2 shows the geographic context and level of outcome on WP.

**Fig. 3:**
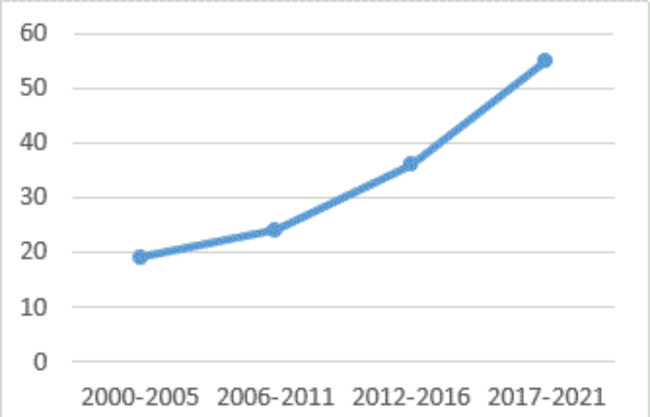
Published articles in from 2000 to 27th September 2021

Of the 29 articles with WP as primary aim/outcome, 18 (62%) articles dealt with cystic fibrosis [68–84, 86], 3 (10%) with haemophilia [60–62], 2 (7%) with Turner syndrome [65, 66] and 2 (7%) with neurofibromatosis [64, 87], and 4 (14%) different diseases [57, 63, 67, 85] had one article with primary outcome on WP. For further information see supplementary appendix 3 (included articles). Most (n = 91/68%) articles were published the last decade. Figure 3 shows the total number of primary articles published in period from 2000 to 2021.

**Fig. 4:**
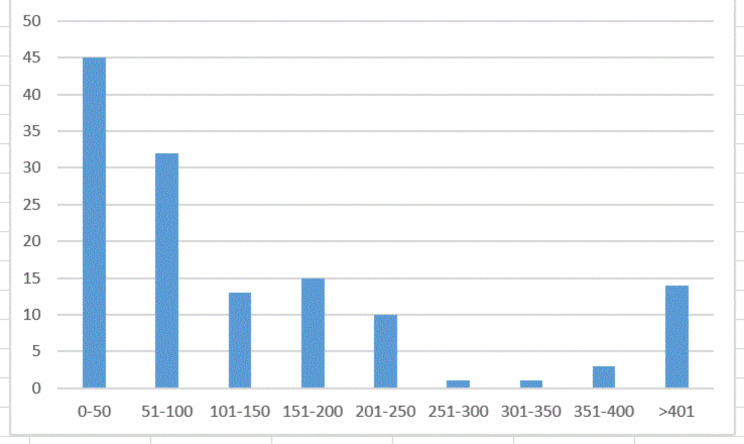
Sample size of the included articles

Most of the primary research articles had small sample sizes, 45 (34%) had 50 or less respondents and 77 (57%) had 100 or less (Fig. 4). Twelve (9%) articles had more than 400 respondents; dealing with cystic fibrosis [76, 83, 88–90], haemophilia [53, 55, 60, 91, 92], neuro-fibromatosis type 1 [93] and Marfan syndrome [59]. Three of these articles [83, 89, 90] included more than 2,000 respondents and all dealt with cystic fibrosis. The overall mean of respondents in all the included studies was 217. Figure 4 show the number of studies with different sample sizes.

The total proportion of all respondents in the primary studies was approximately 32,249, with a variation from 9 [94, 95] to 7,427 [90]. Five diseases (cystic fibrosis, haemophilia, Marfan syndrome, neurofibromatosis and Turner syndrome) accounted for 84% (n = 27,139) of the total proportion, and 51% (n = 16,661) had cystic fibrosis. The study samples were mainly recruited from hospitals, most commonly from dedicated disease clinics (59%) and general hospitals (22%), or patient associations (11%), registry data (6%), or open web and advertisement in newspaper (2%).

The mean age of the respondents was approximately 37 years, with a slightly greater percentage of males (55%). One disease (Turner syndrome) included only females and another disease (haemophilia) mostly male.

#### Study design and methods

**Fig. 5:**
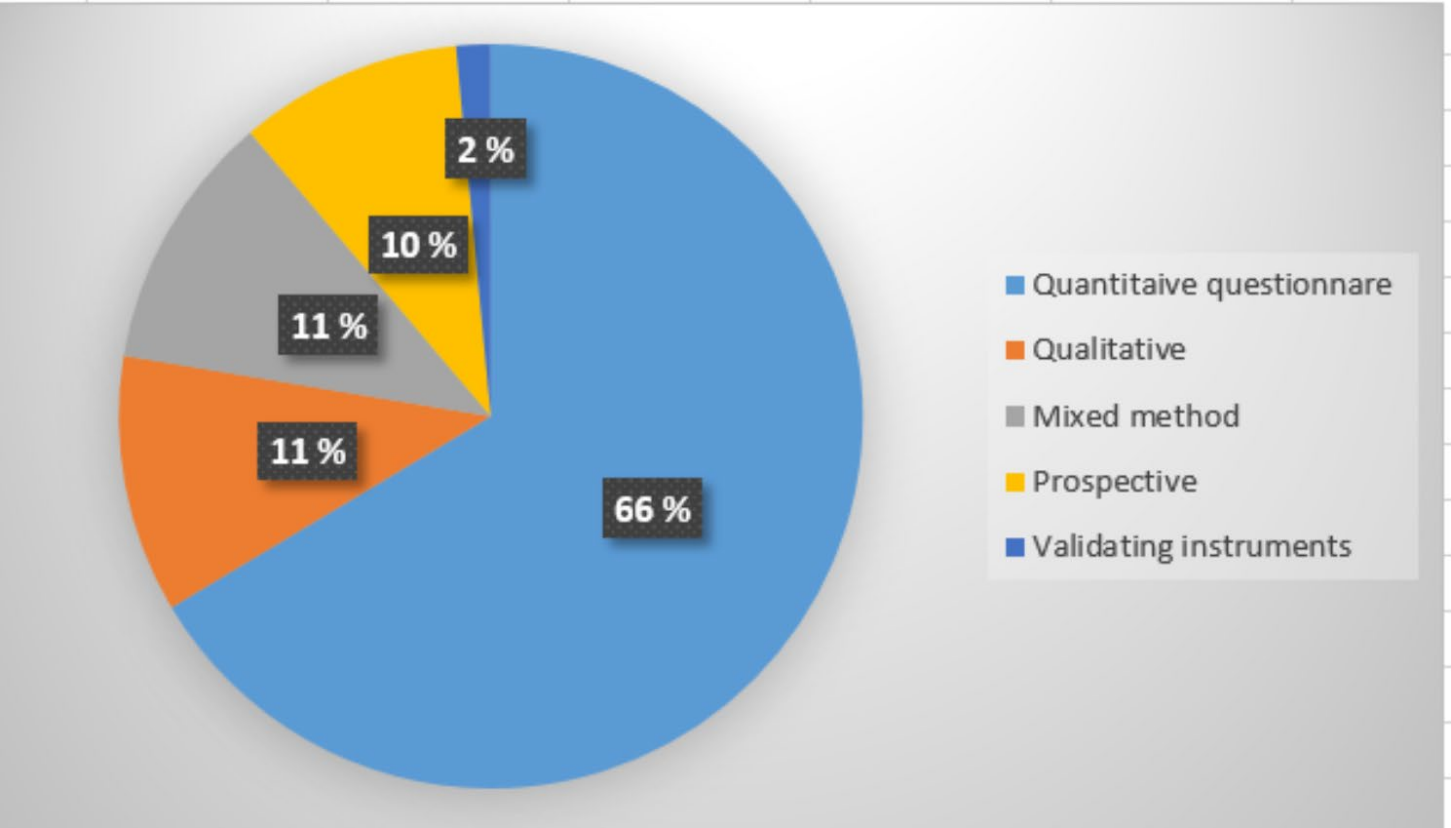
Study design of the included articles

There were a wide variation of design and approaches in the different studies dealing with WP in RDs. The most common methodology was cross-sectional quantitative study (n = 89/66%), using study specific questionnaire, administrated face-to-face, on internet or postal. Less common was prospective studies (n = 13/10%) [60, 63, 70, 71, 76, 80, 83, 96–101] or qualitative studies (n = 15/11%) including either individual interviews [51, 57, 82, 95, 102–110] or focus group interviews [52, 111]. Fifteen (11%) used mixed methods, combing quantitative questionnaire with semi-structured individual interviews [62, 86, 94, 116–123], with focus group [124], or open-end questions [56]. Two (2%) studies [124, 123] were validating an instrument, one [124] on instrument on distress and one [123] on health literacy. No randomized controlled trials (RCT) or intervention studies on WP were identified. Figure 5 shows the study design of the included articles. The methodologies of the 134 primary studies are illustrated in Fig. 5.

Across the quantitative questionnaire studies [48–50, 53–55, 58, 59, 61, 64–69, 72–75, 77–79, 81, 84, 85, 87–93, 125–181]^,^, a wide range of different issues were investigated mostly using study specific questions for measuring WP. Only 12 (9%) studies [54, 58, 66, 68, 78, 79, 84, 87, 99, 124, 159, 164] used validated instruments, based on eight different work-related instruments. The Work Productivity and Activity Impairment Questionnaire (WPAI) was the most frequently used instrument. No studies used diseases specific instruments, all were generic. Table 4 shows an overview of the most frequently used instruments for measuring WP.

**Table 4:** The instruments used for measuring work participation

WP instruments	Number of studies (references)
The Work Productivity and Activity Impairment Questionnaire (WPAI)	6 studies (54,58,78,112,159,164)
General Nordic Questionnaire for Psychological and Social factors of Work (QPS),	2 studies (66,99)
The employment Hope Survey-Short (EHS-14)	1 study (87)
The Barrier to Employment Success Inventory (BESI).	1 study (87)
The World Health Organization Health and Work Performance Questionnaire (HPQ)	1 study (79)
The Stanford Presentism Scale SPS-6:,	1 study (84)
The Work Ability Index (WAI)	1 study (78)
The Standard Vocation Preparation (SVP)	1 study (68)

Some studies used items of questions on WP from a national labour force survey [64, 65, 85, 97, 178–180], or validated instruments on other aspects than WP, including some questions about WP [49, 71, 74, 75, 79, 151, 152, 169]. More than half (n = 79/59%) of the included studies did not described questions used for exploring WP.

#### Description of the results from the included studies

Most articles (n = 129/96%) reported data on prevalence of WP, such as work participation rate (full/part time) and/or work disability rate (disability/rehabilitation pension) across all included diagnostic groups. The mean work participation rate of the total study samples in the primary articles was calculated to approximately 55.1%, with a variation from 0% [152] to 100% [101].

Nearly half of the articles (n = 60/45%) reported associations to WP. WP was reported negatively associated to the severity of the disease, fatigue, pain, depression, decreased quality of life, lower education level and higher age [50, 55, 57, 60, 61, 63, 64, 66, 67, 69, 70, 73, 81, 83, 85, 87, 89–92, 96–99, 124, 124, 125, 128–130, 134, 135, 139, 140, 143, 146–149, 151, 153, 154, 156, 159–161, 164, 165, 167–170, 172, 174, 176–181].

Less than half (n = 53/40%) of the primary studies also reported other aspects related to WP, such as patients‘ experiences and perceptions, how the disease impact work [49–54, 56, 57, 60, 86, 94, 95, 99, 102, 103, 105–111, 124, 123, 124, 124, 126, 127, 131, 132, 134, 135, 141, 150, 162, 174], average age for leaving work [85, 97, 176], work place experiences [46, 72, 82, 104, 149], stigma/discrimination [84, 124], experienced meaning of work [70, 75, 80, 124, 123, 123] and productivity loss [84, 96] (see supplementary appendix 4, the included articles).

## Discussion

This scoping review included 141 articles addressing WP in 33 genetic RDs. This may seem like a large number, but only 21% investigated WP as primary outcome. Most studies were based on small sample sizes with various research design and methodologies. Quantitative cross-sectional questionnaire studies were predominant, with few utilizing qualitative, prospective or mixed method design. The extent of the studies varied for each disease and the vast majority were conducted in the Western countries. While the results indicate that many people with RDs experience WP barriers as a results of their condition, caution is needed due to the variation within and between diagnoses, and the differences in the use of methodologies and instruments.

### Secondary versus primary studies

Seven reviews were identified covering WP in RDs, with only one review focusing mainly on WP. This indicates a research gap of the summary and critical evaluation on existing research in this area. Systematic reviews are crucial for determining existing knowledge gaps and future research [28]. Additional, they provide vital guidance for policymakers and healthcare providers in developing clinical guidelines and directing clinical practice [28]. Some of the disease (e.g. cystic fibrosis, haemophilia, Marfan syndrome) had several studies on WP, suggesting the feasibility of systematic reviews. These reviews could provide a more comprehensive understanding of critical issues related to WP, such as prevalence and factors that promote inhibits work possibilities across different rare diseases. Such reviews may also be helpful to provide as guidance to formal job counselling or career choices for people with RDs.

### Characteristics of the primary studies

#### Sample sizes and diseases

Our results confirm that most studies on WP in RDs have small sample size. The challenges related to small sample sizes in RDs have been emphasized in several studies [182, 183]. It may poses recruitment challenges, lack of sufficient statistical power, and questions regarding the representativeness of the available data for the population [182, 183]. Surprisingly, 12 of the included studies in our review had more than 400 participants. Three of these included more than 2,000 respondents each and were conducted in United Kingdom and the USA. All three dealt with cystic fibrosis, one of the most common life-shortening genetic RDs, affecting more than 10,000 people in the United Kingdom and 90,000 people worldwide [184]. The larger sample sizes in these studies may be attributed to the ease of recruitment in larger countries with dedicated disease specific centres and large patient organizations.

Our review indicated that five genetic RDs covered approximately 84% of the total proportion of respondents, and the remaining 28 diseases only 16%, indicating that the scope of research varies between the genetic RDs. This may reflect the true differences in occurrence or coincidental interest among professionals. Multinational collaboration particularly on the less common and ultra-rare diseases may be essential to achieve more knowledge about these patient groups.

#### Geographical setting

Concerning the geographical settings, nearly all studies were conducted in Western countries, (Europe, USA, Canada and Australia), few from Asia and South America, and none from Africa. This indicate a gap in research from low-income countries similar to what has been found in other reviews [43, 185, 186] on WP of people with disability. Only a few studies were multinational and none of these investigated WP as a major outcome. Despite that the welfare systems and labour marked are different in various countries, more international collaboration studies using the same study design and measurement methods may contribute to better understanding on how the disease may impact work ability across these cultural differences.

#### Methods and research questions

The vast majority of the studies were cross-sectional quantities questionnaire studies, and only a few studies had qualitative design. Benjamin et al. [187] recommended using a wide range of methods to gain a more comprehensive understanding of patients’ experiences, perceptions and needs. This can provide valuable insight in coping strategies for people with RDs and help identify which aspects of work related issues are important to address in research. The extensive use of cross-sectional methodology currently also limits causal inference in the relationship between disease and the impact on WP. More prospective investigations could assess the possible links between the disease and WP.

Few studies employed validated instruments to measure WP, and the variation in questions and measures utilized makes comparisons between and within diseases challenging. The need for more sensitive and specific outcome measures are emphasized as a challenge in RDs research [183, 187]. To address this, researchers, health professionals, and patient organizations could cooperate to create standardized sets of WP outcomes for a particular disease or groups of RDs. This may enable agreements on what issues that are important to measure, how it should be measured and how the results could be interpreted [187]. WP related questions may be included in patient reported outcome measurements (PROMs) to systematically incorporate patients’ perspectives for measuring outcomes that matters for the patients. Overall, more secondary and primary research, as well as collaboration on instruments and questions, are needed to better understand work-related aspects in RDs.

#### Charting the results of the included articles

Nearly all articles reported WP-rate, and the estimated mean WP-rate (full/part-time) of the respondents in all included studies was approximately 55.1%. This is slightly higher than the employment rate of 49% found in the French barometer survey [4] of adults with different rare diseases. The French barometer survey also found that 50.7% had stopped working due to their disease [4]. Although, the results from our study is comparable with the French barometer survey, caution is needed due to the differences in methodologies, culture and respondents in included articles.

Several studies also reported variables associated to WP, such as disease-related symptoms, the severity of the disease, pain, fatigue and demographic aspects, similar to finding in studies of more common diseases [1, 2, 185, 186]. Identifying both disease-related and others factors influencing WP may be valuable information for better understanding how the diseases and other aspects may influence people`s work capacity [186].

Some studies also reported other work related aspects such as the participants’ perception of how the disease influence WP, discrimination and productivity loss, and nearly all only emphasized challenges related to WP. More studies on coping strategies, successful work integration, useful facilitation measures and adaption in work for people with RDs, could provide valuable information for both health professionals and people with RDs. Our findings suggest that WP studies of people with RDs should account for the multifaceted interplay between biological, personal, environmental and social factors [43, 78, 185, 186]. Better understanding of critical issues related to WP activities, the impact of the disease on several work related outcome, such as career choice, employment status, absence due to sickness, work ability and factors predictive of disability should be addressed in more comprehensive analyses both between and within the RDs.

The United Nations [3] reaffirming that persons living with a RD face challenges in accessing, retaining and returning to work, encourage Member States to promote access to full and productive employment and decent work for persons living with RDs. The need of expanding flexible working arrangement, including the use of information and communication technologies is emphasized as important work-oriented facilitation measures for people with RDs [3]. The ILO Global Business and Disability Network (ILO GBDN) also emphasizes that the digital transformation and the continuous change in the nature of work and skills may be beneficial for people with disability including those with RDs [188]. Increased health literacy and more research on possibilities of reskilling and upskilling people with rare diseases with 21st century skills may be of great importance for in a world of work where physical function paces less importance [188, 189].

## Limitations and strength

A limitations might be that we only included articles about patients with rare genetic diseases, thereby excluded other rare diseases. However, we found this necessary in order to ensure that the inclusion criteria were as clear and transparent as possible. In addition, including approximately 7,000 different rare diseases in this review would have been methodologically challenging. Choice of search words and our cultural and conceptual understanding may have limited our identification of papers and the interpretation of the content of the included studies. The comprehensive searches by an academic librarian in all relevant databases is a strength, nevertheless we might have overlooked some articles. Another strength is the use of Rayyan software with blinded evaluation between the review authors. The process of selecting, charting and extracted data into a priori extraction form may involve some biases, but a strength was that two review authors conducted this independently. Disagreements were solved by discussions in the review team. The classification of different RDs is challenging. We used the Orphanet classification for categorizing of the diagnoses into diagnostic groups, but a limitation may be that many diseases can be categorized into several diagnostic groups and we may have misplaced some diseases. A strength might be that we chose to restrict the focus of our review on WP by only including genetic RDs. This gave us an opportunity to include a wide range of research on WP in different genetic RDs, but also clarify the scope of included rare diseases. The use of specific inclusion criteria and predefined categories is a strength. We also summarized and presented some results from each article in the data extraction table (supplementary appendix Table 4). These results provide insight into work related aspects of different RDs, and provide basic materials for initiating systematic reviews on various diseases. Nevertheless, these results must be treated with caution due to the lack of risk bias assessment of the included articles.

## Conclusion

This scoping review has highlighted that work-related issues are an under-recognized and under-researched topic for most RDs, and that the extent of research varies between the diseases. Studies indicated that many people with RDs experience barriers related to work, closely associated to the severity and symptoms of the disease. The challenge is to develop policies that counter tendencies in the job market to marginalize people with RDs. It is important to gain more insight into the unique challenges faced by people with different RDs to facilitate better vocational situations for these patient groups within the health and welfare system. Therefore, guidelines for research and clinical measurement of work-related aspects should be developed, taking into account the general problems associated with work disability, challenges related to the rarity of the diagnoses, specific medical symptoms of the disease, and the patients’ individual circumstances.

## Electronic supplementary material

Below is the link to the electronic supplementary material.

**Supplementary file 1**: PRISMA-ScR Checklist

**Supplementary file 2**: Main search strategies

**Supplementary file 3**: Search strategies

**Supplementary table 4**: Data extraction of included articles on work participation in rare genetic diseases

## Data Availability

materials. The dataset supporting the conclusion of this article is included within the article (and its supplementary files). An Endnote-file is available on request to the main author.
