# Supplementary material for: Work participation in adults with rare genetic diseases - a scoping review
Source: BMC Public Health. 2023 May 19;23:910. doi: 10.1186/s12889-023-15654-3 (PMC10197424; doi:10.1186/s12889-023-15654-3)
Supplement: Supplementary file 2 — Main search strategies [file 12889_2023_15654_MOESM2_ESM.docx]

**Supplementary file 2: Main search strategies**

Documentation of literature search for «Rare diseases and work participation»

| **Database** | **Number**  **of hits** |
| --- | --- |
| MEDLINE (Ovid) | 3866 |
| Embase (Ovid) | 10243 |
| PsycINFO (Ovid) | 749 |
| AMED (Ovid) | 98 |
| Cochrane Database of Systematic Reviews | 18 |
| Cochrane Central Register of Controlled Trials | 228 |
| CINAHL (EBSCO) | 452 |
| Scopus | 6056 |
| Web of Science | 4071 |
| SveMed+ | 8 |
| Number of reference before duplicate check: | 25789 |
| Number of references after duplicate check: | 13551 |

All searches were performed on 1. April 2020 by Hilde Strømme, adviser/librarian at the Medical Library of University of Oslo.

**Ovid MEDLINE(R) ALL 1946 to March 31, 2020 (Ovid)**

Date for search: 1 April 2020
Number of hits:: 3866

| 1 | Rare Diseases/ or Osteogenesis Imperfecta/ or Exostoses, Multiple Hereditary/ or exp Fibrous Dysplasia of Bone/ or Achondroplasia/ or Marfan Syndrome/ or Ehlers-Danlos Syndrome/ or Loeys-Dietz Syndrome/ or exp Muscular Dystrophies/ or Glycogen Storage Disease Type II/ or exp Porphyrias/ or Hemophilia A/ or Hemophilia B/ or Cystic Fibrosis/ or exp Neural Tube Defects/ or exp Limb Deformities, Congenital/ or Charcot-Marie-Tooth Disease/ or Spastic Paraplegia, Hereditary/ or DiGeorge Syndrome/ or exp Neurofibromatoses/ or Turner Syndrome/ or exp Mitochondrial Diseases/ or Noonan Syndrome/ or Klinefelter Syndrome/ | 221273 |
| --- | --- | --- |
| 2 | ((rare adj3 (disease* or disorder*)) or (orphan adj (disease* or disorder*)) or (osteogenesis adj imperfecta) or brittle bone disease* or fragilitas ossium or osteopsathyrosis or ((lobstein* or bruck*) adj (disease* or syndrome*)) or ((skeletal or fibrous) adj3 (dysplasia* or bone*)) or achondroplas* or Diaphyseal Aclasis or ((Hereditary or Familial or Multiple) adj2 (Exostos* or Chondrodysplas* or Osteochondroma*)) or Bessel-Hagen Disease or (Fibro* adj Dysplasia*) or Jaffe Lichtenstein* or (Marfan* adj2 (syndrome* or disease* or disorder* or abiotrophy)) or (Ehlers adj Danlos) or (Loeys adj Dietz) or (genetic adj3 aortic) or ((musc* or limb-girdle) adj dystroph*) or (Glycogen Storage Disease Type adj (II or "2")) or (glycogenos* adj2 (II or "2" or generali*)) or (Pompe adj (disease* or syndrome* or disorder*)) or porphyria* or (porphyrin adj (disorder* or disease* or syndrome*)) or hemophilia* or haemophilia* or ((cystic or pancreatic) adj2 fibros*) or mucoviscidosis or mucoviscoidosis or (pancrea* adj (fibrocystic or fibros* or cystic)) or myelomeningocele* or (neural tube adj2 defect*) or spina bifida* or (congenital adj3 (limb* or exremit*)) or ((limb* or exremit*) adj3 (deformi* or malform* or anomalit*)) or Arthrogryposis multiplex congenita or Amyoplasia* or (Charcot adj Marie) or (Roussy adj Levy) or Peroneal Muscular Atroph* or Hereditary Areflexic Dystasia* or ("hereditary motor and sensory" adj neuropat*) or (HMSN adj2 (I or II or 1A or 1B or "5" or V)) or HMSN1A or HMSN1B or ((Strumpel* or Struempel*) adj1 (disease or syndrome)) or (familial spastic adj (paralysis or paraparesis or paraplegia)) or (dejerine sottas adj (syndrome or disease)) or (myoton* adj2 (dystroph* or atrophica* or myopathy*)) or (Steinert* adj disease*) or Ricker Syndrome* or PROMM* or (Hereditary adj3 Spastic Paraplegia*) or Spastic Paraplegia Hypertrophic Motor Sensory Neuropathy or "CMT with Pyramidal Features" or (Spastic Paraplegia adj2 ("2" or II)) or SPG2 or (rare adj3 hereditary ataxia*) or ((DiGeorge or Di George) adj (syndrome* or sequence or anomaly)) or ((velocardiofacial or Velo Cardio Facial or 22q11 or vcf or pharyngeal pouch or Thymic Aplasia or Sedlackova or Shprintzen) adj2 syndrome*) or "Autosomal Dominant Opitz G Bbb Syndrome" or "Conotruncal Anomaly Face Syndrome" or Catch22 or neurofibromatos* or recklinghausen* or (multiple adj1 neurofibroma*) or ((Turner* or Ullrich* or XO or 45X) adj3 (syndrome* or disease* or state or status or stigma*)) or (Gonadal Dysgenesis adj XO) or Monosomy X or (Bonnevie Ullrich adj (syndrome* or status)) or (mitochondrial adj (disease* or disorder*)) or respiratory chain deficienc* or oxidative phosphorylation deficienc* or (Noonan* adj3 (syndrome* or disease*)) or ((Klinefelter* or XXY or XXYY or XXXXY) adj3 (syndrome* or trisom* or disease*))).tw,kf. | 269156 |
| 3 | 1 or 2 | 352233 |
| 4 | exp Work/ or Employment/ or exp Rehabilitation, Vocational/ or Workplace/ or exp Occupations/ or Occupational Stress/ or Job Satisfaction/ or Work-Life Balance/ or Sick Leave/ or Absenteeism/ or Retirement/ or Vocational Guidance/ or Career Choice/ or Work Capacity Evaluation/ or Presenteeism/ | 216667 |
| 5 | ((work* adj3 (participat* or disabilit* or abilit* or rehabilit* or challenge* or adapt* or facilitat* or tenure or load or activ* or left or leave or leaving or sustain* or capacit* or measure or measures or protect* or inhibit* or incapacity* or qualit* or life)) or (disabilit* adj3 (pension* or rehabilit* or benefit*)) or ((disabilit* or sick*) adj3 (leave or absent*)) or retirement or retired or retiring or employ* or unemploy* or job or jobs or occupation* or vocation* or career* or workplace* or workforce* or absenteeism or presenteeism).tw,kf. | 932397 |
| 6 | 4 or 5 | 1039989 |
| 7 | 3 and 6 | 5418 |
| 8 | limit 7 to yr="2000 - 2020" | 3866 |

**Embase Classic+Embase <1947 to 2020 March 31> (Ovid)**

Date for search: 1. April 2020
Number of hits: 10243

| 1 | rare disease/ or osteogenesis imperfecta/ or hereditary multiple exostosis/ or exp fibrous dysplasia/ or achondroplasia/ or Marfan syndrome/ or Ehlers Danlos syndrome/ or Loeys Dietz syndrome/ or exp muscular dystrophy/ or glycogen storage disease type 2/ or exp porphyria/ or exp hemophilia/ or cystic fibrosis/ or exp neural tube defect/ or exp limb malformation/ or hereditary motor sensory neuropathy/ or DiGeorge syndrome/ or exp neurofibromatosis/ or exp disorders of mitochondrial functions/ or Noonan syndrome/ or exp Klinefelter syndrome/ | 413849 |
| --- | --- | --- |
| 2 | ((rare adj3 (disease* or disorder*)) or (orphan adj (disease* or disorder*)) or (osteogenesis adj imperfecta) or brittle bone disease* or fragilitas ossium or osteopsathyrosis or ((lobstein* or bruck*) adj (disease* or syndrome*)) or ((skeletal or fibrous) adj3 (dysplasia* or bone*)) or achondroplas* or Diaphyseal Aclasis or ((Hereditary or Familial or Multiple) adj2 (Exostos* or Chondrodysplas* or Osteochondroma*)) or Bessel-Hagen Disease or (Fibro* adj Dysplasia*) or Jaffe Lichtenstein* or (Marfan* adj2 (syndrome* or disease* or disorder* or abiotrophy)) or (Ehlers adj Danlos) or (Loeys adj Dietz) or (genetic adj3 aortic) or ((musc* or limb-girdle) adj dystroph*) or (Glycogen Storage Disease Type adj (II or "2")) or (glycogenos* adj2 (II or "2" or generali*)) or (Pompe adj (disease* or syndrome* or disorder*)) or porphyria* or (porphyrin adj (disorder* or disease* or syndrome*)) or hemophilia* or haemophilia* or ((cystic or pancreatic) adj2 fibros*) or mucoviscidosis or mucoviscoidosis or (pancrea* adj (fibrocystic or fibros* or cystic)) or myelomeningocele* or (neural tube adj2 defect*) or spina bifida* or (congenital adj3 (limb* or exremit*)) or ((limb* or exremit*) adj3 (deformi* or malform* or anomalit*)) or Arthrogryposis multiplex congenita or Amyoplasia* or (Charcot adj Marie) or (Roussy adj Levy) or Peroneal Muscular Atroph* or Hereditary Areflexic Dystasia* or ("hereditary motor and sensory" adj neuropat*) or (HMSN adj2 (I or II or 1A or 1B or "5" or V)) or HMSN1A or HMSN1B or ((Strumpel* or Struempel*) adj1 (disease or syndrome)) or (familial spastic adj (paralysis or paraparesis or paraplegia)) or (dejerine sottas adj (syndrome or disease)) or (myoton* adj2 (dystroph* or atrophica* or myopathy*)) or (Steinert* adj disease*) or Ricker Syndrome* or PROMM* or (Hereditary adj3 Spastic Paraplegia*) or Spastic Paraplegia Hypertrophic Motor Sensory Neuropathy or "CMT with Pyramidal Features" or (Spastic Paraplegia adj2 ("2" or II)) or SPG2 or (rare adj3 hereditary ataxia*) or ((DiGeorge or Di George) adj (syndrome* or sequence or anomaly)) or ((velocardiofacial or Velo Cardio Facial or 22q11 or vcf or pharyngeal pouch or Thymic Aplasia or Sedlackova or Shprintzen) adj2 syndrome*) or "Autosomal Dominant Opitz G Bbb Syndrome" or "Conotruncal Anomaly Face Syndrome" or Catch22 or neurofibromatos* or recklinghausen* or (multiple adj1 neurofibroma*) or ((Turner* or Ullrich* or XO or 45X) adj3 (syndrome* or disease* or state or status or stigma*)) or (Gonadal Dysgenesis adj XO) or Monosomy X or (Bonnevie Ullrich adj (syndrome* or status)) or (mitochondrial adj (disease* or disorder*)) or respiratory chain deficienc* or oxidative phosphorylation deficienc* or (Noonan* adj3 (syndrome* or disease*)) or ((Klinefelter* or XXY or XXYY or XXXXY) adj3 (syndrome* or trisom* or disease*))).tw,kw. | 400497 |
| 3 | 1 or 2 | 567804 |
| 4 | exp work/ or exp employment/ or employability/ or exp occupation/ or job stress/ or medical leave/ or absenteeism/ or presenteeism/ or retirement/ or work capacity/ or work disability/ or vocational rehabilitation/ | 647308 |
| 5 | ((work* adj3 (participat* or disabilit* or abilit* or rehabilit* or challenge* or adapt* or facilitat* or tenure or load or activ* or left or leave or leaving or sustain* or capacit* or measure or measures or protect* or inhibit* or incapacity* or qualit* or life)) or (disabilit* adj3 (pension* or rehabilit* or benefit*)) or ((disabilit* or sick*) adj3 (leave or absent*)) or retirement or retired or retiring or employ* or unemploy* or job or jobs or occupation* or vocation* or career* or workplace* or workforce* or absenteeism or presenteeism).tw,kw. | 1217485 |
| 6 | 4 or 5 | 1603370 |
| 7 | 3 and 6 | 12640 |
| 8 | limit 7 to yr="2000 - 2020" | 10243 |

**APA PsycINFO 1987 to March Week 4 2020 (Ovid)**

Date for search: 1 April 2020
Number of hits: 749

| 1 | Muscular Dystrophy/ or Porphyria/ or Hemophilia/ or Cystic Fibrosis/ or Charcot-Marie-Tooth Disease/ or exp Neurofibromatosis/ or Turners Syndrome/ or Klinefelters Syndrome/ | 18611 |
| --- | --- | --- |
| 2 | ((rare adj3 (disease* or disorder*)) or (orphan adj (disease* or disorder*)) or (osteogenesis adj imperfecta) or brittle bone disease* or fragilitas ossium or osteopsathyrosis or ((lobstein* or bruck*) adj (disease* or syndrome*)) or ((skeletal or fibrous) adj3 (dysplasia* or bone*)) or achondroplas* or Diaphyseal Aclasis or ((Hereditary or Familial or Multiple) adj2 (Exostos* or Chondrodysplas* or Osteochondroma*)) or Bessel-Hagen Disease or (Fibro* adj Dysplasia*) or Jaffe Lichtenstein* or (Marfan* adj2 (syndrome* or disease* or disorder* or abiotrophy)) or (Ehlers adj Danlos) or (Loeys adj Dietz) or (genetic adj3 aortic) or ((musc* or limb-girdle) adj dystroph*) or (Glycogen Storage Disease Type adj (II or "2")) or (glycogenos* adj2 (II or "2" or generali*)) or (Pompe adj (disease* or syndrome* or disorder*)) or porphyria* or (porphyrin adj (disorder* or disease* or syndrome*)) or hemophilia* or haemophilia* or ((cystic or pancreatic) adj2 fibros*) or mucoviscidosis or mucoviscoidosis or (pancrea* adj (fibrocystic or fibros* or cystic)) or myelomeningocele* or (neural tube adj2 defect*) or spina bifida* or (congenital adj3 (limb* or exremit*)) or ((limb* or exremit*) adj3 (deformi* or malform* or anomalit*)) or Arthrogryposis multiplex congenita or Amyoplasia* or (Charcot adj Marie) or (Roussy adj Levy) or Peroneal Muscular Atroph* or Hereditary Areflexic Dystasia* or ("hereditary motor and sensory" adj neuropat*) or (HMSN adj2 (I or II or 1A or 1B or "5" or V)) or HMSN1A or HMSN1B or ((Strumpel* or Struempel*) adj1 (disease or syndrome)) or (familial spastic adj (paralysis or paraparesis or paraplegia)) or (dejerine sottas adj (syndrome or disease)) or (myoton* adj2 (dystroph* or atrophica* or myopathy*)) or (Steinert* adj disease*) or Ricker Syndrome* or PROMM* or (Hereditary adj3 Spastic Paraplegia*) or Spastic Paraplegia Hypertrophic Motor Sensory Neuropathy or "CMT with Pyramidal Features" or (Spastic Paraplegia adj2 ("2" or II)) or SPG2 or (rare adj3 hereditary ataxia*) or ((DiGeorge or Di George) adj (syndrome* or sequence or anomaly)) or ((velocardiofacial or Velo Cardio Facial or 22q11 or vcf or pharyngeal pouch or Thymic Aplasia or Sedlackova or Shprintzen) adj2 syndrome*) or "Autosomal Dominant Opitz G Bbb Syndrome" or "Conotruncal Anomaly Face Syndrome" or Catch22 or neurofibromatos* or recklinghausen* or (multiple adj1 neurofibroma*) or ((Turner* or Ullrich* or XO or 45X) adj3 (syndrome* or disease* or state or status or stigma*)) or (Gonadal Dysgenesis adj XO) or Monosomy X or (Bonnevie Ullrich adj (syndrome* or status)) or (mitochondrial adj (disease* or disorder*)) or respiratory chain deficienc* or oxidative phosphorylation deficienc* or (Noonan* adj3 (syndrome* or disease*)) or ((Klinefelter* or XXY or XXYY or XXXXY) adj3 (syndrome* or trisom* or disease*))).tw. | 11640 |
| 3 | 1 or 2 | 25708 |
| 4 | Quality of Work Life/ or School to Work Transition/ or exp Vocational Rehabilitation/ or "work (attitudes toward)"/ or Work-Life Balance/ or Occupational Stress/ or exp Occupations/ or Workplace Intervention/ or Employee Assistance Programs/ or Employee Leave Benefits/ or exp Employee Characteristics/ or Diversity in the Workplace/ or Occupational Guidance/ | 114853 |
| 5 | ((work* adj3 (participat* or disabilit* or abilit* or rehabilit* or challenge* or adapt* or facilitat* or tenure or load or activ* or left or leave or leaving or sustain* or capacit* or measure or measures or protect* or inhibit* or incapacity* or qualit* or life)) or (disabilit* adj3 (pension* or rehabilit* or benefit*)) or ((disabilit* or sick*) adj3 (leave or absent*)) or retirement or retired or retiring or employ* or unemploy* or job or jobs or occupation* or vocation* or career* or workplace* or workforce* or absenteeism or presenteeism).tw. | 410098 |
| 6 | 4 or 5 | 428741 |
| 7 | 3 and 6 | 853 |
| 8 | limit 7 to yr="2000 - 2020" | 749 |

**AMED (Allied and Complementary Medicine) <1985 to March 2020> (Ovid)**

Date for search: 1 April 2020
Number og hits: 98

| 1 | exp Muscular Dystrophy/ or Hemophilia/ or Cystic Fibrosis/ or exp Neural Tube Defects/ or Foot Deformities Congenital/ or exp "Neuropathies Hereditary Motor and Sensory"/ or "Neuropathies Hereditary Sensory and Autonomic"/ | 1330 |
| --- | --- | --- |
| 2 | ((rare adj3 (disease* or disorder*)) or (orphan adj (disease* or disorder*)) or (osteogenesis adj imperfecta) or brittle bone disease* or fragilitas ossium or osteopsathyrosis or ((lobstein* or bruck*) adj (disease* or syndrome*)) or ((skeletal or fibrous) adj3 (dysplasia* or bone*)) or achondroplas* or Diaphyseal Aclasis or ((Hereditary or Familial or Multiple) adj2 (Exostos* or Chondrodysplas* or Osteochondroma*)) or Bessel-Hagen Disease or (Fibro* adj Dysplasia*) or Jaffe Lichtenstein* or (Marfan* adj2 (syndrome* or disease* or disorder* or abiotrophy)) or (Ehlers adj Danlos) or (Loeys adj Dietz) or (genetic adj3 aortic) or ((musc* or limb-girdle) adj dystroph*) or (Glycogen Storage Disease Type adj (II or "2")) or (glycogenos* adj2 (II or "2" or generali*)) or (Pompe adj (disease* or syndrome* or disorder*)) or porphyria* or (porphyrin adj (disorder* or disease* or syndrome*)) or hemophilia* or haemophilia* or ((cystic or pancreatic) adj2 fibros*) or mucoviscidosis or mucoviscoidosis or (pancrea* adj (fibrocystic or fibros* or cystic)) or myelomeningocele* or (neural tube adj2 defect*) or spina bifida* or (congenital adj3 (limb* or exremit*)) or ((limb* or exremit*) adj3 (deformi* or malform* or anomalit*)) or Arthrogryposis multiplex congenita or Amyoplasia* or (Charcot adj Marie) or (Roussy adj Levy) or Peroneal Muscular Atroph* or Hereditary Areflexic Dystasia* or ("hereditary motor and sensory" adj neuropat*) or (HMSN adj2 (I or II or 1A or 1B or "5" or V)) or HMSN1A or HMSN1B or ((Strumpel* or Struempel*) adj1 (disease or syndrome)) or (familial spastic adj (paralysis or paraparesis or paraplegia)) or (dejerine sottas adj (syndrome or disease)) or (myoton* adj2 (dystroph* or atrophica* or myopathy*)) or (Steinert* adj disease*) or Ricker Syndrome* or PROMM* or (Hereditary adj3 Spastic Paraplegia*) or Spastic Paraplegia Hypertrophic Motor Sensory Neuropathy or "CMT with Pyramidal Features" or (Spastic Paraplegia adj2 ("2" or II)) or SPG2 or (rare adj3 hereditary ataxia*) or ((DiGeorge or Di George) adj (syndrome* or sequence or anomaly)) or ((velocardiofacial or Velo Cardio Facial or 22q11 or vcf or pharyngeal pouch or Thymic Aplasia or Sedlackova or Shprintzen) adj2 syndrome*) or "Autosomal Dominant Opitz G Bbb Syndrome" or "Conotruncal Anomaly Face Syndrome" or Catch22 or neurofibromatos* or recklinghausen* or (multiple adj1 neurofibroma*) or ((Turner* or Ullrich* or XO or 45X) adj3 (syndrome* or disease* or state or status or stigma*)) or (Gonadal Dysgenesis adj XO) or Monosomy X or (Bonnevie Ullrich adj (syndrome* or status)) or (mitochondrial adj (disease* or disorder*)) or respiratory chain deficienc* or oxidative phosphorylation deficienc* or (Noonan* adj3 (syndrome* or disease*)) or ((Klinefelter* or XXY or XXYY or XXXXY) adj3 (syndrome* or trisom* or disease*))).mp. | 2238 |
| 3 | 1 or 2 | 2374 |
| 4 | exp Rehabilitation Vocational/ or exp Employment/ or Career Mobility/ or Career Choice/ or Job Satisfaction/ or Sick Leave/ or Absenteeism/ or Retirement/ | 7913 |
| 5 | ((work* adj3 (participat* or disabilit* or abilit* or rehabilit* or challenge* or adapt* or facilitat* or tenure or load or activ* or left or leave or leaving or sustain* or capacit* or measure or measures or protect* or inhibit* or incapacity* or qualit* or life)) or (disabilit* adj3 (pension* or rehabilit* or benefit*)) or ((disabilit* or sick*) adj3 (leave or absent*)) or retirement or retired or retiring or employ* or unemploy* or job or jobs or occupation* or vocation* or career* or workplace* or workforce* or absenteeism or presenteeism).mp. | 32797 |
| 6 | 4 or 5 | 32797 |
| 7 | 3 and 6 | 133 |
| 8 | limit 7 to yr="2000 - 2020" | 98 |

**Cochrane Library**

Date for search: 1 April 2020
Number of hits: Cochrane Database of Systematic Reviews: 18

Number og Hits: Cochrane Central Register of Controlled Trials: 228

| #1 | ([mh ^"Rare Diseases"] OR [mh ^"Osteogenesis Imperfecta"] OR [mh ^"Exostoses, Multiple Hereditary"] OR [mh "Fibrous Dysplasia of Bone"] OR [mh ^Achondroplasia] OR [mh ^"Marfan Syndrome"] OR [mh ^"Ehlers-Danlos Syndrome"] OR [mh ^"Loeys-Dietz Syndrome"] OR [mh "Muscular Dystrophies"] OR [mh ^"Glycogen Storage Disease Type II"] OR [mh Porphyrias] OR [mh ^"Hemophilia A"] OR [mh ^"Hemophilia B"] OR [mh ^"Cystic Fibrosis"] OR [mh "Neural Tube Defects"] OR [mh "Limb Deformities, Congenital"] OR [mh ^"Charcot-Marie-Tooth Disease"] OR [mh ^"Spastic Paraplegia, Hereditary"] OR [mh ^"DiGeorge Syndrome"] OR [mh Neurofibromatoses] OR [mh ^"Turner Syndrome"] OR [mh "Mitochondrial Diseases"] OR [mh ^"Noonan Syndrome"] OR [mh ^"Klinefelter Syndrome"]) | 3668 |
| --- | --- | --- |
| #2 | ((rare NEAR/2 (disease* OR disorder*)) OR (orphan NEXT (disease* OR disorder*)) OR (osteogenesis NEXT imperfecta) OR (brittle NEXT bone NEXT disease*) OR "fragilitas ossium" OR osteopsathyrosis OR ((lobstein* OR bruck*) NEXT (disease* OR syndrome*)) OR ((skeletal OR fibrous) NEAR/2 (dysplasia* OR bone*)) OR achondroplas* OR "Diaphyseal Aclasis" OR ((Hereditary OR Familial OR Multiple) NEAR/1 (Exostos* OR Chondrodysplas* OR Osteochondroma*)) OR "Bessel-Hagen Disease" OR (Fibro* NEXT Dysplasia*) OR (Jaffe NEXT Lichtenstein*) OR (Marfan* NEAR/1 (syndrome* OR disease* OR disorder* OR abiotrophy)) OR (Ehlers NEXT Danlos) OR (Loeys NEXT Dietz) OR (genetic NEAR/2 aortic) OR ((musc* OR limb-girdle) NEXT dystroph*) OR (("Glycogen Storage Disease Type") NEXT (II OR 2)) OR (glycogenos* NEAR/1 (II OR 2 OR generali*)) OR (Pompe NEXT (disease* OR syndrome* OR disorder*)) OR porphyria* OR (porphyrin NEXT (disorder* OR disease* OR syndrome*)) OR hemophilia* OR haemophilia* OR ((cystic OR pancreatic) NEAR/1 fibros*) OR mucoviscidosis OR mucoviscoidosis OR (pancrea* NEXT (fibrocystic OR fibros* OR cystic)) OR myelomeningocele* OR ("neural tube" NEAR/1 defect*) OR (spina NEXT bifida*) OR (congenital NEAR/2 (limb* OR exremit*)) OR ((limb* OR exremit*) NEAR/2 (deformi* OR malform* OR anomalit*)) OR "Arthrogryposis multiplex congenita" OR Amyoplasia* OR (Charcot NEXT Marie) OR (Roussy NEXT Levy) OR (Peroneal NEXT Muscular NEXT Atroph*) OR (Hereditary NEXT Areflexic NEXT Dystasia*) OR ("hereditary motor and sensory" NEXT neuropat*) OR (HMSN NEAR/1 (I OR II OR "1A" OR "1B" OR "5" OR V)) OR HMSN1A OR HMSN1B OR ((Strumpel* OR Struempel*) NEAR/1 (disease OR syndrome)) OR ("familial spastic" NEXT (paralysis OR paraparesis OR paraplegia)) OR ("dejerine sottas" NEXT (syndrome OR disease)) OR (myoton* NEAR/1 (dystroph* OR atrophica* OR myopathy*)) OR (Steinert* NEXT disease*) OR (Ricker NEXT (Syndrome* OR PROMM*)) OR (Hereditary NEAR/2 "Spastic Paraplegia*") OR "Spastic Paraplegia Hypertrophic Motor Sensory Neuropathy" OR "CMT with Pyramidal Features" OR ("Spastic Paraplegia" NEAR/1 ("2" OR II)) OR SPG2 OR (rare NEAR/2 (hereditary NEXT ataxia*)) OR ((DiGeorge OR "Di George") NEXT (syndrome* OR sequence OR anomaly)) OR ((velocardiofacial OR "Velo Cardio Facial" OR "22q11" OR vcf OR "pharyngeal pouch" OR "Thymic Aplasia" OR Sedlackova OR Shprintzen) NEAR/1 syndrome*) OR "Autosomal Dominant Opitz G Bbb Syndrome" OR "Conotruncal Anomaly Face Syndrome" OR "Catch22" OR neurofibromatos* OR recklinghausen* OR (multiple NEAR/1 neurofibroma*) OR ((Turner* OR Ullrich* OR XO OR "45X") NEAR/2 (syndrome* OR disease* OR state OR status OR stigma*)) OR ("Gonadal Dysgenesis" NEXT XO) OR "Monosomy X" OR ("Bonnevie Ullrich" NEXT (syndrome* OR status)) OR (mitochondrial NEXT (disease* OR disorder*)) OR (respiratory NEXT chain NEXT deficienc*) OR (oxidative NEXT phosphorylation NEXT deficienc*) OR (Noonan* NEAR/2 (syndrome* OR disease*)) OR ((Klinefelter* OR XXY OR XXYY OR XXXXY) NEAR/2 (syndrome* OR trisom* OR disease*))):ti,ab,kw | 10709 |
| #3 | #1 OR #2 | 11050 |
| #4 | ([mh Work] OR [mh ^Employment] OR [mh "Rehabilitation, Vocational"] OR [mh ^Workplace] OR [mh Occupations] OR [mh ^"Occupational Stress"] OR [mh ^"Job Satisfaction"] OR [mh ^"Work-Life Balance"] OR [mh ^"Sick Leave"] OR [mh ^Absenteeism] OR [mh ^Retirement] OR [mh ^"Vocational Guidance"] OR [mh ^"Career Choice"] OR [mh ^"Work Capacity Evaluation"] OR [mh ^Presenteeism]) | 3899 |
| #5 | ((work* NEAR/2 (participat* OR disabilit* OR abilit* OR rehabilit* OR challenge* OR adapt* OR facilitat* OR tenure OR load OR activ* OR left OR leave OR leaving OR sustain* OR capacit* OR measure OR measures OR protect* OR inhibit* OR incapacity* OR qualit* OR life)) OR (disabilit* NEAR/2 (pension* OR rehabilit* OR benefit*)) OR ((disabilit* OR sick*) NEAR/2 (leave OR absent*)) OR retirement OR retired OR retiring OR *employ* OR job OR jobs OR occupation* OR vocation* OR career* OR workplace* OR workforce* OR absenteeism OR presenteeism):ti,ab,kw | 44625 |
| #6 | #4 OR #5 | 45080 |
| #7 | #3 AND #6 | 247 |
| #8 | #7 in Cochrane Reviews, Cochrane Protocols | 18 |
| #9 | #7 in Trials | 228 |

**CINAHL (EBSCO)**

Date for search: 1 April 2020
Number of hits: 452

| S1 | (MH "Rare Diseases" OR MH "Osteogenesis Imperfecta" OR MH "Fibrous Dysplasia of Bone+" OR MH "Achondroplasia" OR MH "Marfan Syndrome" OR MH "Ehlers-Danlos Syndrome" OR MH "Loeys-Dietz Syndrome" OR MH "Muscular Dystrophy+" OR MH "Glycogen Storage Disease" OR MH "Porphyrias+" OR MH "Hemophilia+" OR MH "Cystic Fibrosis" OR MH "Neural Tube Defects+" OR MH "Limb Deformities, Congenital+" OR MH "Neuropathies, Hereditary Motor and Sensory+" OR MH "DiGeorge Syndrome" OR MH "Neurofibromatoses+" OR MH "Turner's Syndrome" OR MH "Mitochondrial Diseases+" OR MH "Noonan Syndrome" OR MH "Klinefelter's Syndrome") | 32,499 |
| --- | --- | --- |
| S2 | ((rare N1 (disease* OR disorder*)) OR (orphan N0 (disease* OR disorder*)) OR "osteogenesis imperfecta" OR "brittle bone disease*" OR "fragilitas ossium" OR osteopsathyrosis OR ((lobstein* OR bruck*) N0 (disease* OR syndrome*)) OR ((skeletal OR fibrous) N1 (dysplasia* OR bone*)) OR achondroplas* OR "Diaphyseal Aclasis" OR ((Hereditary OR Familial OR Multiple) N1 (Exostos* OR Chondrodysplas* OR Osteochondroma*)) OR "Bessel-Hagen Disease" OR ("Fibro* N0 Dysplasia*) OR "Jaffe Lichtenstein*" OR (Marfan* N1 (syndrome* OR disease* OR disorder* OR abiotrophy)) OR "Ehlers Danlos" OR "Loeys Dietz" OR (genetic N1 aortic) OR ((musc* OR limb-girdle) N0 dystroph*) OR (("Glycogen Storage Disease Type") N0 (II OR 2)) OR (glycogenos* N1 (II OR 2 OR generali*)) OR (Pompe N0 (disease* OR syndrome* OR disorder*)) OR porphyria* OR (porphyrin N0 (disorder* OR disease* OR syndrome*)) OR hemophilia* OR haemophilia* OR ((cystic OR pancreatic) N1 fibros*) OR mucoviscidosis OR mucoviscoidosis OR (pancrea* N0 (fibrocystic OR fibros* OR cystic)) OR myelomeningocele* OR ("neural tube" N1 defect*) OR "spina bifida*" OR (congenital N1 (limb* OR exremit*)) OR ((limb* OR exremit*) N1 (deformi* OR malform* OR anomalit*)) OR "Arthrogryposis multiplex congenita" OR Amyoplasia* OR "Charcot Marie" OR "Roussy Levy" OR "Peroneal Muscular Atroph*" OR "Hereditary Areflexic Dystasia*" OR ("hereditary motor and sensory" N0 neuropat*) OR (HMSN N1 (I OR II OR "1A" OR "1B" OR "5" OR V)) OR HMSN1A OR HMSN1B OR ((Strumpel* OR Struempel*) N1 (disease OR syndrome)) OR ("familial spastic" N0 (paralysis OR paraparesis OR paraplegia)) OR ("dejerine sottas" N0 (syndrome OR disease)) OR (myoton* N1 (dystroph* OR atrophica* OR myopathy*)) OR "Steinert* disease*" OR "Ricker Syndrome*" OR PROMM* OR (Hereditary N1 "Spastic Paraplegia*") OR "Spastic Paraplegia Hypertrophic Motor Sensory Neuropathy" OR "CMT with Pyramidal Features" OR ("Spastic Paraplegia" N1 ("2" OR II)) OR SPG2 OR (rare N1 "hereditary ataxia*") OR ((DiGeorge OR "Di George") N0 (syndrome* OR sequence OR anomaly)) OR ((velocardiofacial OR "Velo Cardio Facial" OR "22q11" OR vcf OR "pharyngeal pouch" OR "Thymic Aplasia" OR Sedlackova OR Shprintzen) N1 syndrome*) OR "Autosomal Dominant Opitz G Bbb Syndrome" OR "Conotruncal Anomaly Face Syndrome" OR Catch22 OR neurofibromatos* OR recklinghausen* OR (multiple N1 neurofibroma*) OR ((Turner* OR Ullrich* OR XO OR S45 X) N1 (syndrome* OR disease* OR state OR status OR stigma*)) OR ("Gonadal Dysgenesis" N0 XO) OR "Monosomy X" OR ("Bonnevie Ullrich" N0 (syndrome* OR status)) OR (mitochondrial N0 (disease* OR disorder*)) OR "respiratory chain deficienc*" OR "oxidative phosphorylation deficienc*" OR (Noonan* N1 (syndrome* OR disease*)) OR ((Klinefelter* OR XXY OR XXYY OR XXXXY) N1 (syndrome* OR trisom* OR disease*))) | 14,027 |
| S3 | S1 OR S2 | 43,202 |
| S4 | (MH "Work+" MH "Employment+" OR MH "Rehabilitation, Vocational" OR MH "Work Environment+" OR MH "Occupations and Professions+" OR MH "Stress, Occupational" OR MH "Job Satisfaction" OR MH "Work-Life Balance" OR MH "Sick Leave" OR MH "Absenteeism" OR MH "Presenteeism" OR MH "Vocational Guidance" OR MH "Retirement" OR MH "Career Planning and Development" OR MH "Work Capacity Evaluation") | 184,355 |
| S5 | ((work* N1 (participat* OR disabilit* OR abilit* OR rehabilit* OR challenge* OR adapt* OR facilitat* OR tenure OR load OR activ* OR left OR leave OR leaving OR sustain* OR capacit* OR measure OR measures OR protect* OR inhibit* OR incapacity* OR qualit* OR life)) OR (disabilit* N1 (pension* OR rehabilit* OR benefit*)) OR ((disabilit* OR sick*) N1 (leave OR absent*)) OR retirement OR retired OR retiring OR employ* OR unemploy* OR job OR jobs OR occupation* OR vocation* OR career* OR workplace* OR ... | 529,215 |
| S6 | S4 OR S5 | 580,374 |
| S7 | S3 AND S6 | 1,027 |
| S8 | S3 AND S6 Limiters - Published Date: 20000101-20201231; Exclude MEDLINE records | 452 |

**Scopus**

Date for search: 1 April 2020
Number of hits: 6056

TITLE-ABS-KEY ( ( ( ( rare W/2 ( disease* OR disorder* ) ) OR ( orphan W/0 ( disease* OR disorder* ) ) OR ( "osteogenesis imperfecta" ) OR "brittle bone disease*" OR "fragilitas ossium" OR osteopsathyrosis OR ( ( lobstein* OR bruck* ) W/0 ( disease* OR syndrome* ) ) OR ( ( skeletal OR fibrous ) W/2 ( dysplasia* OR bone* ) ) OR achondroplas* OR "Diaphyseal Aclasis" OR ( ( hereditary OR familial OR multiple ) W/1 ( exostos* OR chondrodysplas* OR osteochondroma* ) ) OR "Bessel-Hagen Disease" OR ( fibro* W/0 dysplasia* ) OR "Jaffe Lichtenstein*" OR ( marfan* W/1 ( syndrome* OR disease* OR disorder* OR abiotrophy ) ) OR "Ehlers Danlos" OR "Loeys Dietz" OR ( genetic W/2 aortic ) OR ( ( musc* OR limb-girdle ) W/0 dystroph* ) OR ( "Glycogen Storage Disease Type" W/0 ( ii OR "2" ) ) OR ( glycogenos* W/1 ( ii OR "2" OR generali* ) ) OR ( pompe W/0 ( disease* OR syndrome* OR disorder* ) ) OR porphyria* OR ( porphyrin W/0 ( disorder* OR disease* OR syndrome* ) ) OR hemophilia* OR haemophilia* OR ( ( cystic OR pancreatic ) W/1 fibros* ) OR mucoviscidosis OR mucoviscoidosis OR ( pancrea* W/0 ( fibrocystic OR fibros* OR cystic ) ) OR myelomeningocele* OR ( neural AND tube W/1 defect* ) OR "spina bifida*" OR ( congenital W/2 ( limb* OR exremit* ) ) OR ( ( limb* OR exremit* ) W/2 ( deformi* OR malform* OR anomalit* ) ) OR "Arthrogryposis multiplex congenita OR Amyoplasia*" OR "Charcot Marie" OR "Roussy Levy" OR "Peroneal Muscular Atroph*" OR "Hereditary Areflexic Dystasia*" OR ( "hereditary motor and sensory" W/0 neuropat* ) OR ( hmsn W/1 ( i OR ii OR 1a OR 1b OR 5 OR v ) ) OR hmsn1a OR hmsn1b OR ( ( strumpel* OR struempel* ) W/0 ( disease OR syndrome ) ) OR ( familial AND spastic W/0 ( paralysis OR paraparesis OR paraplegia ) ) OR ( "dejerine sottas" W/0 ( syndrome OR disease ) ) OR ( myoton* W/1 ( dystroph* OR atrophica* OR myopathy* ) ) OR ( steinert* W/0 disease* ) OR "Ricker Syndrome*" OR promm* OR ( hereditary W/2 "Spastic Paraplegia*" ) OR "Spastic Paraplegia Hypertrophic Motor Sensory Neuropathy" OR "CMT with Pyramidal Features" OR ( "Spastic Paraplegia" W/1 ( 2 OR ii ) ) OR spg2 OR ( rare W/2 "hereditary ataxia*" ) OR ( ( digeorge OR "Di George" ) W/0 ( syndrome* OR sequence OR anomaly ) ) OR ( ( velocardiofacial OR "Velo Cardio Facial" OR 22q11 OR vcf OR "pharyngeal pouch" OR "Thymic Aplasia" OR sedlackova OR shprintzen ) W/1 syndrome* ) OR "Autosomal Dominant Opitz G Bbb Syndrome" OR "Conotruncal Anomaly Face Syndrome" OR catch22 OR neurofibromatos* OR recklinghausen* OR ( multiple W/0 neurofibroma* ) OR ( ( turner* OR ullrich* OR xo OR 45x ) W/2 ( syndrome* OR disease* OR state OR status OR stigma* ) ) OR ( "Gonadal Dysgenesis" W/0 xo ) OR "Monosomy X" OR ( "Bonnevie Ullrich" W/0 ( syndrome* OR status ) ) OR ( mitochondrial W/0 ( disease* OR disorder* ) ) OR "respiratory chain deficienc*" OR "oxidative phosphorylation deficienc*" OR ( noonan* W/2 ( syndrome* OR disease* ) ) OR ( ( klinefelter* OR xxy OR xxyy OR xxxxy ) W/2 ( syndrome* OR trisom* OR disease* ) ) ) AND ( ( work* W/2 ( participat* OR disabilit* OR abilit* OR rehabilit* OR challenge* OR adapt* OR facilitat* OR tenure OR load OR activ* OR left OR leave OR leaving OR sustain* OR capacit* OR measure OR measures OR protect* OR inhibit* OR incapacity* OR qualit* OR life ) ) OR ( disabilit* W/2 ( pension* OR rehabilit* OR benefit* ) ) OR ( ( disabilit* OR sick* ) W/2 ( leave OR absent* ) ) OR retirement OR retired OR retiring OR employ* OR unemploy* OR job OR jobs OR occupation* OR vocation* OR career* OR workplace* OR workforce* OR absenteeism OR presenteeism ) ) ) AND ( LIMIT-TO ( PUBYEAR , 2020 ) OR LIMIT-TO ( PUBYEAR , 2019 ) OR LIMIT-TO ( PUBYEAR , 2018 ) OR LIMIT-TO ( PUBYEAR , 2017 ) OR LIMIT-TO ( PUBYEAR , 2016 ) OR LIMIT-TO ( PUBYEAR , 2015 ) OR LIMIT-TO ( PUBYEAR , 2014 ) OR LIMIT-TO ( PUBYEAR , 2013 ) OR LIMIT-TO ( PUBYEAR , 2012 ) OR LIMIT-TO ( PUBYEAR , 2011 ) OR LIMIT-TO ( PUBYEAR , 2010 ) OR LIMIT-TO ( PUBYEAR , 2009 ) OR LIMIT-TO ( PUBYEAR , 2008 ) OR LIMIT-TO ( PUBYEAR , 2007 ) OR LIMIT-TO ( PUBYEAR , 2006 ) OR LIMIT-TO ( PUBYEAR , 2005 ) OR LIMIT-TO ( PUBYEAR , 2004 ) OR LIMIT-TO ( PUBYEAR , 2003 ) OR LIMIT-TO ( PUBYEAR , 2002 ) OR LIMIT-TO ( PUBYEAR , 2001 ) OR LIMIT-TO ( PUBYEAR , 2000 ) )

**Web of Science Core Collection**

Date for search: 1 April 2020
Number of hits: 4071

TOPIC: (((rare NEAR/2 (disease* OR disorder*)) OR (orphan NEAR/0 (disease* OR disorder*)) OR ("osteogenesis imperfecta") OR "brittle bone disease*" OR "fragilitas ossium" OR osteopsathyrosis OR ((lobstein* OR bruck*) NEAR/0 (disease* OR syndrome*)) OR ((skeletal OR fibrous) NEAR/2 (dysplasia* OR bone*)) OR achondroplas* OR "Diaphyseal Aclasis" OR ((Hereditary OR Familial OR Multiple) NEAR/1 (Exostos* OR Chondrodysplas* OR Osteochondroma*)) OR "Bessel-Hagen Disease" OR (Fibro* NEAR/0 Dysplasia*) OR "Jaffe Lichtenstein*" OR (Marfan* NEAR/1 (syndrome* OR disease* OR disorder* OR abiotrophy)) OR "Ehlers Danlos" OR "Loeys Dietz" OR (genetic NEAR/2 aortic) OR ((musc* OR limb-girdle) NEAR/0 dystroph*) OR ("Glycogen Storage Disease Type" NEAR/0 (II OR "2")) OR (glycogenos* NEAR/1 (II OR "2" OR generali*)) OR (Pompe NEAR/0 (disease* OR syndrome* OR disorder*)) OR porphyria* OR (porphyrin NEAR/0 (disorder* OR disease* OR syndrome*)) OR hemophilia* OR haemophilia* OR ((cystic OR pancreatic) NEAR/1 fibros*) OR mucoviscidosis OR mucoviscoidosis OR (pancrea* NEAR/0 (fibrocystic OR fibros* OR cystic)) OR myelomeningocele* OR (neural tube NEAR/1 defect*) OR "spina bifida*" OR (congenital NEAR/2 (limb* OR exremit*)) OR ((limb* OR exremit*) NEAR/2 (deformi* OR malform* OR anomalit*)) OR "Arthrogryposis multiplex congenita OR Amyoplasia*" OR "Charcot Marie" OR "Roussy Levy" OR "Peroneal Muscular Atroph*" OR "Hereditary Areflexic Dystasia*" OR ("hereditary motor and sensory" NEAR/0 neuropat*) OR (HMSN NEAR/1 (I OR II OR 1A OR 1B OR 5 OR V)) OR HMSN1A OR HMSN1B OR ((Strumpel* OR Struempel*) NEAR/0 (disease OR syndrome)) OR (familial spastic NEAR/0 (paralysis OR paraparesis OR paraplegia)) OR ("dejerine sottas" NEAR/0 (syndrome OR disease)) OR (myoton* NEAR/1 (dystroph* OR atrophica* OR myopathy*)) OR (Steinert* NEAR/0 disease*) OR "Ricker Syndrome*" OR PROMM* OR (Hereditary NEAR/2 "Spastic Paraplegia*") OR "Spastic Paraplegia Hypertrophic Motor Sensory Neuropathy" OR "CMT with Pyramidal Features" OR ("Spastic Paraplegia" NEAR/1 (2 OR II)) OR SPG2 OR (rare NEAR/2 "hereditary ataxia*") OR ((DiGeorge OR "Di George") NEAR/0 (syndrome* OR sequence OR anomaly)) OR ((velocardiofacial OR "Velo Cardio Facial" OR 22q11 OR vcf OR "pharyngeal pouch" OR "Thymic Aplasia" OR Sedlackova OR Shprintzen) NEAR/1 syndrome*) OR "Autosomal Dominant Opitz G Bbb Syndrome" OR "Conotruncal Anomaly Face Syndrome" OR Catch22 OR neurofibromatos* OR recklinghausen* OR (multiple NEAR/0 neurofibroma*) OR ((Turner* OR Ullrich* OR XO OR 45X) NEAR/2 (syndrome* OR disease* OR state OR status OR stigma*)) OR ("Gonadal Dysgenesis" NEAR/0 XO) OR "Monosomy X" OR ("Bonnevie Ullrich" NEAR/0 (syndrome* OR status)) OR (mitochondrial NEAR/0 (disease* OR disorder*)) OR "respiratory chain deficienc*" OR "oxidative phosphorylation deficienc*" OR (Noonan* NEAR/2 (syndrome* OR disease*)) OR ((Klinefelter* OR XXY OR XXYY OR XXXXY) NEAR/2 (syndrome* OR trisom* OR disease*))) AND ((work* NEAR/2 (participat* OR disabilit* OR abilit* OR rehabilit* OR challenge* OR adapt* OR facilitat* OR tenure OR load OR activ* OR left OR leave OR leaving OR sustain* OR capacit* OR measure OR measures OR protect* OR inhibit* OR incapacity* OR qualit* OR life)) OR (disabilit* NEAR/2 (pension* OR rehabilit* OR benefit*)) OR ((disabilit* OR sick*) NEAR/2 (leave OR absent*)) OR retirement OR retired OR retiring OR employ* OR unemploy* OR job OR jobs OR occupation* OR vocation* OR career* OR workplace* OR workforce* OR absenteeism OR presenteeism))

Indexes=SCI-EXPANDED, SSCI, A&HCI, CPCI-S, CPCI-SSH, ESCI Timespan=2000-2020

**SveMed+**

Date for search: 1 April 2020
Number of hits: 8

| 1 | (noexp:"Rare Diseases" OR noexp:"Osteogenesis Imperfecta" OR noexp:"Exostoses, Multiple Hereditary" OR exp:"Fibrous Dysplasia of Bone" OR noexp:"Achondroplasia" OR noexp:"Marfan Syndrome" OR noexp:"Ehlers-Danlos Syndrome" OR noexp:"Loeys-Dietz Syndrome" OR exp:"Muscular Dystrophies" OR noexp:"Glycogen Storage Disease Type II" OR exp:"Porphyrias" OR noexp:"Hemophilia A" OR noexp:"Hemophilia B" OR noexp:"Cystic Fibrosis" OR exp:"Neural Tube Defects" OR exp:"Limb Deformities, Congenital" OR noexp:"Charcot-Marie-Tooth Disease" OR noexp:"Spastic Paraplegia, Hereditary" OR noexp:"DiGeorge Syndrome" OR exp:"Neurofibromatoses" OR noexp:"Turner Syndrome" OR exp:"Mitochondrial Diseases" OR noexp:"Noonan Syndrome" OR noexp:"Klinefelter Syndrome") | 879 |
| --- | --- | --- |
| 2 | (exp:"Work" OR noexp:"Employment" OR exp:"Rehabilitation, Vocational" OR noexp:"Workplace" OR exp:"Occupations" OR noexp:"Occupational Stress" OR noexp:"Job Satisfaction" OR noexp:"Work-Life Balance" OR noexp:"Sick Leave" OR noexp:"Absenteeism" OR noexp:"Presenteeism" OR noexp:"Retirement" OR noexp:"Vocational Guidance" OR noexp:"Career Choice" OR noexp:"Work Capacity Evaluation") | 4454 |
| 3 | #1 AND #2 | 8 |
