# Supplementary material for: Work participation in adults with rare genetic diseases - a scoping review
Source: BMC Public Health. 2023 May 19;23:910. doi: 10.1186/s12889-023-15654-3 (PMC10197424; doi:10.1186/s12889-023-15654-3)
Supplement: Supplementary file 3 — Search strategies [file 12889_2023_15654_MOESM3_ESM.docx]

**Supplementary file 3: Search strategies**

All searches were performed on 27 September 2021. Exeption: SveMed+ was searched on 1 April 2020, SveMed+ has not been updated since December 2019.

**Ovid MEDLINE(R) ALL <1946 to September 24, 2021>**

| 1 | Rare Diseases/ or Osteogenesis Imperfecta/ or Exostoses, Multiple Hereditary/ or exp Fibrous Dysplasia of Bone/ or Achondroplasia/ or Marfan Syndrome/ or Ehlers-Danlos Syndrome/ or Loeys-Dietz Syndrome/ or exp Muscular Dystrophies/ or Glycogen Storage Disease Type II/ or exp Porphyrias/ or Hemophilia A/ or Hemophilia B/ or Cystic Fibrosis/ or exp Neural Tube Defects/ or exp Limb Deformities, Congenital/ or Charcot-Marie-Tooth Disease/ or Spastic Paraplegia, Hereditary/ or DiGeorge Syndrome/ or exp Neurofibromatoses/ or Turner Syndrome/ or exp Mitochondrial Diseases/ or Noonan Syndrome/ or Klinefelter Syndrome/ | 234268 |
| --- | --- | --- |
| 2 | ((rare adj3 (disease* or disorder*)) or (orphan adj (disease* or disorder*)) or (osteogenesis adj imperfecta) or brittle bone disease* or fragilitas ossium or osteopsathyrosis or ((lobstein* or bruck*) adj (disease* or syndrome*)) or ((skeletal or fibrous) adj3 (dysplasia* or bone*)) or achondroplas* or Diaphyseal Aclasis or ((Hereditary or Familial or Multiple) adj2 (Exostos* or Chondrodysplas* or Osteochondroma*)) or Bessel-Hagen Disease or (Fibro* adj Dysplasia*) or Jaffe Lichtenstein* or (Marfan* adj2 (syndrome* or disease* or disorder* or abiotrophy)) or (Ehlers adj Danlos) or (Loeys adj Dietz) or (genetic adj3 aortic) or ((musc* or limb-girdle) adj dystroph*) or (Glycogen Storage Disease Type adj (II or "2")) or (glycogenos* adj2 (II or "2" or generali*)) or (Pompe adj (disease* or syndrome* or disorder*)) or porphyria* or (porphyrin adj (disorder* or disease* or syndrome*)) or hemophilia* or haemophilia* or ((cystic or pancreatic) adj2 fibros*) or mucoviscidosis or mucoviscoidosis or (pancrea* adj (fibrocystic or fibros* or cystic)) or myelomeningocele* or (neural tube adj2 defect*) or spina bifida* or (congenital adj3 (limb* or extremit*)) or ((limb* or extremit*) adj3 (deformi* or malform* or anomalit*)) or Arthrogryposis multiplex congenita or Amyoplasia* or (Charcot adj Marie) or (Roussy adj Levy) or Peroneal Muscular Atroph* or Hereditary Areflexic Dystasia* or ("hereditary motor and sensory" adj neuropat*) or (HMSN adj2 (I or II or 1A or 1B or "5" or V)) or HMSN1A or HMSN1B or ((Strumpel* or Struempel*) adj1 (disease or syndrome)) or (familial spastic adj (paralysis or paraparesis or paraplegia)) or (dejerine sottas adj (syndrome or disease)) or (myoton* adj2 (dystroph* or atrophica* or myopathy*)) or (Steinert* adj disease*) or Ricker Syndrome* or PROMM* or (Hereditary adj3 Spastic Paraplegia*) or Spastic Paraplegia Hypertrophic Motor Sensory Neuropathy or "CMT with Pyramidal Features" or (Spastic Paraplegia adj2 ("2" or II)) or SPG2 or (rare adj3 hereditary ataxia*) or ((DiGeorge or Di George) adj (syndrome* or sequence or anomaly)) or ((velocardiofacial or Velo Cardio Facial or 22q11 or vcf or pharyngeal pouch or Thymic Aplasia or Sedlackova or Shprintzen) adj2 syndrome*) or "Autosomal Dominant Opitz G Bbb Syndrome" or "Conotruncal Anomaly Face Syndrome" or Catch22 or neurofibromatos* or recklinghausen* or (multiple adj1 neurofibroma*) or ((Turner* or Ullrich* or XO or 45X) adj3 (syndrome* or disease* or state or status or stigma*)) or (Gonadal Dysgenesis adj XO) or Monosomy X or (Bonnevie Ullrich adj (syndrome* or status)) or (mitochondrial adj (disease* or disorder*)) or respiratory chain deficienc* or oxidative phosphorylation deficienc* or (Noonan* adj3 (syndrome* or disease*)) or ((Klinefelter* or XXY or XXYY or XXXXY) adj3 (syndrome* or trisom* or disease*))).tw,kf. | 293871 |
| 3 | 1 or 2 | 380434 |
| 4 | exp Work/ or Employment/ or exp Rehabilitation, Vocational/ or Workplace/ or exp Occupations/ or Occupational Stress/ or Job Satisfaction/ or Work-Life Balance/ or Sick Leave/ or Absenteeism/ or Retirement/ or Vocational Guidance/ or Career Choice/ or Work Capacity Evaluation/ or Presenteeism/ | 231730 |
| 5 | ((work* adj3 (participat* or disabilit* or abilit* or rehabilit* or challenge* or adapt* or facilitat* or tenure or load or activ* or left or leave or leaving or sustain* or capacit* or measure or measures or protect* or inhibit* or incapacity* or qualit* or life)) or (disabilit* adj3 (pension* or rehabilit* or benefit*)) or ((disabilit* or sick*) adj3 (leave or absent*)) or retirement or retired or retiring or employ* or unemploy* or job or jobs or occupation* or vocation* or career* or workplace* or workforce* or absenteeism or presenteeism).tw,kf. | 1046679 |
| 6 | 4 or 5 | 1158640 |
| 7 | 3 and 6 | 6041 |
| 8 | limit 7 to yr="2000 - 2021" | 4473 |

**Embase Classic+Embase <1947 to 2021 September 24>**

| 1 | rare disease/ or osteogenesis imperfecta/ or hereditary multiple exostosis/ or exp fibrous dysplasia/ or achondroplasia/ or Marfan syndrome/ or Ehlers Danlos syndrome/ or Loeys Dietz syndrome/ or exp muscular dystrophy/ or glycogen storage disease type 2/ or exp porphyria/ or exp hemophilia/ or cystic fibrosis/ or exp neural tube defect/ or exp limb malformation/ or hereditary motor sensory neuropathy/ or DiGeorge syndrome/ or exp neurofibromatosis/ or exp disorders of mitochondrial functions/ or Noonan syndrome/ or exp Klinefelter syndrome/ | 450434 |
| --- | --- | --- |
| 2 | ((rare adj3 (disease* or disorder*)) or (orphan adj (disease* or disorder*)) or (osteogenesis adj imperfecta) or brittle bone disease* or fragilitas ossium or osteopsathyrosis or ((lobstein* or bruck*) adj (disease* or syndrome*)) or ((skeletal or fibrous) adj3 (dysplasia* or bone*)) or achondroplas* or Diaphyseal Aclasis or ((Hereditary or Familial or Multiple) adj2 (Exostos* or Chondrodysplas* or Osteochondroma*)) or Bessel-Hagen Disease or (Fibro* adj Dysplasia*) or Jaffe Lichtenstein* or (Marfan* adj2 (syndrome* or disease* or disorder* or abiotrophy)) or (Ehlers adj Danlos) or (Loeys adj Dietz) or (genetic adj3 aortic) or ((musc* or limb-girdle) adj dystroph*) or (Glycogen Storage Disease Type adj (II or "2")) or (glycogenos* adj2 (II or "2" or generali*)) or (Pompe adj (disease* or syndrome* or disorder*)) or porphyria* or (porphyrin adj (disorder* or disease* or syndrome*)) or hemophilia* or haemophilia* or ((cystic or pancreatic) adj2 fibros*) or mucoviscidosis or mucoviscoidosis or (pancrea* adj (fibrocystic or fibros* or cystic)) or myelomeningocele* or (neural tube adj2 defect*) or spina bifida* or (congenital adj3 (limb* or extremit*)) or ((limb* or extremit*) adj3 (deformi* or malform* or anomalit*)) or Arthrogryposis multiplex congenita or Amyoplasia* or (Charcot adj Marie) or (Roussy adj Levy) or Peroneal Muscular Atroph* or Hereditary Areflexic Dystasia* or ("hereditary motor and sensory" adj neuropat*) or (HMSN adj2 (I or II or 1A or 1B or "5" or V)) or HMSN1A or HMSN1B or ((Strumpel* or Struempel*) adj1 (disease or syndrome)) or (familial spastic adj (paralysis or paraparesis or paraplegia)) or (dejerine sottas adj (syndrome or disease)) or (myoton* adj2 (dystroph* or atrophica* or myopathy*)) or (Steinert* adj disease*) or Ricker Syndrome* or PROMM* or (Hereditary adj3 Spastic Paraplegia*) or Spastic Paraplegia Hypertrophic Motor Sensory Neuropathy or "CMT with Pyramidal Features" or (Spastic Paraplegia adj2 ("2" or II)) or SPG2 or (rare adj3 hereditary ataxia*) or ((DiGeorge or Di George) adj (syndrome* or sequence or anomaly)) or ((velocardiofacial or Velo Cardio Facial or 22q11 or vcf or pharyngeal pouch or Thymic Aplasia or Sedlackova or Shprintzen) adj2 syndrome*) or "Autosomal Dominant Opitz G Bbb Syndrome" or "Conotruncal Anomaly Face Syndrome" or Catch22 or neurofibromatos* or recklinghausen* or (multiple adj1 neurofibroma*) or ((Turner* or Ullrich* or XO or 45X) adj3 (syndrome* or disease* or state or status or stigma*)) or (Gonadal Dysgenesis adj XO) or Monosomy X or (Bonnevie Ullrich adj (syndrome* or status)) or (mitochondrial adj (disease* or disorder*)) or respiratory chain deficienc* or oxidative phosphorylation deficienc* or (Noonan* adj3 (syndrome* or disease*)) or ((Klinefelter* or XXY or XXYY or XXXXY) adj3 (syndrome* or trisom* or disease*))).tw,kw. | 433223 |
| 3 | 1 or 2 | 619096 |
| 4 | exp work/ or exp employment/ or employability/ or exp occupation/ or job stress/ or medical leave/ or absenteeism/ or presenteeism/ or retirement/ or work capacity/ or work disability/ or vocational rehabilitation/ | 717034 |
| 5 | ((work* adj3 (participat* or disabilit* or abilit* or rehabilit* or challenge* or adapt* or facilitat* or tenure or load or activ* or left or leave or leaving or sustain* or capacit* or measure or measures or protect* or inhibit* or incapacity* or qualit* or life)) or (disabilit* adj3 (pension* or rehabilit* or benefit*)) or ((disabilit* or sick*) adj3 (leave or absent*)) or retirement or retired or retiring or employ* or unemploy* or job or jobs or occupation* or vocation* or career* or workplace* or workforce* or absenteeism or presenteeism).tw,kw. | 1356481 |
| 6 | 4 or 5 | 1778306 |
| 7 | 3 and 6 | 14417 |
| 8 | limit 7 to yr="2000 - 2021" | 12003 |

**APA PsycInfo <1987 to September Week 3 2021>**

| 1 | Muscular Dystrophy/ or Porphyria/ or Hemophilia/ or Cystic Fibrosis/ or Charcot-Marie-Tooth Disease/ or exp Neurofibromatosis/ or Turners Syndrome/ or Klinefelters Syndrome/ | 20166 |
| --- | --- | --- |
| 2 | ((rare adj3 (disease* or disorder*)) or (orphan adj (disease* or disorder*)) or (osteogenesis adj imperfecta) or brittle bone disease* or fragilitas ossium or osteopsathyrosis or ((lobstein* or bruck*) adj (disease* or syndrome*)) or ((skeletal or fibrous) adj3 (dysplasia* or bone*)) or achondroplas* or Diaphyseal Aclasis or ((Hereditary or Familial or Multiple) adj2 (Exostos* or Chondrodysplas* or Osteochondroma*)) or Bessel-Hagen Disease or (Fibro* adj Dysplasia*) or Jaffe Lichtenstein* or (Marfan* adj2 (syndrome* or disease* or disorder* or abiotrophy)) or (Ehlers adj Danlos) or (Loeys adj Dietz) or (genetic adj3 aortic) or ((musc* or limb-girdle) adj dystroph*) or (Glycogen Storage Disease Type adj (II or "2")) or (glycogenos* adj2 (II or "2" or generali*)) or (Pompe adj (disease* or syndrome* or disorder*)) or porphyria* or (porphyrin adj (disorder* or disease* or syndrome*)) or hemophilia* or haemophilia* or ((cystic or pancreatic) adj2 fibros*) or mucoviscidosis or mucoviscoidosis or (pancrea* adj (fibrocystic or fibros* or cystic)) or myelomeningocele* or (neural tube adj2 defect*) or spina bifida* or (congenital adj3 (limb* or exremit*)) or ((limb* or exremit*) adj3 (deformi* or malform* or anomalit*)) or Arthrogryposis multiplex congenita or Amyoplasia* or (Charcot adj Marie) or (Roussy adj Levy) or Peroneal Muscular Atroph* or Hereditary Areflexic Dystasia* or ("hereditary motor and sensory" adj neuropat*) or (HMSN adj2 (I or II or 1A or 1B or "5" or V)) or HMSN1A or HMSN1B or ((Strumpel* or Struempel*) adj1 (disease or syndrome)) or (familial spastic adj (paralysis or paraparesis or paraplegia)) or (dejerine sottas adj (syndrome or disease)) or (myoton* adj2 (dystroph* or atrophica* or myopathy*)) or (Steinert* adj disease*) or Ricker Syndrome* or PROMM* or (Hereditary adj3 Spastic Paraplegia*) or Spastic Paraplegia Hypertrophic Motor Sensory Neuropathy or "CMT with Pyramidal Features" or (Spastic Paraplegia adj2 ("2" or II)) or SPG2 or (rare adj3 hereditary ataxia*) or ((DiGeorge or Di George) adj (syndrome* or sequence or anomaly)) or ((velocardiofacial or Velo Cardio Facial or 22q11 or vcf or pharyngeal pouch or Thymic Aplasia or Sedlackova or Shprintzen) adj2 syndrome*) or "Autosomal Dominant Opitz G Bbb Syndrome" or "Conotruncal Anomaly Face Syndrome" or Catch22 or neurofibromatos* or recklinghausen* or (multiple adj1 neurofibroma*) or ((Turner* or Ullrich* or XO or 45X) adj3 (syndrome* or disease* or state or status or stigma*)) or (Gonadal Dysgenesis adj XO) or Monosomy X or (Bonnevie Ullrich adj (syndrome* or status)) or (mitochondrial adj (disease* or disorder*)) or respiratory chain deficienc* or oxidative phosphorylation deficienc* or (Noonan* adj3 (syndrome* or disease*)) or ((Klinefelter* or XXY or XXYY or XXXXY) adj3 (syndrome* or trisom* or disease*))).tw. | 12407 |
| 3 | 1 or 2 | 27779 |
| 4 | Quality of Work Life/ or School to Work Transition/ or exp Vocational Rehabilitation/ or "work (attitudes toward)"/ or Work-Life Balance/ or Occupational Stress/ or exp Occupations/ or Workplace Intervention/ or Employee Assistance Programs/ or Employee Leave Benefits/ or exp Employee Characteristics/ or Diversity in the Workplace/ or Occupational Guidance/ | 126309 |
| 5 | ((work* adj3 (participat* or disabilit* or abilit* or rehabilit* or challenge* or adapt* or facilitat* or tenure or load or activ* or left or leave or leaving or sustain* or capacit* or measure or measures or protect* or inhibit* or incapacity* or qualit* or life)) or (disabilit* adj3 (pension* or rehabilit* or benefit*)) or ((disabilit* or sick*) adj3 (leave or absent*)) or retirement or retired or retiring or employ* or unemploy* or job or jobs or occupation* or vocation* or career* or workplace* or workforce* or absenteeism or presenteeism).tw. | 450671 |
| 6 | 4 or 5 | 471208 |
| 7 | 3 and 6 | 944 |
| 8 | limit 7 to yr="2000 - 2021" | 840 |

**AMED (Allied and Complementary Medicine) <1985 to September 2021>**

| 1 | exp Muscular Dystrophy/ or Hemophilia/ or Cystic Fibrosis/ or exp Neural Tube Defects/ or Foot Deformities Congenital/ or exp "Neuropathies Hereditary Motor and Sensory"/ or "Neuropathies Hereditary Sensory and Autonomic"/ | 1373 |
| --- | --- | --- |
| 2 | ((rare adj3 (disease* or disorder*)) or (orphan adj (disease* or disorder*)) or (osteogenesis adj imperfecta) or brittle bone disease* or fragilitas ossium or osteopsathyrosis or ((lobstein* or bruck*) adj (disease* or syndrome*)) or ((skeletal or fibrous) adj3 (dysplasia* or bone*)) or achondroplas* or Diaphyseal Aclasis or ((Hereditary or Familial or Multiple) adj2 (Exostos* or Chondrodysplas* or Osteochondroma*)) or Bessel-Hagen Disease or (Fibro* adj Dysplasia*) or Jaffe Lichtenstein* or (Marfan* adj2 (syndrome* or disease* or disorder* or abiotrophy)) or (Ehlers adj Danlos) or (Loeys adj Dietz) or (genetic adj3 aortic) or ((musc* or limb-girdle) adj dystroph*) or (Glycogen Storage Disease Type adj (II or "2")) or (glycogenos* adj2 (II or "2" or generali*)) or (Pompe adj (disease* or syndrome* or disorder*)) or porphyria* or (porphyrin adj (disorder* or disease* or syndrome*)) or hemophilia* or haemophilia* or ((cystic or pancreatic) adj2 fibros*) or mucoviscidosis or mucoviscoidosis or (pancrea* adj (fibrocystic or fibros* or cystic)) or myelomeningocele* or (neural tube adj2 defect*) or spina bifida* or (congenital adj3 (limb* or extremit*)) or ((limb* or extremit*) adj3 (deformi* or malform* or anomalit*)) or Arthrogryposis multiplex congenita or Amyoplasia* or (Charcot adj Marie) or (Roussy adj Levy) or Peroneal Muscular Atroph* or Hereditary Areflexic Dystasia* or ("hereditary motor and sensory" adj neuropat*) or (HMSN adj2 (I or II or 1A or 1B or "5" or V)) or HMSN1A or HMSN1B or ((Strumpel* or Struempel*) adj1 (disease or syndrome)) or (familial spastic adj (paralysis or paraparesis or paraplegia)) or (dejerine sottas adj (syndrome or disease)) or (myoton* adj2 (dystroph* or atrophica* or myopathy*)) or (Steinert* adj disease*) or Ricker Syndrome* or PROMM* or (Hereditary adj3 Spastic Paraplegia*) or Spastic Paraplegia Hypertrophic Motor Sensory Neuropathy or "CMT with Pyramidal Features" or (Spastic Paraplegia adj2 ("2" or II)) or SPG2 or (rare adj3 hereditary ataxia*) or ((DiGeorge or Di George) adj (syndrome* or sequence or anomaly)) or ((velocardiofacial or Velo Cardio Facial or 22q11 or vcf or pharyngeal pouch or Thymic Aplasia or Sedlackova or Shprintzen) adj2 syndrome*) or "Autosomal Dominant Opitz G Bbb Syndrome" or "Conotruncal Anomaly Face Syndrome" or Catch22 or neurofibromatos* or recklinghausen* or (multiple adj1 neurofibroma*) or ((Turner* or Ullrich* or XO or 45X) adj3 (syndrome* or disease* or state or status or stigma*)) or (Gonadal Dysgenesis adj XO) or Monosomy X or (Bonnevie Ullrich adj (syndrome* or status)) or (mitochondrial adj (disease* or disorder*)) or respiratory chain deficienc* or oxidative phosphorylation deficienc* or (Noonan* adj3 (syndrome* or disease*)) or ((Klinefelter* or XXY or XXYY or XXXXY) adj3 (syndrome* or trisom* or disease*))).mp. | 2348 |
| 3 | 1 or 2 | 2483 |
| 4 | exp Rehabilitation Vocational/ or exp Employment/ or Career Mobility/ or Career Choice/ or Job Satisfaction/ or Sick Leave/ or Absenteeism/ or Retirement/ | 8054 |
| 5 | ((work* adj3 (participat* or disabilit* or abilit* or rehabilit* or challenge* or adapt* or facilitat* or tenure or load or activ* or left or leave or leaving or sustain* or capacit* or measure or measures or protect* or inhibit* or incapacity* or qualit* or life)) or (disabilit* adj3 (pension* or rehabilit* or benefit*)) or ((disabilit* or sick*) adj3 (leave or absent*)) or retirement or retired or retiring or employ* or unemploy* or job or jobs or occupation* or vocation* or career* or workplace* or workforce* or absenteeism or presenteeism).mp. | 34113 |
| 6 | 4 or 5 | 34113 |
| 7 | 3 and 6 | 137 |
| 8 | limit 7 to yr="2000 - 2021" | 102 |

**Cochrane Database of Systematic Reviews**

**Cochrane Central Register of Controlled Trials**

| #1 | ([mh ^"Rare Diseases"] OR [mh ^"Osteogenesis Imperfecta"] OR [mh ^"Exostoses, Multiple Hereditary"] OR [mh "Fibrous Dysplasia of Bone"] OR [mh ^Achondroplasia] OR [mh ^"Marfan Syndrome"] OR [mh ^"Ehlers-Danlos Syndrome"] OR [mh ^"Loeys-Dietz Syndrome"] OR [mh "Muscular Dystrophies"] OR [mh ^"Glycogen Storage Disease Type II"] OR [mh Porphyrias] OR [mh ^"Hemophilia A"] OR [mh ^"Hemophilia B"] OR [mh ^"Cystic Fibrosis"] OR [mh "Neural Tube Defects"] OR [mh "Limb Deformities, Congenital"] OR [mh ^"Charcot-Marie-Tooth Disease"] OR [mh ^"Spastic Paraplegia, Hereditary"] OR [mh ^"DiGeorge Syndrome"] OR [mh Neurofibromatoses] OR [mh ^"Turner Syndrome"] OR [mh "Mitochondrial Diseases"] OR [mh ^"Noonan Syndrome"] OR [mh ^"Klinefelter Syndrome"]) | 4008 |
| --- | --- | --- |
| #2 | (((rare NEAR/2 (disease* OR disorder*)) OR (orphan NEXT (disease* OR disorder*)) OR (osteogenesis NEXT imperfecta) OR (brittle NEXT bone NEXT disease*) OR "fragilitas ossium" OR osteopsathyrosis OR ((lobstein* OR bruck*) NEXT (disease* OR syndrome*)) OR ((skeletal OR fibrous) NEAR/2 (dysplasia* OR bone*)) OR achondroplas* OR "Diaphyseal Aclasis" OR ((Hereditary OR Familial OR Multiple) NEAR/1 (Exostos* OR Chondrodysplas* OR Osteochondroma*)) OR "Bessel-Hagen Disease" OR (Fibro* NEXT Dysplasia*) OR (Jaffe NEXT Lichtenstein*) OR (Marfan* NEAR/1 (syndrome* OR disease* OR disorder* OR abiotrophy)) OR (Ehlers NEXT Danlos) OR (Loeys NEXT Dietz) OR (genetic NEAR/2 aortic) OR ((musc* OR limb-girdle) NEXT dystroph*) OR (("Glycogen Storage Disease Type") NEXT (II OR 2)) OR (glycogenos* NEAR/1 (II OR 2 OR generali*)) OR (Pompe NEXT (disease* OR syndrome* OR disorder*)) OR porphyria* OR (porphyrin NEXT (disorder* OR disease* OR syndrome*)) OR hemophilia* OR haemophilia* OR ((cystic OR pancreatic) NEAR/1 fibros*) OR mucoviscidosis OR mucoviscoidosis OR (pancrea* NEXT (fibrocystic OR fibros* OR cystic)) OR myelomeningocele* OR ("neural tube" NEAR/1 defect*) OR (spina NEXT bifida*) OR (congenital NEAR/2 (limb* OR extremit*)) OR ((limb* OR extremit*) NEAR/2 (deformi* OR malform* OR anomalit*)) OR "Arthrogryposis multiplex congenita" OR Amyoplasia* OR (Charcot NEXT Marie) OR (Roussy NEXT Levy) OR (Peroneal NEXT Muscular NEXT Atroph*) OR (Hereditary NEXT Areflexic NEXT Dystasia*) OR ("hereditary motor and sensory" NEXT neuropat*) OR (HMSN NEAR/1 (I OR II OR "1A" OR "1B" OR "5" OR V)) OR HMSN1A OR HMSN1B OR ((Strumpel* OR Struempel*) NEAR/1 (disease OR syndrome)) OR ("familial spastic" NEXT (paralysis OR paraparesis OR paraplegia)) OR ("dejerine sottas" NEXT (syndrome OR disease)) OR (myoton* NEAR/1 (dystroph* OR atrophica* OR myopathy*)) OR (Steinert* NEXT disease*) OR (Ricker NEXT (Syndrome* OR PROMM*)) OR (Hereditary NEAR/2 "Spastic Paraplegia*") OR "Spastic Paraplegia Hypertrophic Motor Sensory Neuropathy" OR "CMT with Pyramidal Features" OR ("Spastic Paraplegia" NEAR/1 ("2" OR II)) OR SPG2 OR (rare NEAR/2 (hereditary NEXT ataxia*)) OR ((DiGeorge OR "Di George") NEXT (syndrome* OR sequence OR anomaly)) OR ((velocardiofacial OR "Velo Cardio Facial" OR "22q11" OR vcf OR "pharyngeal pouch" OR "Thymic Aplasia" OR Sedlackova OR Shprintzen) NEAR/1 syndrome*) OR "Autosomal Dominant Opitz G Bbb Syndrome" OR "Conotruncal Anomaly Face Syndrome" OR "Catch22" OR neurofibromatos* OR recklinghausen* OR (multiple NEAR/1 neurofibroma*) OR ((Turner* OR Ullrich* OR XO OR "45X") NEAR/2 (syndrome* OR disease* OR state OR status OR stigma*)) OR ("Gonadal Dysgenesis" NEXT XO) OR "Monosomy X" OR ("Bonnevie Ullrich" NEXT (syndrome* OR status)) OR (mitochondrial NEXT (disease* OR disorder*)) OR (respiratory NEXT chain NEXT deficienc*) OR (oxidative NEXT phosphorylation NEXT deficienc*) OR (Noonan* NEAR/2 (syndrome* OR disease*)) OR ((Klinefelter* OR XXY OR XXYY OR XXXXY) NEAR/2 (syndrome* OR trisom* OR disease*))):ti,ab,kw | 108771 |
| #3 | #1 OR #2 | 109032 |
| #4 | ([mh Work] OR [mh ^Employment] OR [mh "Rehabilitation, Vocational"] OR [mh ^Workplace] OR [mh Occupations] OR [mh ^"Occupational Stress"] OR [mh ^"Job Satisfaction"] OR [mh ^"Work-Life Balance"] OR [mh ^"Sick Leave"] OR [mh ^Absenteeism] OR [mh ^Retirement] OR [mh ^"Vocational Guidance"] OR [mh ^"Career Choice"] OR [mh ^"Work Capacity Evaluation"] OR [mh ^Presenteeism]) | 4270 |
| #5 | ((work* NEAR/2 (participat* OR disabilit* OR abilit* OR rehabilit* OR challenge* OR adapt* OR facilitat* OR tenure OR load OR activ* OR left OR leave OR leaving OR sustain* OR capacit* OR measure OR measures OR protect* OR inhibit* OR incapacity* OR qualit* OR life)) OR (disabilit* NEAR/2 (pension* OR rehabilit* OR benefit*)) OR ((disabilit* OR sick*) NEAR/2 (leave OR absent*)) OR retirement OR retired OR retiring OR *employ* OR job OR jobs OR occupation* OR vocation* OR career* OR workplace* OR workforce* OR absenteeism OR presenteeism):ti,ab,kw | 52413 |
| #6 | #4 OR #5 | 52933 |
| #7 | #3 AND #6 | 4883 |
| #8 | #7 in Cochrane Reviews, Cochrane Protocols with Cochrane Library publication date from Jan 2000 to Dec 2021 | 111 |
| #9 | #7 in Trials with Publication Year from 2000 to 2021 | 4765 |

**CINAHL (EBSCO)**

| S1 | (MH "Rare Diseases" OR MH "Osteogenesis Imperfecta" OR MH "Fibrous Dysplasia of Bone+" OR MH "Achondroplasia" OR MH "Marfan Syndrome" OR MH "Ehlers-Danlos Syndrome" OR MH "Loeys-Dietz Syndrome" OR MH "Muscular Dystrophy+" OR MH "Glycogen Storage Disease" OR MH "Porphyrias+" OR MH "Hemophilia+" OR MH "Cystic Fibrosis" OR MH "Neural Tube Defects+" OR MH "Limb Deformities, Congenital+" OR MH "Neuropathies, Hereditary Motor and Sensory+" OR MH "DiGeorge Syndrome" OR MH "Neurofibromatoses+" OR MH "Turner's Syndrome" OR MH "Mitochondrial Diseases+" OR MH "Noonan Syndrome" OR MH "Klinefelter's Syndrome") | 34,453 |
| --- | --- | --- |
| S2 | ((rare N1 (disease* OR disorder*)) OR (orphan N0 (disease* OR disorder*)) OR "osteogenesis imperfecta" OR "brittle bone disease*" OR "fragilitas ossium" OR osteopsathyrosis OR ((lobstein* OR bruck*) N0 (disease* OR syndrome*)) OR ((skeletal OR fibrous) N1 (dysplasia* OR bone*)) OR achondroplas* OR "Diaphyseal Aclasis" OR ((Hereditary OR Familial OR Multiple) N1 (Exostos* OR Chondrodysplas* OR Osteochondroma*)) OR "Bessel-Hagen Disease" OR ("Fibro* N0 Dysplasia*) OR "Jaffe Lichtenstein*" OR (Marfan* N1 (syndrome* OR disease* OR disorder* OR abiotrophy)) OR "Ehlers Danlos" OR "Loeys Dietz" OR (genetic N1 aortic) OR ((musc* OR limb-girdle) N0 dystroph*) OR (("Glycogen Storage Disease Type") N0 (II OR 2)) OR (glycogenos* N1 (II OR 2 OR generali*)) OR (Pompe N0 (disease* OR syndrome* OR disorder*)) OR porphyria* OR (porphyrin N0 (disorder* OR disease* OR syndrome*)) OR hemophilia* OR haemophilia* OR ((cystic OR pancreatic) N1 fibros*) OR mucoviscidosis OR mucoviscoidosis OR (pancrea* N0 (fibrocystic OR fibros* OR cystic)) OR myelomeningocele* OR ("neural tube" N1 defect*) OR "spina bifida*" OR (congenital N1 (limb* OR extremit*)) OR ((limb* OR extremit*) N1 (deformi* OR malform* OR anomalit*)) OR "Arthrogryposis multiplex congenita" OR Amyoplasia* OR "Charcot Marie" OR "Roussy Levy" OR "Peroneal Muscular Atroph*" OR "Hereditary Areflexic Dystasia*" OR ("hereditary motor and sensory" N0 neuropat*) OR (HMSN N1 (I OR II OR "1A" OR "1B" OR "5" OR V)) OR HMSN1A OR HMSN1B OR ((Strumpel* OR Struempel*) N1 (disease OR syndrome)) OR ("familial spastic" N0 (paralysis OR paraparesis OR paraplegia)) OR ("dejerine sottas" N0 (syndrome OR disease)) OR (myoton* N1 (dystroph* OR atrophica* OR myopathy*)) OR "Steinert* disease*" OR "Ricker Syndrome*" OR PROMM* OR (Hereditary N1 "Spastic Paraplegia*") OR "Spastic Paraplegia Hypertrophic Motor Sensory Neuropathy" OR "CMT with Pyramidal Features" OR ("Spastic Paraplegia" N1 ("2" OR II)) OR SPG2 OR (rare N1 "hereditary ataxia*") OR ((DiGeorge OR "Di George") N0 (syndrome* OR sequence OR anomaly)) OR ((velocardiofacial OR "Velo Cardio Facial" OR "22q11" OR vcf OR "pharyngeal pouch" OR "Thymic Aplasia" OR Sedlackova OR Shprintzen) N1 syndrome*) OR "Autosomal Dominant Opitz G Bbb Syndrome" OR "Conotruncal Anomaly Face Syndrome" OR Catch22 OR neurofibromatos* OR recklinghausen* OR (multiple N1 neurofibroma*) OR ((Turner* OR Ullrich* OR XO OR S45 X) N1 (syndrome* OR disease* OR state OR status OR stigma*)) OR ("Gonadal Dysgenesis" N0 XO) OR "Monosomy X" OR ("Bonnevie Ullrich" N0 (syndrome* OR status)) OR (mitochondrial N0 (disease* OR disorder*)) OR "respiratory chain deficienc*" OR "oxidative phosphorylation deficienc*" OR (Noonan* N1 (syndrome* OR disease*)) OR ((Klinefelter* OR XXY OR XXYY OR XXXXY) N1 (syndrome* OR trisom* OR disease*))) | 16,250 |
| S3 | S1 OR S2 | 46,186 |
| S4 | (MH "Work+" MH "Employment+" OR MH "Rehabilitation, Vocational" OR MH "Work Environment+" OR MH "Occupations and Professions+" OR MH "Stress, Occupational" OR MH "Job Satisfaction" OR MH "Work-Life Balance" OR MH "Sick Leave" OR MH "Absenteeism" OR MH "Presenteeism" OR MH "Vocational Guidance" OR MH "Retirement" OR MH "Career Planning and Development" OR MH "Work Capacity Evaluation") | 187,270 |
| S5 | ((work* N1 (participat* OR disabilit* OR abilit* OR rehabilit* OR challenge* OR adapt* OR facilitat* OR tenure OR load OR activ* OR left OR leave OR leaving OR sustain* OR capacit* OR measure OR measures OR protect* OR inhibit* OR incapacity* OR qualit* OR life)) OR (disabilit* N1 (pension* OR rehabilit* OR benefit*)) OR ((disabilit* OR sick*) N1 (leave OR absent*)) OR retirement OR retired OR retiring OR employ* OR unemploy* OR job OR jobs OR occupation* OR vocation* OR career* OR workplace* OR workforce* OR absenteeism OR presenteeism) | 534,490 |
| S6 | S4 OR S5 | 590,939 |
| S7 | S3 AND S6 | 1,104 |
| S8 | S3 AND S6 Limiters - Published Date: 20000101-20211231; Exclude MEDLINE records | 474 |

**Scopus**

TITLE-ABS-KEY ((((rare W/2 (disease* OR disorder*)) OR (orphan W/0 (disease* OR disorder*)) OR ("osteogenesis imperfecta") OR "brittle bone disease*" OR "fragilitas ossium" OR osteopsathyrosis OR ((lobstein* OR bruck*) W/0 (disease* OR syndrome*)) OR ((skeletal OR fibrous) W/2 (dysplasia* OR bone*)) OR achondroplas* OR "Diaphyseal Aclasis" OR ((hereditary OR familial OR multiple) W/1 (exostos* OR chondrodysplas* OR osteochondroma*)) OR "Bessel-Hagen Disease" OR (fibro* W/0 dysplasia*) OR "Jaffe Lichtenstein*" OR (marfan* W/1 (syndrome* OR disease* OR disorder* OR abiotrophy)) OR "Ehlers Danlos" OR "Loeys Dietz" OR (genetic W/2 aortic) OR ((musc* OR limb-girdle) W/0 dystroph*) OR ("Glycogen Storage Disease Type" W/0 (ii OR "2")) OR (glycogenos* W/1 (ii OR "2" OR generali*)) OR (pompe W/0 (disease* OR syndrome* OR disorder*)) OR porphyria* OR (porphyrin W/0 (disorder* OR disease* OR syndrome*)) OR hemophilia* OR haemophilia* OR ((cystic OR pancreatic) W/1 fibros*) OR mucoviscidosis OR mucoviscoidosis OR (pancrea* W/0 (fibrocystic OR fibros* OR cystic)) OR myelomeningocele* OR (neural AND tube W/1 defect*) OR "spina bifida*" OR (congenital W/2 (limb* OR extremit*)) OR ((limb* OR extremit*) W/2 (deformi* OR malform* OR anomalit*)) OR "Arthrogryposis multiplex congenita OR Amyoplasia*" OR "Charcot Marie" OR "Roussy Levy" OR "Peroneal Muscular Atroph*" OR "Hereditary Areflexic Dystasia*" OR ("hereditary motor and sensory" W/0 neuropat*) OR (hmsn W/1 (i OR ii OR 1a OR 1b OR 5 OR v)) OR hmsn1a OR hmsn1b OR ((strumpel* OR struempel*) W/0 (disease OR syndrome)) OR (familial AND spastic W/0 (paralysis OR paraparesis OR paraplegia)) OR ("dejerine sottas" W/0 (syndrome OR disease)) OR (myoton* W/1 (dystroph* OR atrophica* OR myopathy*)) OR (steinert* W/0 disease*) OR "Ricker Syndrome*" OR promm* OR (hereditary W/2 "Spastic Paraplegia*") OR "Spastic Paraplegia Hypertrophic Motor Sensory Neuropathy" OR "CMT with Pyramidal Features" OR ("Spastic Paraplegia" W/1 (2 OR ii)) OR spg2 OR (rare W/2 "hereditary ataxia*") OR ((digeorge OR "Di George") W/0 (syndrome* OR sequence OR anomaly)) OR ((velocardiofacial OR "Velo Cardio Facial" OR 22q11 OR vcf OR "pharyngeal pouch" OR "Thymic Aplasia" OR sedlackova OR shprintzen) W/1 syndrome*) OR "Autosomal Dominant Opitz G Bbb Syndrome" OR "Conotruncal Anomaly Face Syndrome" OR catch22 OR neurofibromatos* OR recklinghausen* OR (multiple W/0 neurofibroma*) OR ((turner* OR ullrich* OR xo OR 45x) W/2 (syndrome* OR disease* OR state OR status OR stigma*)) OR ("Gonadal Dysgenesis" W/0 xo) OR "Monosomy X" OR ("Bonnevie Ullrich" W/0 (syndrome* OR status)) OR (mitochondrial W/0 (disease* OR disorder*)) OR "respiratory chain deficienc*" OR "oxidative phosphorylation deficienc*" OR (noonan* W/2 (syndrome* OR disease*)) OR ((klinefelter* OR xxy OR xxyy OR xxxxy) W/2 (syndrome* OR trisom* OR disease*))) AND ((work* W/2 (participat* OR disabilit* OR abilit* OR rehabilit* OR challenge* OR adapt* OR facilitat* OR tenure OR load OR activ* OR left OR leave OR leaving OR sustain* OR capacit* OR measure OR measures OR protect* OR inhibit* OR incapacity* OR qualit* OR life)) OR (disabilit* W/2 (pension* OR rehabilit* OR benefit*)) OR ((disabilit* OR sick*) W/2 (leave OR absent*)) OR retirement OR retired OR retiring OR employ* OR unemploy* OR job OR jobs OR occupation* OR vocation* OR career* OR workplace* OR workforce* OR absenteeism OR presenteeism))) AND (LIMIT-TO (PUBYEAR, 2021) OR LIMIT-TO (PUBYEAR, 2020) OR LIMIT-TO (PUBYEAR, 2019) OR LIMIT-TO (PUBYEAR, 2018) OR LIMIT-TO (PUBYEAR, 2017) OR LIMIT-TO (PUBYEAR, 2016) OR LIMIT-TO (PUBYEAR, 2015) OR LIMIT-TO (PUBYEAR, 2014) OR LIMIT-TO (PUBYEAR, 2013) OR LIMIT-TO (PUBYEAR, 2012) OR LIMIT-TO (PUBYEAR, 2011) OR LIMIT-TO (PUBYEAR, 2010) OR LIMIT-TO (PUBYEAR, 2009) OR LIMIT-TO (PUBYEAR, 2008) OR LIMIT-TO (PUBYEAR, 2007) OR LIMIT-TO (PUBYEAR, 2006) OR LIMIT-TO (PUBYEAR, 2005) OR LIMIT-TO (PUBYEAR, 2004) OR LIMIT-TO (PUBYEAR, 2003) OR LIMIT-TO (PUBYEAR, 2002) OR LIMIT-TO (PUBYEAR, 2001) OR LIMIT-TO (PUBYEAR, 2000))

**Science Citation Index Expanded, Social Sciences Citation Index, Arts & Humanities Citation Index, Conference Proceedings Citation Index- Science, Conference Proceedings Citation Index Social Science & Humanities, Emerging (Web of Science, Clarivate)**

TS=(((((rare NEAR/2 (disease* OR disorder*)) OR (orphan NEAR/0 (disease* OR disorder*)) OR ("osteogenesis imperfecta") OR "brittle bone disease*" OR "fragilitas ossium" OR osteopsathyrosis OR ((lobstein* OR bruck*) NEAR/0 (disease* OR syndrome*)) OR ((skeletal OR fibrous) NEAR/2 (dysplasia* OR bone*)) OR achondroplas* OR "Diaphyseal Aclasis" OR ((Hereditary OR Familial OR Multiple) NEAR/1 (Exostos* OR Chondrodysplas* OR Osteochondroma*)) OR "Bessel-Hagen Disease" OR (Fibro* NEAR/0 Dysplasia*) OR "Jaffe Lichtenstein*" OR (Marfan* NEAR/1 (syndrome* OR disease* OR disorder* OR abiotrophy)) OR "Ehlers Danlos" OR "Loeys Dietz" OR (genetic NEAR/2 aortic) OR ((musc* OR limb-girdle) NEAR/0 dystroph*) OR ("Glycogen Storage Disease Type" NEAR/0 (II OR "2")) OR (glycogenos* NEAR/1 (II OR "2" OR generali*)) OR (Pompe NEAR/0 (disease* OR syndrome* OR disorder*)) OR porphyria* OR (porphyrin NEAR/0 (disorder* OR disease* OR syndrome*)) OR hemophilia* OR haemophilia* OR ((cystic OR pancreatic) NEAR/1 fibros*) OR mucoviscidosis OR mucoviscoidosis OR (pancrea* NEAR/0 (fibrocystic OR fibros* OR cystic)) OR myelomeningocele* OR (neural tube NEAR/1 defect*) OR "spina bifida*" OR (congenital NEAR/2 (limb* OR extremit*)) OR ((limb* OR extremit*) NEAR/2 (deformi* OR malform* OR anomalit*)) OR "Arthrogryposis multiplex congenita OR Amyoplasia*" OR "Charcot Marie" OR "Roussy Levy" OR "Peroneal Muscular Atroph*" OR "Hereditary Areflexic Dystasia*" OR ("hereditary motor and sensory" NEAR/0 neuropat*) OR (HMSN NEAR/1 (I OR II OR 1A OR 1B OR 5 OR V)) OR HMSN1A OR HMSN1B OR ((Strumpel* OR Struempel*) NEAR/0 (disease OR syndrome)) OR (familial spastic NEAR/0 (paralysis OR paraparesis OR paraplegia)) OR ("dejerine sottas" NEAR/0 (syndrome OR disease)) OR (myoton* NEAR/1 (dystroph* OR atrophica* OR myopathy*)) OR (Steinert* NEAR/0 disease*) OR "Ricker Syndrome*" OR PROMM* OR (Hereditary NEAR/2 "Spastic Paraplegia*") OR "Spastic Paraplegia Hypertrophic Motor Sensory Neuropathy" OR "CMT with Pyramidal Features" OR ("Spastic Paraplegia" NEAR/1 (2 OR II)) OR SPG2 OR (rare NEAR/2 "hereditary ataxia*") OR ((DiGeorge OR "Di George") NEAR/0 (syndrome* OR sequence OR anomaly)) OR ((velocardiofacial OR "Velo Cardio Facial" OR 22q11 OR vcf OR "pharyngeal pouch" OR "Thymic Aplasia" OR Sedlackova OR Shprintzen) NEAR/1 syndrome*) OR "Autosomal Dominant Opitz G Bbb Syndrome" OR "Conotruncal Anomaly Face Syndrome" OR Catch22 OR neurofibromatos* OR recklinghausen* OR (multiple NEAR/0 neurofibroma*) OR ((Turner* OR Ullrich* OR XO OR 45X) NEAR/2 (syndrome* OR disease* OR state OR status OR stigma*)) OR ("Gonadal Dysgenesis" NEAR/0 XO) OR "Monosomy X" OR ("Bonnevie Ullrich" NEAR/0 (syndrome* OR status)) OR (mitochondrial NEAR/0 (disease* OR disorder*)) OR "respiratory chain deficienc*" OR "oxidative phosphorylation deficienc*" OR (Noonan* NEAR/2 (syndrome* OR disease*)) OR ((Klinefelter* OR XXY OR XXYY OR XXXXY) NEAR/2 (syndrome* OR trisom* OR disease*))) AND ((work* NEAR/2 (participat* OR disabilit* OR abilit* OR rehabilit* OR challenge* OR adapt* OR facilitat* OR tenure OR load OR activ* OR left OR leave OR leaving OR sustain* OR capacit* OR measure OR measures OR protect* OR inhibit* OR incapacity* OR qualit* OR life)) OR (disabilit* NEAR/2 (pension* OR rehabilit* OR benefit*)) OR ((disabilit* OR sick*) NEAR/2 (leave OR absent*)) OR retirement OR retired OR retiring OR employ* OR unemploy* OR job OR jobs OR occupation* OR vocation* OR career* OR workplace* OR workforce* OR absenteeism OR presenteeism)))) and 2021 or 2020 or 2019 or 2018 or 2017 or 2016 or 2015 or 2014 or 2013 or 2012 or 2011 or 2010 or 2009 or 2008 or 2007 or 2006 or 2005 or 2004 or 2003 or 2002 or 2001 or 2000 (Publication Years)

**SveMed+**

| 1 | (noexp:"Rare Diseases" OR noexp:"Osteogenesis Imperfecta" OR noexp:"Exostoses, Multiple Hereditary" OR exp:"Fibrous Dysplasia of Bone" OR noexp:"Achondroplasia" OR noexp:"Marfan Syndrome" OR noexp:"Ehlers-Danlos Syndrome" OR noexp:"Loeys-Dietz Syndrome" OR exp:"Muscular Dystrophies" OR noexp:"Glycogen Storage Disease Type II" OR exp:"Porphyrias" OR noexp:"Hemophilia A" OR noexp:"Hemophilia B" OR noexp:"Cystic Fibrosis" OR exp:"Neural Tube Defects" OR exp:"Limb Deformities, Congenital" OR noexp:"Charcot-Marie-Tooth Disease" OR noexp:"Spastic Paraplegia, Hereditary" OR noexp:"DiGeorge Syndrome" OR exp:"Neurofibromatoses" OR noexp:"Turner Syndrome" OR exp:"Mitochondrial Diseases" OR noexp:"Noonan Syndrome" OR noexp:"Klinefelter Syndrome") | 879 |
| --- | --- | --- |
| 2 | (exp:"Work" OR noexp:"Employment" OR exp:"Rehabilitation, Vocational" OR noexp:"Workplace" OR exp:"Occupations" OR noexp:"Occupational Stress" OR noexp:"Job Satisfaction" OR noexp:"Work-Life Balance" OR noexp:"Sick Leave" OR noexp:"Absenteeism" OR noexp:"Presenteeism" OR noexp:"Retirement" OR noexp:"Vocational Guidance" OR noexp:"Career Choice" OR noexp:"Work Capacity Evaluation") | 4454 |
| 3 | #1 AND #2 | 8 |
