# Supplementary material for: Work participation in adults with rare genetic diseases - a scoping review
Source: BMC Public Health. 2023 May 19;23:910. doi: 10.1186/s12889-023-15654-3 (PMC10197424; doi:10.1186/s12889-023-15654-3)
Supplement: Supplementary file 4 — Supplementary table 4: Data extraction of included articles on work participation in rare genetic diseases [file 12889_2023_15654_MOESM4_ESM.docx]

| **Supplementary table 4: Data extraction of included articles on work participation in rare genetic diseases** | | | | | |
| --- | --- | --- | --- | --- | --- |
| **Reference:**   - First Author - Year of publication - Title of the article - Title journal, - Country/Countries | **Aim of the study**   - As reported by the authors | **Participants**   - N =number of patients - G=Gender - A=Age - D=Diagnosis - R=Recruitement context | **Study design and focus on work participation (WP)**   - Qantitative cross-sectional, qualitative, mixed method, internvention or validation of outcome measures study? - WP primary or secondary aim/ outcome in the study? | **Methods and outcome measures**   - Use of study-specific questionnaire? - Use of a standardized instruments (Which instrument?) - Individual interviews -Focus group interviews - Combining methodsQuestions (measurement) on WP | **Short description of results related to WP related research questions:**   - Prevalence of work participation/work disability, associated factors to WP and/or other aspects (experiences, perception, intervention effects etc) |
| **Rare inborn errors of metabolism diseases (ORPHA: 68367)** | | | | | |
| ***Fabry disease (FS) (ORPHA:324)*** | | | | | |
| 125  Cole et al 2007. Depression in adults with Fabry disease: A common and under‐diagnosed problem.  Journal of Inherited Metabolic Disease  United Kingdom | To determine the prevalence of depression in adult patients with AFD in the United Kingdom and to identify factors associated with depression in this patient group. | N = 184  G = 69% female  A =18-76 y, 44y mean  D = Fabry syndrome  R = Four designated lysosomal storage disorder centres | - Cross-sectional quantitative study (postal). - Investigating WP was one secondary outcome | - Study specific self-administered questionnaires on sociodemographic and clinical factors included: The Centre for Epidemiological studies Depression Scale (CES-D). - Questions on WP: not described. | - 59% employed/seeking work, 13 %, retired, 16% sick leave, 3% students, 8% homeworking. - Unemployment and lower income significantly associated with depression. |
| ***Gaucher disease type 1 (GD1) (ORPHA:77259)*** | | | | | |
| 126  Dinur et al 2020. Patient reported outcome measures in a large cohort of patients with type 1 Gaucher disease.  Orphanet Journal of Rare Diseases.  Israel | To report of patient-reported outcome measures (PROM) in a large cohort of patients with type 1 Gaucher disease (GD1) enabling us to study predictors of the reported outcomes. | N=192  G=57,8% female  A=19-91y  D=Gaucher disease.  R=Gaucher Unit at Shaare Zedek Medical Center. | - Cross-sectional quantitative study (by mobile phone). - Investigating WP was one secondary outcome. | - Study specific questionnaire on sociodemographic, clinical and treatment factors and Gaucher Disease-PROM. Clinical data from clinical charts. - Questions about WP: My disease has resticed my education/job (yes/no). | - Almost 40 % reported that the diseases had   restricted education and/or job. Untreated patients reported that disease did not restrict their education/job. |
| ***Glycogen storage disease type 1 (ORPHA:79201)*** | | | | | |
| 127  Garbade et al 2021.  Impact of glycogen storage disease type I on adult daily life: a survey.  Orphanet Journal of Rare Diseases.  Germany | To assess the impact of glycogen storage disease on adult daily life. | N=34  G=39,4 % female  A=17-54 y, 26y mean  D=Glycogen storage disease type I. GSD Ia (n=27) and GSD Ib (n=7)  R=German metabolic centres | - Cross- sectional quantitative study (muticentre) - Investigating WP was one secondary outcome. | - Study specific questionnaire on sociodemographic aspects, social life and patients’ attitude towards the diseases. - Questions on WP: Not described. | - 52% working, 26% students/school, 12% apprenticeship, 9% were out of work. - Among the working individuals, four patients (16%) reported to work mainly physically, while 68% predominantly performed office work. 76% communicated openly about their disease to superios at work. |
| ***Pompe disease (ORPHA:420429)*** | | | | | |
| 128  Chen et al 2021. Quality of life and its contributors among adults with late-onset Pompe disease in China.  Orphanet Journal of Rare Disease.  China | To assess the QOL of adult patients with Pompe in China, and to explore the social and economic factors that may contribute to QoL. | N=68 (other rare diseases n=1067)  G=51,4% female  A=30/35,71y mean  D=Pompe disease  R=Rare Diseases patient organization and China Pompe care centres | - Cross-sectional quantitative study (primarily online). - Investigating WP was one secondary outcome. | - Study specific questionnaire on sociodemographic aspects, including: The World Health Organization Quality of Life: Brief Version (WHOQOL-BREF). - Questions on WP: Not described. | - 59% of people with Pome disease were working compared to 52.67% of people with other rare diseases. - WP significantly associated with psychological environment, sociale relationship and QoL. Pompe disease was associated with less WP, compared to other rare diseases - WP was found to play an important role in QoL |
| 49  Hagemans et al 2007. Impact of late-onset Pompe disease on participation in daily life activities: Evaluation of the Rotterdam Handicap Scale.  Neuromuscular Disorders.  Multi-country: Australia, Canada, Germany, France, the Netherlands, the United Kingdom and the United States | To measure the impact of late-onset Pompe disease on participation in daily life activities. | N= 257  G=53% female  A= 19-79, 48y mean  D=Pompe disease  R=Patient organizations affiliated with the International Pompe Association | - Cross-sectional quantitative study. - Investigating WP was one secondary outcome. | - Study specific questionnaire,   on medical history, current disease status and use of care, including: The Short-form Health Survey for Medical Outcomes Study (SF-36).   - Questions on WP: The Rotterdam Handicap Scale (RHS) included questions about work related aspects. | - 40 % indicated that they were not able to fulfil their prior job/stud and return to fomer job and 9 % only partly. Pompe disease has large negative impact on WP, and the ability to fulfil the work. |
| 96  Kanters et al 2011. Burden of illness of Pompe disease in patients only receiving supportive care. Journal of Inherited Metabolic Disease. Netherland | To assess the burden of Pompe disease with respect to all aspects: societal costs, the use of home care and informal care, productivity losses, and losses in HRQoL. | N=80  G=62,5% female  A=51y mean  D=Pompe disease  R=Clinic The Center for Lysosomal and Metabolic Diseases at Erasmus Medical Center | - Prospective study - Investigating WP was one secondary outcome.   . | - A study specific questionnaire about sociodemographic, health and economic aspects, including: The EuroQoL 5L(EQ-5D). - Questions about work: Productivity losses due to absence from work were included in the questionnaire. | - 39% were employed, 52 % of whom indicated they were working fewer hours than they would without the disease (average 14 h fewer). 40% had stopped working due to their disease; another 20% had reduced their working hours. - Reduced working hours and stop working were associated to the disease. - Total productively losses were calculated to accumulate to over 22 445 euro per patient, including estimated productivity losses 2633 pund. |
| ***Porphyria (ORPHA:95157, 79276)*** | | | | | |
| 97  Baravelli et al 2020. Sick leave, disability, and mortality in acute hepatic porphyria: a nationwide cohort study.  Orphanet Journal of Rare Diseases.  Norway | To investigate the risk of long-term sick leave, disability pension, and premature death in individuals with Acute hepatic porphyria (AHP compared to the general population. | N=333  G=69% female  A=29y mean  D=Acute hepatic porphyria (AHP  R=The Norwegian Porphyria Centre (NAPOS) | - Prospective study. - Investigating WP was one secondary outcome. | - Study specific questionnaire of sociodemographic and clinical aspects. - Questions about WP: the Norwegian Labour and Welfare Administration records regarding disbursements of various benefits, including long-term sick leave benefit and disability pension. | - 70 % accessed lomg-term sick leave thougout the study period. - The diagnosis was associated with increased risk of accessing long-term sick leave and disability pension, compared to the general population. - The median age when accessing disability pension was 45 years, 21 years younger than the general population. |
| 129  Hammersland et al 2019. Self-efficacy and self-management strategies in acute intermittent porphyria.  BMC, Health Service Research.  Norway | To describe self-efficacy in self-reported symptomatic acute intermittent porphyria carriers and to determine whether they implemented changes in behavior after receiving the diagnosis. | N=140  G=58% female  A=18-89y, 52y mean  D=Acute intermittent porphyria (AIP)  R=The Norwegian Porphyria Centre | - Cross-sectional quantitative study (postal). - Investigating WP was one secondary outcome. | - Study specific questionnaire with 14 questions about sociodemographic and clinical aspects, including: The General Perceived Self-efficacy Scale (GSES), Satisfaction with Genetic Counseling Scale (SCS). - Questions on WP: Not described. | - 62% employed, 19% pension, 16 % disabled, 3% others. - Significantly lower WP in the symptomatic groups compared to the asymtomatic group. Genetic counseling of patients were associated with higher level of education and employment, compared to those not receiving genetic counseling. |
| ***Familial chylomicronemia syndrome (FCS) (ORPHA:444490)*** | | | | | |
| 50  Davidson et al 2018. The burden of familial chylomicronemia syndrome: Results from the global IN-FOCUS study.  Journal of Clinical Lipidology.  USA (multinational: 10 countries, 62% from USA) | To understand the ways in which familial chylomicronemia syndrome (FCS) impacts patients' lives. | N=166 patients  G=30% female  A=33y mean  D= Familial chylomicronemia syndrome (FCS) is a rare genetic disorder  R=Open web-based | - Cross-sectional quantitative study (online global web-based). - Investigating WP was one secondary outcome. | - Survey study specific questions on sociodemographic aspects, diagnostic experience, symptoms, comorbidities, including: The Short-form Health Survey for Medical Outcomes Study (SF-36), The Pancreatitis Quality of Life Instrument. - Questions on WP: Not described. | - WP 60 % were employed full/part time and 40 unemployed. Of those unemployed or employed part time, 94% felt that their employment status was due to the disease.. - Patients experienced significant clinical and psychosocial burdens that limit employment and social interactions. Of the homemakers, 40 % felt their lack of employment opportunities were due to disease. |
| 130  Gaudet et al 2020. The burden of familial chylomicronemia syndrome in Canadian patients  Health and Disease.  Journal of Clinical Lipidology.  Canada | To understand the burden of illness of familial chylomicronemia syndrome on Canadian patients’ lives. | N=37  G=11% female  A=18-56y, 33y mean  D=Chylomicronemia syndrome  R=Open web-based-via word of mouth, social media etc | - Cross sectional quantitative study (online global web-based). - Investigating WP was one secondary outcome. | - Study specific questionnaire on sociodemographic aspects, diagnostic experience, symptoms, comorbidities, included: The Short-form Health Survey for Medical Outcomes Study (SF-36), The Pancreatitis Quality of Life Instrument. - Questions on WP: Not described. | - 19% full time work, 40% part time work, 8% full/part time student, 27 % unemployed, 3% retired, 3 % home-makers. - The diagnosis was significantly associated to impact (97 %) on career choice and employment status. - 76% choose careers below their level of abilities. |
| **Rare genetic bone diseases (ORPHA:183524)** | | | | | |
| ***Multiple osteochondromas/ multiple hereditary exostoses (ORPHA:321)*** | | | | | |
| 131  Bathen et al 2019. Fatigue and pain in children and adults with multiple osteochondromas in Norway, a cross-sectional study.  [International Journal of Orthopaedic and Trauma Nursing](https://www.sciencedirect.com/science/journal/18781241).  Norway | To investigate prevalence of fatigue and pain in Norwegian children and adults with multiple osteochondromas (MO).  To compare prevalence of fatigue with reported prevalence in other groups and explore some factors that may contribute to fatigue in this population. | N= 21 adults  G=62% female  A=20-37y  D=Multiple osteochondromas  R=TRS National  Resource Centre for Rare Disorders. | - Cross-sectional quantitative study. - Investigating WP was one secondary outcome. | - Study specific questionnaire on medical and sociodemographic questions including: The Fatigue Severity Scale (FSS), The Numeric rating scale measuring pain. - Questions on WP: Not described. | - 38,1 % working full time, 23,8% part time, 4,8% applying jobs, 4,8% studying, 28,6 % disability benefits, 19.0% work rehabilitation benefit. - Ten of 21 adults had challenges with work participation, being on disability benefits. |
| 132  Goud et al 2012. Pain, physical and social functioning, and quality of life in individuals with multiple hereditary exostoses in the Netherlands.  Journal of Bone and Joint Surgery.  Netherlands | To assess pain and quality of life in a large cohort of patients with multiple hereditary exostoses. | N=184 adults (and 99 children)  G=55% female  A=>18y  D=Multiple Hereditary Exostoses.  R=Dutch patient coalition and three national referral centres. | - Cross sectional quantitative study. - Investigating WP was one secondary outcome. | - Study specific questionnaire of sociodemographic aspects family history and medical aspects, including: The Short-form Health Survey for Medical Outcomes Study (SF-36), - Questions on WP: Not described. | - 65 % were working, 35 % not working, of these 20% medically unfit to work. - 21% needed adjustment at the work-place, 28% had changed jobs because of the disease, 56% had problems some times during occupation including workload to heavy, 8% had functional impairments. |
| ***Osteogenesis imperfecta (OI) (ORPHA:666)*** | | | | | |
| 133  Balkefors et al 2012. Functioning and quality of life in adults with mild‐to‐moderate osteogenesis imperfecta.  Physiotheraphy Research. International.  Sweden | To describe physical ability, quality and satisfaction with life, joint mobility and muscle function in adults with mild to moderate osteogenesis imperfecta (OI). | N=29  G=62% female  A=21-71y  D=Mild‐to‐moderate osteogenesis imperfecta  R=Patient Register at OI Centre, Karolinska University Hospital | - Cross-sectional quantitative study and clinical examination. - Investigating WP was one secondary outcome. | - Study specific questionnaire about sociodemographics combined with clinical examinations, including: Disability Rating Scale (DRI), Physical Activity Questionnaire (PAQ), The Short-form Health Survey for Medical Outcomes Study (SF-36), Life Satisfaction Questionnaire 11 (Lisat 11). - Questions about WP: One question from Lisat 11. | - 48% in sedentary work, 27% active load work, 24% student/pensioner/sick leave. - Disability alone did not decrease the individual’s satisfaction with life, |
| 134  Montpetit et al 2011. Activities and participation in young adults with osteogenesis imperfecta. Journal of Pediatric Rehabilitation Medicine.  Canada | To compare the activities and participation in the domains of mobility, self-care, domestic life and social functioning in young adults according to osteogenesis imperfecta (OI) types. | N=24  G=not reported  A=25y mean  D=Osteogenesis imperfecta type 1, III, IV, V  R=Pediatric orthopedic hospital for OI | - Cross-sectional quantitative study (postal). - Investigating WP was one secondary outcome. | - Study specific questionnaire about sociodemographics and clinical aspects with open-ended questions, including: The Functional Independent Measure (FIM), The Instrumental Activities Measures (IAM). - Questions on WP: Not described. | - 62% working, 38% not working. - Significant more people with OI type I were working compared to type II,III and IV - Participants with OI type I reported full independence, and young adults with OI type III had significantly lower activity scores in employment. |
| 135  Wekre et al 2010.  A population-based study of demographical variables and ability to perform activities of daily living in adults with osteogenesis imperfecta.  Journal of Disability and Rehabilitation.  Norway | To study demographical variables in an adult with osteogenesis imperfecta (OI) compared to control group and to assess the ADL, explore how ADL varied as a consequence of the severity of the disease, explore the impact of OI-type and ADL scores on the employment status. | N=97  G=58% female  A= 44y mean  D=Osteogenesis imperfecta  R=TRS National Resource Centre for Rare Diseases | - Cross-sectional quantitative study and clinical examination. - Investigating WP was one secondary outcome. | - Structured specific interviews concerning sociodemographic combinded with clinical examination, including: The Sunnaas Activities of Daily Living (ADL) Index. - Questions on WP: Not described. | - 64% working compared to general Norwegian population (GNP) =75%. 34 % not working compared to GNP=25%. - Employment status significantly associated with gender, age, educational level, and ADL score. - The study group had hgher educational level than the control group, but the employment rate was significantly lower than GP. |
| 136  Widmann et al 2001. Quality of life in osteogenesis imperfecta.  International Orthopaedics.  USA | To quantify the physical and mental health of a larger diverse adult cohort of patients with OI utilizing the SF-36 and specific functional and demographic questionnaire. | N=30 (congenital n=18, tarda n=12)  G=70% female  A=33.4y mean  D=Osteogenesis imperfecta  R=Pediatric Orthopaedics, Hospital for Special Surgery | - Cross-sectional quantitative study. - Investigating WP was one secondary outcome. | - Study specific questionnaire about diagnostic and sociodemographic aspects, including: The Functional Independence Measure (FIM), The Short-form Health Survey for Medical Outcomes Study (SF-36). - Questions on WP: Not described. | - WP 57 % employed, 32% unemployed, 17% not in work force. - Despite significant physical limitations, the results demonstrated high level of employment. |
| ***X-linked hypophosphatemia (XLH) (ORPHA:89936)*** | | | | | |
| 94  Hughes et al 2020. Giving credence to the experience of X-linked hypophosphatemia in adulthood: An Interprofessional Mixed Methods study.  Journal of Patient-Centred Research and Review.  USA | To build theory about the experiences of the disease in the affected people with X-linked hypopho-sphatemia (XLH) to engage with health care providers and services, and to identify common themes that relate to and prevent effective management for this population. | N=9  G=44% female  A=43-65y, 53.6y mean  D=X-linked hypophosphatemia  R=Quinnipiac University Hospital | - Mixed method cross sectional study. - Investigating WP was one secondary outcome. | - Combining individual interview and study specific questionnaire of sociodemographic aspects including: The Depression Anxiety Stress Scales, The Short-form Health Survey for Medical Outcomes Study (SF-36), The Consumer Assessment of Healthcare Provider and Systems (CAHPS). - Questions on WP: Not described. | - 28% of were working, 72% early retirement (the condition required leaving work). - Fear of failing prevented individuals from attending work gatherings. Balancing work, family and leisure tasks experienced as difficult. |
| 51  Lo et al. 2020.  Exploring the burden of X-linked hypophosphatemia: a European multi-country qualitative study.  Quality of Life Research.  Multi-international: Finland, France, Germany, Luxembourg, the United Kingdom | To provide an in-depth, qualitative under-standing of the nature and impact of pain, stiffness and fatigue symptoms in XLH as well as the psychosocial impact of XLH as a lifelong hereditary condition. | N=30 adults  G=70% female  A=26-69 y, 40y mean  D=X-linked Hypophosphatemia  R=Patient associations  across Europe | - Cross-sectional qualitative study, - Investigating WP was one secondary outcome. | - Individual interviews conducted by the telephone, of the impact and experiences of living with the disease. - Questions on WP: Not described. | - 63% working full time, 10% part time, 13% unable to work, 10% unemployed, 3% retired. - The self-reported mildly affected participants stated that they were able to work, whilst some moderate/severely affected patients were unable to work due to ill health.Mobility problems affected work related activities. |
| 41  Seefried et al 2021.  Burden of disease associated with X-linked hypophosphatemia in adults: a systematic review.  Osteoporosis International.  Germany/United Kingdom | To collect existing evidence on the humanistic and economic burden of XLH in adults to gain an understanding of the unmet needs associated with the disease in adulthood. | N=90 articles and 44 conference abstracts, (3 articles addressed WP)  D=X-linked Hypophosphatemia | - Systematic review - Investigating WP was one secondary outcome. | - Systematic review, without explicit documentation of quality assessment (risk of bias) of the included papers, including synthesizing the results. - Questions o WP: Nor described. | - The review found 3 articles on WP, of these 2 were published before 2000. - Other aspects: Tre review Indicate that disease negatively impact WP. Early retirement and unemployment more frequent among the patients than general population. Decrased WP associated with dental and psychosocial problems. |
| 102  Theodore-Oklota et al 2018.  Quality research to explore the patient experience of X-Linked hypophosphatemia and evaluate the suitability of BPI-SF and WOMAC.  Value in Health 21.  USA | To understand the symptoms, impacts and patient experience of X-linked hypophosphatemia (XLH) and to evaluate the face and content validity of the Western Ontario and McMaster Universities Osteoarthritis Index. | N=18  G=83% female  A=20-60y, 42y mean  D=XLH  R=Support group of patients with XLH (The XLH Network) | - Cross-sectional qualitative study. - Investigating WP was one secondary outcome. | - Individual qualitative interviews, and open-ended questions using a “think aloud” methodology of patients’ expeirnces of living with the disease. - Questions on WP: Not described. | - 61 % were working, 22% not working because of disease, 11% full-time homemakers, 6% students. - XLH characterized with a number of more distal symptoms such as changes in their work. Many reported that fatigue/tiredness was a problem at work. |
| ***Primary bone dysplasias (short stature, mixed populations) (ORPHA:364526)*** | | | | | |
| 137  Dhiman et al 2016.  Factors associated with health-related quality of life (HRQOL) in adults with short stature skeletal dysplasias.  Quality of life research.  USA | To update and clarify knowledge about the HRQOL of adults with short stature skeletal dysplasias (SD). | N=189  G=60% female  A= y>18  D=Short stature skeletal dysplasia  R=Members of Little People of America | - Cross-sectional quantitative study (online survey). - Investigating WP was one secondary outcome. | - Study specific questionnaire on sociodemographic and clinical aspects, including: The Short-form Health Survey for Medical Outcomes Study (SF-36). - Questions on WP: Not described. | - 54,5% working full/part time, 11,6% students, 6,4% retired/not looking for job, 23,8% were disabled, 3.7% unemployed/looking for work. |
| 138  Johansen et al 2007. Health status in adults with short stature: a comparison with the normal population and one well known diagnosis.  Orphanet Journal of Rare Diseases.  Norway | To examine the subjective health status of adults with short stature compared to the general population/ rheumatoid arthritis (RA). Explore associations between age, gender, height, educational level and health status. | N=44  G=73% females  A=16-61y, 36,4y mean  D=Various skeletal dysplasias  R=Clinic, TRS National Resource Center for Rare diseases. | - Cross-sectional quantitative study (postal). - Investigating WP was one secondary outcome. | - Study specific questionnaire on sociodemographic and clinical aspects including: The Short-form Health Survey for Medical Outcomes Study (SF-36). - Questions on WP not described. | - 43% employed, 34% students, 23% disability pension. |
| 114  Shakespeare et al 2010.  No laughing matter: medical and social experiences of restricted growth. Scandinavian Journal of Disability Research.  United Kingdom | To generate rich data about the life experience of people with restricted growth, covering both medical and social aspects. | N=81 (50=interviews)  G=76% female  A=16-75 y  D=Various skeletal dysplasias  R=from three geographical areas in the north of England | - Mixed method study cross sectional study. - Investigating WP was one secondary outcome. | - Combining qualitative individual interviews with study specific questionnaire about sociodemographic aspects, including: The Short-form Health Survey for Medical Outcomes Study (SF-36). - Questions on WP: Not described. | - 57 % in paid work, 16 % on permanently sick leave/disabled, 27% not described? - Respondents did not appear to experience major barriers to finding employment. Restricted growth people strongly valued employment, not least because it was a major marker of independence. |
| ***Achondroplasia (ORPHA:15)*** | | | | | |
| 115  Cortinovis et al 2011.  The Daily Experience of People with achondroplasia.  Applied Psychological Health and Wellbeing.  Italy | To investigate the daily experience of adults with achondroplasia in work, family, and relationships and to detect resources and opportunities for social integration and personal growth. | N=18  G=56% female  A=35y mean  D=Achondroplasia  R=Members of AISAC–the Italian User Association. | - Mixed method cross sectional study. - Investigating WP was one secondary outcome. | - Combing the Experience Sampling Method for collecting quantitative data on sociodemographic and clinical aspects, with the Flow Questionnaire and Life Theme Questionnaire for collecting qualitative data. - Questions on WP: Not described. | - 83% working, mainly as office staff (44%), 11% student, 6% was temporarily not working because of health problems. - Experienced that WP was a key resource to achieving well-being and social integration. |
| 139  Fredwall et al 2020.  High prevalence of symptomatic spinal stenosis in Norwegian adults with achondroplasia: a population-based study. Orphanet Journal of Rare Diseases.  Norway | To describe the prevalence of symptomatic spinal stenosis in Norwegian adults with achondroplasia, and to explore the impact of symptomatic spinal stenosis on physical functioning. | N=50  G=46% female  A=16-87y, 41y mean  D=Achondroplasia  R=TRS, National Resource Centre for Rare Disorders’ database, and four University Clinics | - Cross-sectional quantitative study and clinical examination, including historical data. - Investigating WP was one secondary outcome. | - Study specific questionnaire on clinical and sociodemographic aspects, including: The Health Assessment Questionnaire (HAQ), verified by clinical quantitative interviews. - Questions on WP: Not described. | - 26% working full time, 8% working part time, 22% student, 4% work-rehabilitation, 10 % age pension, 30 % disability benefits.. - Diseases symptoms and symptomatic spinal stenosis associated with decreased WP. - High prevalence of urinary and bowel incontinence may impact productivity at the work place. |
| 140  Gollust et al 2003. Living with achondroplasia in an average –sized world. An assessment of quality of life.  American Journal of Medical genetics.  USA | To examine quality of life (QoL) in affected individuals and incorporate QoL-data reported by affected individuals and relatives to understand experiences of living with achondroplasia. | N=189  G=67% female  A=19-89y, 40,5y mean  D=Achondroplasia  R=National Human Genome Research Institute | - Cross-sectional quantitative study. - Investigating WP was one secondary outcome. | - Study specific questionnaire about sociodemographic and clinical aspects, including: The Rosenberg Self-esteem scale, The Ferrans and power quality of life index (QLI). - Questions on WP: Not described. | - 53% were employed full time.(rest not reported) - Individuals affected with achondroplasia had lower annual income, less education, and were less likely to be married. |
| ***Diastrophic dysplasia (Diastrophic dwarfism) (ORPHA:628)*** | | | | | |
| 141  Kruger et al 2013. Health-related quality of life and socioeconomic situation among diastrophic dysplasia patients in Finland.  Journal of Rehabilitation Medicine.  Finland | To gain a comprehensive view of the quality of life and socioeconomic conditions in a more representative sample of patients with diastrophic dysplasia (DD) than previously presented. | N=68  G=63% female  A= > 18y  D=Diastrophic dysplasia (DD)  R=the Helsinki University Central Hospital (HUCH) | - Cross-sectional quantitative study (postal). - Investigating WP was one secondary outcome. | - Study specific questionnaire of sociodemographic and clinical aspects, including: The Health Assessment Questionnaire (Finn-HAQ), The Short-form Health Survey for Medical Outcomes Study (SF-36). - Questions on WP: Questions related to employment status, household income. | - 40% employed, 6% unemployed, 6% student, 44% disability pension, 4% pension. - In their daily life patients with DD have marked physical difficulties, which affect their quality of life, participation in society, activity in the work force and their financial situation. |
| ***Fibrous dysplasia of bone (ORPHA:249)*** | | | | | |
| 142  Kelly et al 2005. Physical function is impaired but quality of life preserved in patients with fibrous dysplasia of bone.  Journal of [Bone](https://www.sciencedirect.com/science/journal/87563282).  USA | To examine the impact of the scope and extent of the skeletal disease on quality of life in adults and children with fibrous dysplasia (FD). | N=56 adults and 22 children  G=66% female  A=14-86y,33,7y mean  D=Fibrous dysplasia  R=National institute of Health | - Cross-sectional quantitative study (postal). - Investigating WP was one secondary outcome. | - Study specific questionnaire about medical and sociodemographic aspects, included: The Short-form Health Survey for Medical Outcomes Study (SF-36). - Questions on WP: Not described. | - 54 % were working, 30 % students and 11% disabled, and 5 % not in the workforce. - The high level of education and employment shows that functional limitations need not prevent FD patients from living independently and working. |
| **Rare genetic hematologic diseases (ORPHA:158300)** | | | | | |
| ***Hemophilia (Haemophilia) (ORPHA:448)*** | | | | | |
| 95  [Barlow](https://www.sciencedirect.com/science/article/abs/pii/S0738399107002455#!) et al 2007. Living with haemophilia and von Willebrand's disease: A descriptive qualitative study.  Patient Education and Counseling.  United Kingdom | To describe the experience of living with bleeding disorders and to identify the associated salient issues from the perspectives of people living with [haemophilia](https://www.sciencedirect.com/topics/medicine-and-dentistry/haemophilia) or [von Willebrand's disease](https://www.sciencedirect.com/topics/medicine-and-dentistry/von-willebrand-disease). | N=9  G=0% female (100% male)  A=28-84y  D=Haemophilia and von Willebrand's  R= The Haemophilia Society | - Cross-sectional qualitative study. - Investigating WP was one secondary outcome. | - Individual qualitative interviews with nine members of The Haemophilia Society. - Questions on WP: Not described. | - Many experienced lack of understanding and discriminated by management and colleagues at work. Many felt that their bleeding disorder had impacted on their education, work, social activities and family life. |
| 143  Batt et al 2018. Patient-reported outcomes and joint status across subgroups of US adults with hemophilia with varying characteristics: Results from the Pain, Functional Impairment, and Quality of Life (P-FiQ) study.  European Journal of Hemophilia.  USA | To evaluate differences in overall health-related QoL, pain, function, and joint status between P‐FiQ study subgroups. | N=381  G=0% female (100% male)  A=34y mean  D=Hemophilia  R=15 hemophilia treatment center | - Cross-sectional quantitative study and clinical examination. - Investigating WP was one secondary outcome. | - Study specific questionnaire of sociodemographic factors and clinical aspects including several PRO instruments: EuroQoL 5L (EQ-5D-5L), Brief Pain Inventory v2 Short Form (BRI), Hemophilia Activities List (HAL), Hemophilia Joint Health Score (HJHS). - Questions on WP: Not described. | - 77% working, 17 % not working. 6% not reported. - Not working was associated with greater pain severity/interference and functional impairment. |
| 103  Beeton et al 2005.  An exploration of health-related quality of life in adults with haemophilia- a qualitative perspective.  Journal of Haemophilia.  United Kingdom | To investigate concepts associated to QoL. | N=19 (11 interviews and 7 focus groups)  G=0% female (100% male)  A=38-72y, 50y mean  D=Severely affected with haemophilia A and B  R=the Royal Free Hospital | - Cross-sectional qualitative study. - Investigating WP was one secondary outcome. | - Qualitative individual interviews and focus groups interviews of the patients’ experinces of living with the disease and QoL - Questions on WP: Not described. | - 54% were working. 27% were not working due to haemophilia, and 18% were retired. - Participants experienced often success in workplace. Several participants appeared to have an inner drive to ambitions in the workplace, and did not feel that haemophilia restricted their ambitions. |
| 104  Brodin et al 2015.  Persons with haemophilia in Sweden- experiences and strategies in everyday life. A Single Centre Study.  PLoS One.  Sweden | To explore the experiences of adults living with severe or moderate haemophilia and their coping strategies at a single centre in Sweden. | N=14  G=0% female (100% male)  A=19-80 y, 42y mean  D=Haemophilia i  R=Haemophilia Treatment Centre at Sahlgrenska University Hospital | - Cross-sectional qualitative study. - Investigating WP was one secondary outcome. | - Qualitative individual interviews about experiences and strategies of living with haemophilia. - Questions on WP: Not described. | - 42% working, 21% studying, 14% sick leave, 21% disability pension/retired. - All stated that their closest colleagues at work knew about their condition, and experienced that as a benefit. Work and education in this groups seems comparable with the general population. |
| 47  Brown et al 2020.  The societal burden of haemophilia A. A snapshot of haemophilia A in Australia and beyond.  Journal of Haemophila.  Australia | To examine the epidemiology and societal burden of people with haemophilia A in Australia with particular focus on men with the disorder. | N=Not reported  (9 articles addressed work participation)  D=Haemophilia (HA) | - A snapshot review. - Investigating WP was one secondary outcome. | - Review on target literature and data review published in Australia and international the last 10 years. - Questions on WP: Nor described. | - In Australia the rate of full time WP among adults with HA is 75 % of that for the general population, while part-time WP is 35 % higher. - 7-9 % are unemployed, 4-13 % retire early due to long term disability. - Reduced productivity due to the disease was reported. |
| 144  Buckner et al 2018. Assessments of pain, functional impairment, anxiety, and depression in US adults with hemophilia across patient-reported outcome instruments in the Pain, Functional Impairment, and Quality of Life (P-FiQ) study.  European Journal of Haematology.  USA | To report detailed patient‐reported outcomes (PRO) assessments related to health related QoL in participants in the Pain, Functional Impairment, and Quality of Life (P‐FiQ) study. | N=381  G=0% female (100% male)  A= 34y mean  D=Hemophilia A or B  R=Fifteen US outpatient clinic during comprehensive care visits | - Cross-sectional quantitative study. - Investigating WP was one secondary outcome. | - Study specific questionnaire on sociodemographic aspects and clinical aspects, including 5 PRO instruments: EQ-5D-5L, Brief Pain Inventory v/2 Short form (BPI), International physical Acitivity Questionnaire (IPAQ), The Short-form Health Survey for Medical Outcomes Study (SF-36v2), Hemophilia Activity List (HAL) - Questions on WP: Not described. | - 77 % were working (rest not reported). - Physical problems were associated with limited work-activities (69%). - Most participants (70% to 74%)-experiencing pain interference with WP the past week. |
| 42  Cassis et al 2012. Psychosocial aspects of haemophilia: a systematic review of methodologies and findings.  Journal of Haemophilia.  Spain | To review the current literature on psychosocial stressors, and to summarize the available evidence and identify areas where the evidence is heterogeneous, controversial and limited. | N=24 articles, of these 5 addressed WP  D=Haemphilia | - Systematic review. - Investigating WP was one secondary outcome. | - Systematic literature review of relevant literature, no quality assessment of included articles. - Questions on WP: Not described. | - People with mild to moderate haemophilia seems to compare well to the general popluliion in employment, althoug those with severe disease are less involved in full-time work. - In studies of Netherland: patients participated less in full time paid work than general male. In Australia approximately 70 % had been in permanent employment some time. |
| 60  Chu et al 2018. Risk of major comorbidities among workers with hemophilia: A 14-year population-based study.  Medicine.  Taiwan | To compare the incidence and risk factors of the major comorbidities of 411 workers with hemophilia enrolled in Taiwan’s National Health Insurance Research Database between 1997 and 2010 with an age- and sex-matched general population. | N=570 (411 workes and 159 non-workers) with hemophilia  G=0% female (100% male)  A=>16y, 32y mean  D= Hemophilia  R=Taiwan’s National Health Insurance | - Prospective study. - Investigating WP was the primary aim/ outcome. | - Study specific questionnaire on sociodemographic and clinical aspects, and comorbitidity. - Questions on WP: About work status and work history. | - 72% were working (rest not working) - Self-employed workers, farmers and workers with low-income jobs were found to be in a greater risk for hemorrhagic, arthrosis /arthopathy. The risk of comorbidities were significantly higher in workers with hemophila, than non-workers with the disease. - Of the 411 workers, most of the patients (64,48%) were aged 20-40 y. |
| 145  Croteau et al 2020. Awareness, care and treatment in tbesity management to inform haemophilia obesity patient empowerment (ACTION TO HOPE): Results of a survey of US patients with haemophilia and obesity (PwHO) and their partners and caregivers.  Journal of Haemophilia.  USA | To identify patient and caregiver insights on the unique challenges of PwHO. | N=124  G=29% female  A=>18 y  D= Haemophilia  R=The ACTION-TO-HOPE study | - Cross sectional quantitative study (online). - Investigating WP was one secondary outcome. | - Study specific questionnaire of more than 100 items, on sociodemographis, clinical aspects, and insight in the patients needs, including precoded (multiple-choice) closended questions, dichotomous (yes/no) questions and rating scales. - Questions on WP: Not described. | - 32% worked full time work, 8% worked part time, 12% self-employed, 11% not employed but looking job, 4% not employed, not looking for job, rest not descried. - About employment type: 9 % light manual labour, 34% manual labour, 9% strenuous labour, 43% office work/studying, 6% mainly inactive. |
| 98  Curtis et al 2015. Young adults with hemophilia in the U.S: demographics, comorbidities, and health status.  American Journal of Hematology.  USA | To investigate employment status, insurance, health-related QoL, and prevalence of the following comorbidities: pain, range of motion limitation, overweight/ obesity, and viral status. | N=141  G=0 % female (100% male)  A=18-34y  D=Hemophilia A (n=103) and hemophilia B (n=38)  R=Hemophilia special care centres. | - Prospective study. - Investigating WP was one secondary outcome. | - Combination of self-reported and clinical chart review data, with study specific questionnaire of sociodemographic and clinical aspects, included: The Short-form Health Survey for Medical Outcomes Study (SF-12). - Questions on WP: Not described. | - 44% working full time, 23% working part time, 19% unemployed, 14% student. - Lower WP signicantly associated with the disease compared to the general population. |
| 91  den Uijl et al 2009. Clinical outcome of moderate haemophilia compared with severe and mild haemophilia.  Haemophilia.  Netherlands | Compared self‐reported burden of disease in moderate haemophilia to severe and mild haemophilia. | N=1066  G=0 % female (100% male)  A=36y mean  D=Hemopholia (44% severe, 16% moderate, 39% mild),  R=The Dutch Haemophilia Society and all haemophilia centres in the Netherlands, | - Cross-sectional quantitative study. - Investigating WP was one secondary outcome. | - Study specific questionnaire (the fifth Haemophilia in the Netherlands questionnaire) on sociodemographic and clinical aspects, including: The Short-form Health Survey for Medical Outcomes Study (SF-36). - Questions on WP: About work and disability (difficultires perfoming activities comprised employment and occupation disability). | - 84% (mild haemophilia)/78% (moderate)/ 75% (severe) were working full/part time. Rest not reported. - Mild haemophila were associated with higher rate of WP and less work disability. |
| 52  Flood et al 2014. Illustrating the impact of mild/moderate and severe haemophilia on health-related quality of life: Hypothesised conceptual models.  European Journal of Haematology.  Multi-international  Germany (n=9), Spain (n=11), USA (n=11) | To understand the impacts of haemophilia and its treatment from the patient perspective and to inform the development of comprehensive health‐related quality of life (HRQL) conceptual models. | N=31 (focus groups)  G=0 % female (100% male)  A=37.7y mean  D=Haemophilia  R=Oediatric haemophilia centres | - Cross-sectional qualitative study. - Investigating WP was one secondary outcome. | - Qualitative focus groups interviews to support the validity of the conceptual models. - Questions on WP: Not described. | - 29% worked full time, 16% part time. 13% homemakers, 26 %, 10 % disabled, 6% others. - Many experienced that haemophilia negatively impacted the ability to lead a normal school or work life. They had to take significantly more sick days from job than their colleagues, due to complications related to their bleeds. |
| 53  Forsyth et al 2014. Haemophilia Experiences, Results and Opportunities (HERO) Study: survey methodology and population demographics.  Haemophilia.  Multi-interational: conducted in 10 countries. | To describe the multinational HERO quantitative study, with particular focus on the survey methodology and the demographic characteristics of adult haemophilia. | N=675  G=0 % female (100% male)  A=18-86y, 36 y mean  D=Haemophilia (moderate to severe)  R= Multinational hemophilia organizations | - Cross-sectional quantitative study (online web-based, e-mail or Web page). - Investigating WP was one secondary outcome. | - Study specific questionnaires about sociodemographic and diagnosis aspects, including: The EuroQality of life (EQ-5D-3L), The Well-being Index (WHO-5), The Male Sexual Health Questionnaire (MSHQ). - Questions on WP: Not described. | - 60 % working full/part time, 13 % unable to work due to long term disability, 1 % homemaker, rest not described. - Of those working 45 % had office work, 20 % light, 14% moderate and 4 % strenuous manual labour. - Impact of haemophilia on employment: very large impact (27%), moderate impact (25 %), small impacted l (28 %), no negative impact (19%). |
| 92  Forsyth et al 2015. Associations of quality of life, pain, and self-reported arthritis with age, employment, bleed rate, and utilization of hemophilia treatment center and health care provider services: results in adults with hemophilia in the HERO study.  Patient Preference and Adherence.  Canada | - To examine potential associations between HRQoL, pain interference, and self-reported arthritis and age, employment, activity, bleed frequency, and hemophilia treatment center and health care professional utilization. | N=675  G=0% female (100% male)  A= > 18  D=Haemophilia. (moderate to severe)  R=National hemophilia organizations | - Cross-sectional quantitative study (online web-based, e-mail or Web page). - Investigating WP was one secondary outcome. | - Study specific questionnaire study on sociodemographic aspects and pain, including: The EuroQality of life (EQ-5D-3L scale). - Questions of WP: Not described. | - 60% were employed full/part time, 8% looking for jobs, 13 % unable to work due to longterm disability, rest not described. - Increased disability and pain were associated with lower WP. |
| 61  Hartl et al 2008. The impact of severe haemophilia on the social status and quality of life among Austrian haemophiliacs.  Journal of Haemophilia.  Austria | To obtain data on the social status of Austrian haemophilia and to compare these with an age- and sex-matched reference population. | N=53  G=0% female (100% male)  A=Mean 36,7y D=Haemophilia. R=Two Austrian haemophilia Centres | - Cross-sectional quantitative study and clinical examination. - Investigating WP was the primary aim/ outcome. | - Study-specific questionnaire on clinical and sociodemographic aspects including: The Short-form Health Survey for Medical Outcomes Study (SF-36). - Questions on WP: Work status was classified according to the Austrian Social Security: employed. | - 64% employed/in training, 34% unemployed, rest not described. - Significantly fewer patients were in the active work process (either employed or in training) compared to controls (64% vs 90%), due to no difference in educational level. - 47% had white collar work, 8% blue collar work, 9 % were self-employed, 23% retired (15 % early retirement). |
| 146  Iannone et al 2012. revalence of depression in adults with haemophilia.  Journal of Haemophilia.  USA | To assess the prevalence of depression in adults with haemophilia treated at the Arizona Haemophilia and  Thrombosis Center (AzHTC) and to assess risk factors associated with depression. | N=41  G=0% female (100% male)  A=18-80y, 37y mean  D=Haemophilia  R=Haemophila Center. | - Cross-sectional quantitative study and clinical examination. - Investigating WP was one secondary outcome. | - Study specific questionnaire on sociodemographic, including: The Depression Fact Sheet and health, The Patient Health Questionnaire (PHQ-9), The Universal Data Collection for persons with Bleeding Disorder Quality of life (UDC-QoL). - Questions on WP: Not described. | - 54 % working, 46% not working of these 32% receiving disability pension. - Unemployment was significantly associated with depressive symptoms. - Treatment of depression may lead to improved adherence, and may improve social outcome and higher employment rate. |
| 147  Kempton et al 2018. Factors associated with pain severity, pain interference, and perception of functional abilities independent of joint status in US adults with haemophilia: Multivariable analysis of the Pain, Functional Impairment, and Quality of Life ((P-FIQ) study.  European Journal of Haematology.  USA | To access the associations of covariate, patients demographic and clinical characteristics with perceptions. | N=240  G=0% female (100% male)  A= ≥18 y, 32y median  D=Haemophilia  A or B  R=Clinic, hemophilia treatment center | - Cross-sectional quantitative study, prior to care visit. - Investigating WP was one secondary outcome. | - Study specific questionnaire on sociodemographic and clinical factors, including PRO instruments: EuroQoL 5L (EQ-5D-SL), disease specific aspects: HAL and Hemophilia Joint Health scan: HJHS. - Questions on WP: Not described. | - 65 % were employed.(rest not reported) - More pain were associated with unemployment status. The most significant predictors for functional impairment were older age, unemployment, severe haemophila and greater pain. |
| 112  Kempton et al 2020. Validation of a new instrument to measure disease-related distress among patients with haemophilia. Clinical Haemophilia.  USA | To develop and validate a measure of Hemophilia-Related Distress Questionnaire (HRDq). | N=126  G=9,5% women  A=32,7y mean  D=Haemophilia  R=From 15 hemophilia Centres | - Validating of instrument study - Investigating WP was one secondary outcome. | - Study specific questionnaire on sociodemographic aspecs, including: The EQ-5D-5L, The Haem A-QoL, The NCCN Distress Thermometer Kessler +6, Patient Health Questionnaire -9 (PHQ-9), The PROMIS-29 profile v2.0, The Brief Pain Inventory (BPI). - Questions on WP: The Work Productivity and Activity Impairment Questionnaire, (WPAI). | - 32,5 % working, 12 % unemployed/disability support, rest not repored.. - WPAI total work activity impairment strongly correlated with distress, pain, andQoL - 88% did not receive disability support and 11,9 % received disability support. |
| 148  Naous et al 2019. The impact of haemophilia on the social status and the health‐related quality of life in adult Lebanese persons with heamophilia.  Journal of Haemophilia.  Lebanon | To assess the impact of haemophilia on the patients daily life and social function compared to aged-matched healthy controls HRQoL and social status of adult Lebanese persons with haemophilia compared to a reference population | N=60  G=0% female (100% male)  A=18-61y, 33.6y mean  D=Haemophilia  R=Lebanese Association of Hemophilia Centres | - Cross-sectional quantitative study. - Investigating WP was one secondary outcome. | - Study specific questionnaire with four axes: sociodemographics, the disease, quality of life and additional questions, including: The Short-form Health Survey for Medical Outcomes Study (SF-36). - Questions on WP: On socio-economic level of the participants according to work status. | - 71,2 % working , 18,6% students, 10.2% unemployed. - Patients were more likely to have a job requiring physical activity than controls (55.9% vs 31.4%) and more likely to be unemployed (10.2% vs 1.0%). - Of those working, 40,7% were manual workers/drivers, 28,8% were bank employers/nurse etc,. 1,7 % were medical doctors. |
| 54  O'Hara et al 2019. Disease burden and remaining unmet need in patients with haemophilia A treated with primary prophylaxis.  Clinical Haemophilia.  Multi-national: France, Germany, Italy, Spain and United Kingdom | To compare the quality of life and work related function in people with haemophilia A, and the general population. | N=55  G=0% female (100% male)  A=18-35 y  D=Haemophilia  R= The Cost Haemophilia in Europe a sociaoeconomic Survey(CHESS-database) | - Cross-sectional quantitative study. - Investigating WP was one secondary outcome. | - Study specific questions of clinical, economic and sociodemographic factors, including: The AuroQoL, EQ-5D-3L. - Questions on WP: The Work Productivity and Activity Impairment Questionnaire (WPAI). Qustions on how the disease impact WP. | - 44% were working, rest not reported. - WP and productivity lower than for the general population. - 26 % reported mean presenteeism, 16.2% work impairment, 23.1% activity impairment |
| 55  Schram et al 2002. Clinical outcomes and resource utilization associated with haemophilia care in Europe.  Journal of Haemophilia.  Multicenter:10 European Centers | To compare outcomes and resource utilization associated with on-demand and prophylactic factors substitution methods in European haemophilia partients. | N=1021  G=0% female (100%male)  A=11-83 y, mean 34,8 y.  D=Haemophilia  R=Haemophilia Centres in Europe. | - Cross-sectional quantitative study and clinical examination. - Investigating WP was one secondary outcome. | - Study specific questionnaire on sociodemographic aspects, medical history, and healthcare resource utilization, including: The Short-form Health Survey for Medical Outcomes Study (SF-36). - Qustions on WP: Not described. | - 32,3% in white collar work, 43,6% in blue collar work, 24,1% unemployed. - LowereWP associated with the number of joint bleeds. |
| 62  Smith et al 2019. Vocational experiences and career support opportunities among Canadian men with moderate and severe haemophilia.  Journal of Haemophilia.  Canada | To provide haemophilia treatment centers with guidance for the potential development of appropriate and timely interventions related to employment and vocational counselling and supports. | N=75 (in survey), 13 (in focus groups)  G=0% female (100%male)  A=>19 y.  D=Haemophilia  R=Haemophilia treatment centres | - Mixed method cross sectional study. - Investigating WP was the primary aim/ outcome. | - Combination of study specific quantitative questionnaire on sociodemographic and clinical aspects combinded with focus group interviews. - Questions on WP: Current employment situation and future career, how the disease impact employment, and barriers and possibilities of maintaining employment. | - 60 % were working, 11% actively seeking job, 11% full-time student, 16% disability pension, 4% retired. - 47 % reported haemophila had small negative impact, 27% felt it had moderate impact and 13 % very large impact on WP. - One third had received some form of employment related advice. |
| ***Congenitial factor VII deficiency (ORPHA:327)*** | | | | | |
| 149  Peltier et al 2020. Psychosocial impact and disease management in patients with congenital factor VII deficiency.  Journal of Blood Medicine.  USA | To identify the burden of congenital factor VII deficiency on patients and caregivers through a better understanding of the management and psychosocial impact of this disease. | N=25 patients (20 caregivers)  G=56 % female A=18-67y  D=Congenital factor VII deficiency  R=Health Education Services | - Cross-sectional quantitative study (online survey). - Investigating WP was one secondary outcome. | - Study specific questionnaire on sociodemographics, diagnosis and treatment, including: The ISTH Bleeding Assessment Tool (BAT). - Questions of WP: Not described. | - 53% working full time, 13% self-employed, 13% working part time, 11% not working, 4% students, 4% retired. - Level of presenteeism were double compared to general population - 16% reported losing or resigning from a job in adulthood as a direct result of their disease. 12% stated that their employer was not understanding of their disease. |
| ***Chronic coagulation disorders (ORPHA: 183654)*** | | | | | |
| 116  Talaulikar et al 2006. Health-realted quality of life in chronic coagulation disorders.  Journal of Haemophilia.  Australia | To study the quality of life in patients with chronic coagulation disorders in Australia. | N=30  G=0% female (100% male)  A=2-83y, 36.5 y mean  D=Chronic coagulation disorders  R=Canberra Hospital | - Mixed method cross sectional study. - Investigating WP was one secondary outcome. | - Semistructuresdinterviews combined with Quantiative measurement: The Short-form Health Survey for Medical Outcomes Study (SF-36), and McGill Short-Form Pain Questionnaire. - Questions on WP: Not described. | - 33% had held permanent jobs at some times of their life. - Many experienced that the disease had great negative impact on the possibility to work. The patients who perceived that they would encounter difficulites at the work place were between 23-28 years. |
| **Rare genetic neurological disorders (ORPHA:98000)** | | | | | |
| ***Charcot-Marie-Tooth disease (hereditary motor and sensory neuropathy type 1) (ORPHA:99946)*** | | | | | |
| 105  Arnold et al 2005. Psychosocial issues that face patients with Charcot‐Marie‐Tooth disease: The role of genetic counseling.  Journal of Genetic Counseling.  United Kingdom | To describe the psychosocial issues surrounding individuals with Charcot‐Marie‐Tooth disease, with the goal to determine ways to help genetic counsellors to counsel and support this patient groups more effectively. | N=14  G=64 % female  A=32–74 years  D=Charcot‐Marie‐Tooth disease  R=Clinic, New York medical centre and Greater New York Support Group Meeting | - Cross-sectional qualitative study. - Investigating WP was one secondary outcome. | - Individual interviews on sociodemographic, clinical, psychosocial and disability. - Questions on WP: Not described. | - 64% were working, 36 % unemployed. - Several experienced difficulties to WP. It was emphasized that genetic counsellors need to be aware of genetic testing for the reason of insurance and employment purposes. |
| 56  Lafarge et al 2014. Living with Charcot-Marie-Tooth disease: a qualitative analysis.  [British Journal of Neuroscience Nursing.](https://www.magonlinelibrary.com/doi/abs/10.12968/bjnn.2014.10.5.226)  Multi-national: United Kingdom, US and Australia | To examine the relationship between illness perceptions, coping strategies and quality of life. | N=194  G=66,8% female  A=20-86,y, 52,7y mean  D=Charcot-Marie-Tooth disease  R=CMT charity, muscle group | - Mixed method cross sectional study. - Investigating WP was one secondary outcome. | - Study specific questionnaire on medical and sociodemographic aspects, including open-ended questions about experiences of living with Charcot‐Marie‐Tooth Disease. - Questions on WP: Not described. | - The participants’ experienced that the disease had detrimental impact on work ability, and often associated with financial concern. |
| 150  van der Linden et al 2007.  Ambulatory disabilities and the use of walking aids in patients with hereditary motor and sensory neuropathy type 1.  Journal of Disability & Rehabilitation.  Netherlands | To determine the level of ambulatory disability and the use of walking aids in well-ambulant hereditary motor and sensory neuropathy type I (HMSN I) patients, and to identify the related demographic, physical and psychological variables. | N=75  G=58% female  A=20–58 y, 46,8y mean  D=HMSN 1  R=The Dutch Neuromuscular Disease Association | - Cross-sectional quantitative study, including clinical examination. - Investigating WP was one secondary outcome. | - Study specific questionnaire on clinical and sociodemographic aspects, including: The Sickness Impact Profile, Medical Research Council, The Checklist Individual Strength, EuroQoL, The Checklist Individual Strength (CIS). - Question on WP: Not described. | - 62. 7% were working, 4% household activites, 10,7% study/school, the rest not reported. - 25% declared partially of completely incapacitated to work. |
| 151  Videler et al 2009. Limited upper limb functioning has impact on restrictions in participation and autonomy of patients with hereditary motor and sensory neuropathy 1A.  Journal of Rehabilitation Medicine.  Netherlands | To evaluate upper limb function, restrictions on participation and the independent contribution of upper and lower limb disability to participation in hereditary motor and sensory neuropathy. | N=49  G=25% female  A=18-70 y, 46,8y mean  D=Hereditary motor and sensory neuropathy 1A  R=Rehabilition clinic | - Cross-sectional quantitative study. - Investigating WP was one secondary outcome. | - Study specific questionnaire on medical and sociodemographic aspects. - Questions on WP: The Michogan Hand Outcomes Questionnaire (one domain “work performance”), The Impact on Participation and Autonomy Questionnaire (one domain on work/education). | - 65 % were working, 33 % not working and 2 % not reported. - Restrictions on participation were perceived particularly in the domains of work. Insufficient participation (poor/very poor) reported in the domain of getting and keeping work. |
| ***Duchenne muscular dystrophy (ORPHA:98896)*** | | | | | |
| 152  Rahbek et al 2005. Adult life with Duchenne muscular dystrophy: Observations among an emerging and unforeseen patient population.  Pediatric Rehabilitation.  Denmark | To review existing information and describe body functional, social participatory and quality of life profiles of the ordinary adult Danish Duchenne muscular dystrophy patient. | N=65  G=0 % female (100% male)  A=18–42y  D=Duchenne muscular dystrophy  R=Institute for Neuromuscular Disease | - Cross-sectional quantitative study. - Investigating WP was one secondary outcome. | - Study specific questionnaire of medical and sociodemographic aspects, comprising 197 items, with 179 close-ended and 18 open-ended question. - Questions on WP: education and employment were items in the questionnaire. | - None was working. - 12 % had occupational experiences. 92 % early retirement. |
| ***Facioscapulohumeral muscular dystrophy (FSHD) (ORPHA:269)*** | | | | | |
| 106  Bakker et al 2017. It’s not just the physical: a qualitative study regarding the illness experiences of people with facioscapulohumeral muscular dystrophy.  Journal of Disability &Rehabilitation.  Netherlands | To provide insight into the illness experiences of people with facioscapulohumeral muscular dystrophy in order to tailor rehabilitation programs to individual needs and expectations. | N=25  G=44% female  A=24 -77y, 56y mean  D=Facioscapulohumeral muscular dystrophy  R=Different medical and rehabilitation Centres | - Cross-sectional qualitative study. - Investigating WP was one secondary outcome. | - Semi-structured individual interviews were conducted in the patients’ home, focused on the illness experiences. - Questions on WP: Not described. | - 36,0% were working (of these 2 were working full time) - The impact of the disease on daily life was often related to the participants’ ability to work. Some had to make adjustments in their work, others had to change their jobs, and some had to give up work. |
| ***Limb-girdle muscle dystrophy (ORPHA:466921)*** | | | | | |
| 117  Aho et al 2016. Health perceptions of young adults living with recessive limb‐girdle muscular dystrophy.  Journal of Advanced Nursing.  Sweden | To describe health perceptions related to sense of coherence among young adults living with recessive limb‐girdle muscular dystrophy. | N=14  G=57% female  A=20‐30 y.  D=Limb‐girdle muscular dystrophy  R=Hospitals  in three different regions. | - A mixed method cross sectional study. - Investigating WP was one secondary outcome. | - Combining qualitative data from interviews and quantitative data from The Sense of Coherence Questionnaire (SOC-13). - Questions on WP: Not described. | - 28% were working full/part time, 28% students, 42 % not working/studying. - The participants emphasized the importance of having a meaningful job. |
| ***Muscular dystrophies, mixed populations (ORPHA:*206647)** | | | | | |
| 63  Bostrøm et al 2005. Sickness impact in people with muscular dystrophy: a longitudinal study over 10 years.  Clinical Rehabilitation.  Sweden | To describe changes of function in terms of sickness impact over 10 years in adult patients with different types of muscular dystrophy. | N=44 people.  G=59% female  A=47y mean  D=Myotonic dystrophy andr muscular dystrophy  R=Study in the county of Ørebro hospital. | - Prospective study. - Investigating WP was the primary aim/ outcome. | - Study specific questionnaire on sociodemographic and clinical aspects, including: The Sickness Impact Profile (the SIP), The Self-report ADL. - Questions on WP: SIP consists of items related to work. | - Changes from 1991 vs 2001: 30 % vs 14% were working full time, 18% vs 11% working part time, 5 % vs 0% unemployed, 11 % vs /2% on sick-list, 36% vs 55% full disability pension, 0%/ vs 8% retirement pension. - Higer age was associated with lower WP (disability pension increased from 36 to 55% from 2001 to 2010). |
| ***Myotonic dystrophies (ORPHA:206647)*** | | | | | |
| 153  Gagnon et al 2007. Life habits in myotonic dystrophy type 1.  Journal of Rehabilitation Medicine.  Canada | To describe and compare life habits between individuals with mild phenotypes of myotonic dystrophy; identify life habit dimensions in which accomplishment is compromised; and describe satisfaction related to life habit. | N=200  G=60,5 % female  A=20-81 y, 47y mean  D=Myotonic dystrophy type 1 (42 mild phenotypes, 158 adult phenotypes)  R=Neuromuscular Centres | - Cross-sectional quantitative study. - Investigating WP was one secondary outcome. | - Study specific questionnaire on sociodemographic and clinical factors including: The short version of the Assessment of Life Habits (LIFE-H). - Questions on WP: The LIFE-H includes questions about employment/work. | - 44,5 % were holding a paid job.(the rest, not reported) - Significantly lower WP in those with severe than those with the mild phenotype. - The lowest satisfaction score was observed in the Employment and Recreation categories. |
| 118  Heatwole et al 2015.  Patient-reported impact of symptoms in myotonic dystrophy type 2 (PRISM-2). American Academy of Neurology.  USA | To determine the frequency and relative importance of the most life-affecting symptoms in myotonic dystrophy type 2 and to identify factors associated with these symptoms. | N=74  G=65% female  A= >21y, 57y mean  D=Myotonic dystrophy type 2  R=University of Rochester neuromuscular clinic | - Mixed method cross sectional study. - Investigating WP was one secondary outcome. | - Combing study specific questionnaire about sociodemographic and clinical aspects with qualitative interviews. - Questions on WP: Not described. | - 44.6 % (n=33) were working (the rest, not reported). - Unemployment was associated with a longer duration of symptoms, and less education and a higher average prevalence of all symptomatic themes. |
| 154  Laberge et al 2007. The correlation of CTG repeat length with material and social deprivation in myotonic dystrophy.  Clinical Genetic.  Canada | To explore the relationship between CTG repeats, muscular impairment, socioeconomic characteristics, and  social support in patients with myotonic dystrophy. | N=200  G=60, 5 % female  A=20–81y, 47y mean  D=Myotonic dystrophy  R=Neuromuscular Clinic (Jonquie`re, Quebec, Canada) | - Cross-sectional quantitative study, home visit. - Investigating WP was one secondary outcome. | - A study specific questionnaire on sociodemographic aspects, including: The PSS-FA, The PSS-Fr. - Questions on WP: Employment status about currently working, used to work, and never worked. | - 20% were working, 66% had worked, 14 % had never worked. - The group showed poor academic achievement, high unemployment and low family income, compared to the general population. |
| 155  Laberge et al 2013. Clinical, psychosocial, and central correltates of quality of life in myotonic dystrophy type 1 patients.  European Neurology.  Canada | To compare SF-36 scores of a large cohort of myotonic dystrophy type 1 patients to population specific normative data and to identify demographic, social, clinical, genetic, central, affective and personality factors. | N=200  G=60,5% female  A= 47y mean  D=Myotonic dystrophy type 1  R=Neuromuscular clinic of the Centre de sante`et de service sociaux | - Cross-sectional quantitative study, including clinical examination. - Investigating WP was one secondary outcome. | - Study specific questionnaire on sociodemographic factors, including: The muscle Impairment Scale (MIRS), The symptom Checklist -90 (SCL-90-R), The Daytime Sleepiness (DSS), The Fatigue Severity Scale (FSS), The Wechsler Adult Intelligence Scale (WAIS-R), The Short-form Health Survey for Medical Outcomes Study (SF-36). - Questions on WP: Not described. | - 20 % employed 80% unemployed. - Unemployment associated to lower scores in physical health function. |
| **Rare genetic developmental defect during embryogenesis (ORPHA:183530)** | | | | | |
| ***Neurofibromatosis type 1 and type 2 (ORPHA:636, 637)*** | | | | | |
| 119  [Bicudo](javascript:;) et al 2016. Quality of life in adults with neurofibromatosis 1 in Brazil.  [Journal of Genetic Counseling](https://link.springer.com/journal/10897).  Brazil | This study aimed to examine quality of life constructs among adults with neurofibromatosis 1 in Brazil. It is an exploratory, descriptive and cross-sectional study consisting of two stages, involving thirteen adult patients. | N=13  G=62% female  A=18-64 y, 39,3y mean  D=Neurofibromatosis 1  R=Medical Genetic clinic | - Mixed method cross- sectional study. - Investigating WP was one secondary outcome. | - Study specific questionnaire on sociodemographic and clinical aspects, including: The WHOQOL-100 questionnaire, combinded with individual semi-structured interviews. - Questions on WP: Such as “have you ever stopped participating in employment because of Neurofibromatosis 1”. | - 53,8 were working full time, 7% retired, 32,1% unemployed, rest homeworkers. - Some patients experienced that the diagnosis limited job opportunities and affected the professional life, either in terms of prejudice or as results of medical consequences. |
| 87  Buono et al 2021. The mediating effects of quality of life, depression, and generalized anxiety on perceived barriers to employment success for people diagnosed with neurofibromatosis type 1.  Orphanet Journal of Rare Diseases.  USA | To explore differences in the impact of psychological factors, quality of life and employment hope on barriers to successful employment between those with neurofibromatosis type 1 and matched controls. | N=105  G=66%  A=18-56 y, 43,6y mean D=Neurofibromatosis type 1 R= NF advocacy listservs (NF Network and NF Northeast) | - Cross-sectional quantitative study (email) - Investigating WP was the primary aim/ outcome. | - Study specific questionnaire on sociodemographic and clinical aspects, including: The Generalized Anxiety Disorder Scale-7 (GAD-7), The Patient Health Questionnaire (PHQ-9), The Short-form Health Survey for Medical Outcomes Study (SF-36), - Questions on WP: The employment Hope Survey-Short (EHS-14) and The Barrier to Employment Success Inventory (BESI). | - 65% were working, 5% retired, 11% unemployed, 11% received disability pension, 5% students, 1% workers` compensation. - The disease was associated with more barrier to successful employment, compared to matched controls. - Total barrier to successful employment was higher than the general population, indicating physical, mental, social and societal pressure are constrained in individuals with neurofibromatosis 1. |
| 93  Cohen et al 2015. Depression among adults with neuro-fibromatosis type 1: Prevalence and impact on quality of life.  Clinical Genetics.  USA | To measure the prevalence of depressive symptoms among a large sample of adults with neurofibromatosis 1 and to quantify the impact of depressive symptoms on quality of life. | N=498  G=73 % female  A=18-69y, 38,9y mean  D=Neurofibromatosis type 1  R=National NF1 organizations | - Cross-sectional quantitative study. - Investigating WP was one secondary outcome. | - Study specific questionnaire on sociodemographic and clinical aspects including: The Center for Epidemiologic Studies Depression scale (CESD), The Ferrans and Powers Quality of Life Index (QLI). - Questions on WP: Not described. | - 57,9 % full time work, 12.4% part time, 14.7 unemployed, 3.9% retired, 11.9 disabled for employment. - No associations were found between WP and QoL. |
| 64  Fjermestad et al 2019. Health complaints and work experiences among adults with neurofibromatosis 1.  Occupational medicine.  Norway | To examine work participation and experiences in relation to health complains among adults with neurofibromatosis 1(NF1). | N=142  G=62% females  A=32-80 y, 50,3y mean  D=Neurofibromatosis 1  R=Resource Centre for rare diseases (60%) and patient organization (40%) | - Cross-sectional quantitative study. - Investigating WP was the primary aim/ outcome. | - -Study specific questionnaire on sociodemographic and clinical aspects, including The Subjective Health Complaint Inventory (SHC). - Questions on WP: Eight items from National Labour Force survey concerning work-related experiences. | - 44% were working, 35% disability pension, 9% sick leave/work allowance, 7% on regular retirement benefits, 0.7% unemployed, 4,5 % not repored. - Subjective health complaints significantly associated to less work participation. Significantly more people with NF1 reported workplace bullying (13 versus 5%) and work as physically exhausting (74% versus 44%), than general population. - Person with NF1 experince more psychical and social problems than general population, and work domains negatively related to health complaints. |
| 156  Hamoy-Jimenez et al 2020.  Quality of life in patients with neurofibromatosis type 1 and 2 in Canada.  Neuro-Oncology Advances.  Canada | To assess factors associated with quality of life of people with neurofibromatosis type 1 (NF1) and type 2 (NF2) in Canada. | - N=184   G=55%/59% female  A= mean 33y (NF1) and 40y (NF2) D=Neurofibromatosis 1 (88%) and Neurofibromatosis 2 (12%)  R=The Elisabeth Raab Neurofibromatosis Multidisciplinary clinic | - Cross-sectional quantitative study, including clinical examination. - Investigating WP was one secondary outcome. | - Study specific questionnaire of clinical and sociodemographic aspects, including: The Short-form Health Survey for Medical Outcomes Study (SF-12), The EuroQoL 5L (EQ-5D-5L), The PROMIS pain interference, The PedsQL NF1 module, The NFTI-QOL for NF2. - Questions on WP: Not described. | - NF1 vs NF2 patients: 49 vs 50% employed, 6% vs20% unemployed, 16% vs 25% disability pension, 23% vs 5% students, 6% vs 0% other occupations. - NF1 and NF2 patients had lower employment rate than the general population, and lower score in all domains of Quality of Life. |
| 107  [Hummelvoll](javascript:;) et al 2013. Young Aaults’ experience of living with neuro-fibromatosis type 1.  [Journal of Genetic Counseling](https://link.springer.com/journal/10897).  Norway | To describe the experiences and concerns of persons living with NF1 in the early stages of adulthood, particularly related to social and psychosocial aspects. | N=15  G=53% female  A=18-37 y  D=Neurofibromatosis type 1(NF1)  R=Frambu Resource Centre for Rare Diseases | - Cross-sectional qualitative study. - Investigating WP was one secondary outcome. | - Semi-structured individual interviews, on the participants’ experiences and concerns related to the disease. - Questions on WP: Not described. | - 13 % were working full time, 20 % working part time, 27% unemployed, 27% students, 13% disability pension. - Participants experienced that unemployment contributes to low self-confidence, depressive reactions, worrying and an experience of NF1 dominating one`s life. |
| 157  Leschziner et al 2013. Sleep disturbance as part of the neurofibromatosis type 1 phenotype in adults.  American Journal of Medical Genetics. Part A.  United Kingdom | To examine the frequency of sleep disturbance in neurofibtormatosis 1 adults. | N=100  G=57 % female  A=16 -69y  D=Neurofibromatosis 1  R=National neurofibromatosis NFI service. | - Cross-sectional quantitative study. - Investigating WP was one secondary outcome. | - Study specific questionnaire on sociodemographic and clinical aspects including: The ESS scores and The PSQI. - Questions on WP: Patients were examined for occupation that might influence sleep quality. | - 47 % were working, 15% students, 35 % unemployed. - Unemployment status was significantly associated with a worse global PSQI score and worse sleep quality in the PSQI. |
| 120  [Neary et al](https://www.tandfonline.com/author/Neary%2C+Wanda+J) 2006. Psychosocial effects of neurofibromatosis type 2 (part 1): General effects.  Audiological Medicine.  United Kingdom | To probie both general effects and those specifically related to the condition`s impact on specific organ systems. | N=20  G=55% female  A=23-57 y, 46y mean  D=Neurofibromatosis type 2  R=Manchester Multidisciplinary Clinic. | - Mixed method cros-sectional study - Investigating WP was one secondary outcome. | - Combining open-ended questionnaire and individual interviews on patients’ experiences of living with the condition. - Questions on WP: The occupational experiences were elicited with different questions. | - 40% were working, 35% retired, 25% unemployed. - Participants’ experiemced that the diagnosei affected their work, and had to leave employment due to hearing difficulties. Those, working, were concerned about the future due to communication problems. |
| 158  Wolkenstein et al 2001. Quality-of-Life impairment in neurofibromatosis type 1. Cross-sectional study of 128 cases.  Archives of Dermatology.  Franch | To evaluate the impact of the severity and visibility of neurofibromatosis type 1 on QoL. | N=128  G=58.6% female  A=18-74y  D= Neurofibromatosis type 1  R= French academic dermatological and neurofibromatosis clinic. | - Cross-sectional quantitative study. - Investigating WP was one secondary outcome. | - Study specific questionnaire on sociodemographic and clinical aspects including: The Short-form Health Survey for Medical Outcomes Study (SF-36). - Questions on WP: Not descriped. | - 57,0 % were working full/part time, 9,4% retired, 5.5% unemployed, 4.7% caring for home, 9,4% others, 14.1% missing data. |
| ***Spinal muscular atrophy (SMA) (ORPHA:139518, 139525, 139547, 206580)*** | | | | | |
| 159  Belter et al 2020. Quality of life data for individuals affected by spinal muscular atrophy: a baseline dataset from the Cure SMA Community Update Survey.  Orphanet Journal of Rare Disease.  USA | To look at various PROMs that assess specific aspects of patient experience that might bee more sensitive to capturing subtle, but meaningful changes with drug therapy, in progression of spinal muscular atrophy (SMA), QoL, work productivity and fatigue. | N=185 affected patients (total of 478 included caregivers and children)  G=59.4% female  A=>18y D=SMA type 1,2,3  R=SMA patient advocacy organization world wide (self-reported database) | - Cross-sectional quantitative study. - Investigating WP was one secondary outcome. | - Study specific questionnaire of sociodemographic aspects, including PROMs: The Health Utilities Index, The Patient Reported Outcomes Measurement Information System Fatigue Short Form (PROMIS Fatigue SF). - Questions on WP: The Work Productivity, The Activity Impairment (WPAI). | - 47,9 % were working (included caregivers) - Work productivity loss negatively associated to decreased QoL Less severe SMA associated with higher QoL and work productivity. |
| 108  Wan et al 2019. Getting ready for the adult world”: how adults with spinal muscular atrophy perceive and experience healthcare, transition and well-being.  Orphanet Journal of Rare Diseases.  Australia | To qualitatively explore the lived experiences and perspectives of adults with spinal muscular atrophy (SMA), particularly as related to healthcare, transition and wellbeing. | N= 19 adults (5 parents and 1 partner)  G=63%  A=16-48y, 30y mean D= spinal muscular atrophy  R=Advertisements distributed via email and social media to members of SMA Australia | - Cross-sectional qualitative study. - Investigating WP was one secondary outcome. | - Individual interviews about experiences of the diagnosis. - Questions on WP: Not described. | - 32% were working fulltime, 16% part time, 5% causal employment, 26% students, 5% retired, 16 % unemployed. - Many experienced that vocational pursuits were valued, but fatigue and the burden of physical care prevented many with SMA from securing fulltime employment. Working from home and flexible or part-time work that facilitated participation in the labour force were appreciated. |
| 121  Jepperson et al 2010. Living and ageing with spinal muscular atrophy type 2: Observations among an unexplored patient population.  Journal of Developmental Neurorehabilitation.  Denmark | To study conditions of living, participation and diagnostic perceptions in a national population of adult persons with spinal muscular atrophy type 2 (SMA 2) | N=37 (n=29 in interview)  G=38% female  A=18-69y, 34y mean  D=SMA2  R=RCFM. registy | - Mixed methods cross sectional study. - Investigating WP was one secondary outcome. | - Combining study specific questionnaire on sociodemographic and clinical aspects comprising 150 questions, with individual interviews. - Qustions on WP: Not described. | - 48,3% were working, 14% had worked, 38% had never worked. |
| 160  Kruitwagen et al 2018. Social participation of adult patients with spinal muscular atrophy (SMA): Frequency, restrictions, satisfaction, and correlates.  Journal of Muscle & Nerve.  Netherland | To describe the frequency of, and perceived restrictions in, participation and related satisfaction levels in adults with spinal muscular atrophy (SMA) types 1–4. | N=62  G=45% females  A=20-70y, 43 y mean D=SMA 1-4  R=the Dutch patient organization and 4 Dutch Centers for Chronic Respiratory Ventilation | - Cross-sectional quantitative study, including medical examination study. - Investigating WP was one secondary outcome. | - Study specific questionnaire on sociodemographic aspects, including: The Utrecht Scale for Evaluation Rehabilitation (USER-P) and The Expanded Hammersmith Functional Motor Scale, The Hospital and Anxiety and Depression scale, The Coping Intentory for Stressful situations, The Fatigue Symptom Inventory, The Short-form Health Survey for Medical Outcomes Study (SF-36). - Questions on WP: Not described. | - 39% had paid work, of these 16 % had full time work. 43 % of men were employed, which is half as many as in the general population. - Less severe motor impairment were associated with increased WP. - 67 % had experienced restrictions in work/education. |
| ***Turner syndrome (ORPHA:881)*** | | | | | |
| 122  Boman et al 2009. Psychologival Wellbeing in women with Turner syndrome: Somatic and social correlates.  Journal of Psychosomatic Obstetrics and Gynoecology.  Sweden | To examine possible somatic and social correlates to psychological well-being in adult women with Turner Syndrome, including hormone replacement treatment. | N=63  G=100% female  A=>18y, 31,5y mean  D=Turner syndrome  R=Multidiciplinary Swedish nationalwide project | - Mixed method cross sectional study. - Investigating WP was one secondary outcome. | - Individual qualitative interviews combinded wih of two standardized self-rating scales, The Psychological General Well-being Index (PGWB), The Nottingham Health profile (NHP). - Questions on WP: Not described. | - 87 % were working, 13 % sickness leave/unemployed. - In the interview, both negative and positive experiences of the diagnoses were reported in relation to WP. |
| 48  Dorr et al 2019. Life situation of young women with Turner syndrome: Results of a questionnaire-based study in Germany.  Deutsche Medizinische Wochenschrift  Germany | To investigate psychosocial aspects in women with Turner syndrome. | N=130  G=100% female  A=25y mean  D=Turner syndrome  R=Medical clinic in Germany | - Cross-sectional quantitative study. - Investigating WP was one secondary outcome. | - Study specific questionnaire of sociodemographic, psychosocial and clinical aspects. - Questions on WP. Not described. | - 46 % were working. (the rest, not reported). - 39 % had not completed vocational training. |
| 99  Fjermestad et al 2016. A 6‐yearfollow‐up survey of health status in middle‐aged women with Turner syndrome.  Clinical Endocrinology.  Norway | To compare multiple areas of function betwee Turner syndrome and controls in a 6 years follow-up study. | N=57  G=100% female  A=40,6y mean  D=Turner syndrome  R=Frambu Resource Centre for Rare diseases | - Prospective study. - Investigating WP was one secondary outcome. | - Study specific questionnaire on sociodemographic aspects, included: The Life Satisfaction Questionnaire 11, The Rosenberg Self-Esteem. - Questions on WP: The General Nordic questionnaire for psychological and social factors in work (QPS–Nordic). | - 68 % were working (34 % worked part time), 14 % on disability pension (the rest not described) - The disease associated to decrease in work score compared to controls. - Participants reported that work was more physically challenging, and that they experienced more strain in late work life. |
| 65  Gould, et al 2013. High levels of education and employment among women with Turner syndrome.  Journal of Women's Health.  USA | To compare education levels, employment, marriage and parenting in a current cohort of women participating, to current census data for US female population. | N=240  G=100% female  A=25-67y, 38,4y mean  D=Turner syndrome  R=National Institute of Health, Clinical research Center in Bethesda | - Cross-sectional quantitative study, including clinical examination. - Investigating WP was the primary aim/outcome. | - Study specific questionnaire of sociodemographic and clinical aspects. - Questions on WP: From the Burau of Labor and Statistics. | - 80% was employed (compared to 70% in the US female population). 20% unemployed, of these 10 % were not seeking employment. - No association of age, age of diagnosis, or height with employment status. - Women with Turner syndrome currently achieve education and employment levels higher than the female US population, but are less likely to marry. |
| 161  Hanew et al 2021. The current status of 492 adult women with Turner syndrome: a questionnaire survey by the foundation for Growth Science.  Endocrine Journal.  Japan | To investigate the association between menstruation, Kaufmann therapy (menstrual induction therapy), social status (education, employment & marriage), complications, transition from pediatric to adult care, and sex chromosome. karyotype using statistical methods. | N=210 about WP (492 cases reported from their physician)  G=100% female  A=born before 1.August 1993  D=Turner syndrome  R=Medical clinic | - Cross-sectional quantitative study. - Investigating WP was one secondary outcome. | - Study specific questionnaire on sociodemographic and clinical aspects. - Questions on WP: Not described. | - 72,9% were working, 16% students, 22.8% not working. - Employment rate and length of education were significantly higher in patients that had received Kaufman therapy than among those not receiving it. No associations between employment rate and complications of presence of hearing loss. |
| 100  Krantz et al 2019. Health- related quality of Life in Turner syndrome and the Iifluence of growth hormone therapy: A 20-Year follow-up.  Journal of Clinical Endocrinology & Metababolism.  Sweden | To describe the health related HRQoL of women with Turner syndrome (TS) and how grow hormone treatment and comorbidity influence HRQoL and to compare HRQoL of women with TS with the general population. | N=178  G=100%  A=16-78y  D=Turner syndrome  R=The Turner Center, Sahlgrenska University Hospital, Gothenburg, Sweden | - Prospective study. - Investigating WP was one secondary outcome. | - Study specific questionnaire on sociodemographic aspects, including: The psychological General Well-Being index, Nottingham Health Profile. - Questions on WP: Not described. | - 81% were working, 11% sick leave/ retired, rest not reported. |
| 66  Naess et al 2009. Health status in women with Turner syndrome: a questionnaire study on health status, education, work participation and aspects of sexual functioning.  Journal of Clinical endocrinology.  Norway | To explore health status, level of education, work  participation, medical conditions, physical activity, satisfaction  with life and aspects of sexual functioning in adult Turner syndrome (TS) women and compare with a matched control group. | N=80  G=100% female  A=>18y, 34y mean  D=Turner syndrome  R=Frambu Resource center and Norwegian Turner Syndrome Association | - Cross-sectional quantitative study. - Investigating WP was the primary aim/ outcome. | - Study specific questionnaire of sociodemographics and clinical aspects, including: The Norwegian Standardized Classification of Education, The Life Satisfaction Questionnaire 11 (Lisat 11), The Short-form Health Survey for Medical Outcomes Study (SF-36). - Questions on WP: The general Nordic Questionnaire for Psychological and Social factors of Work (QPS). Questions from the Norwegian Labour Force Survey. | - Employed/students 89 %, disability pension 8%, and out of work/domestic work 2%. - Employment status was significantly associated to the disease, compared to the controls. The TS-group (68%) more often held a permanent job position than the controls (58%). - None of the women with TS were self-employed, compared to 4% in the controls, and 8 % were employed in temporary job positions compared to 19% in controls. |
| 162  Van der Hoven et al 2020.  A value-based healthcare approach: Health-related quality of life and psychosocial functioning in women with Turner syndrome.  Journal of Clinical Endocrinology.  Netherlands | To investigate different aspects of health-related quality of life (HRQoL) and psychosocial  functioning in women with Turner syndrome (TS) in order to establish new possible targets for  therapy. | N=177 (90 about work)  G= 100% female  A=≥18 years, 33y mean  D=Turner syndrome.  R=Out-patient clinic, Netherlands | - Cross-sectional quantitative study, as part of valued based health programme. - Investigating WP was one secondary outcome. | - Study specific questionnaire on sociodemographic and clinical aspects including: The EuroQol-5D (EQ-5D), The Hospital Anxiety and Depression Scale (HADS), The Checklist Individual Strength (CIS-20), The Perceived Stress Scale (PSS-10), The Fertility Quality of Life (Ferti-QoL), The Perceived Burden Questionnaires. - Questions on WP: Not described. | - 26% worked full time, 40% part time, 18% were students, 13% unemployed. - 2 % reported work-related stress, 4% reported being overburdened by work, 30% reported concerns about the future, often related to their education or work. |
| 163  Verlinde et al 2004. Health and psychosocial status of patients with Turner syndrome after transition to adulthood: the Belgian experience.  Journal of Hormone Research in Paediatrics.  Belgian | To gain insight into the adult medical and psychosocial situation, we performed a survey in young adult Turner syndrome (TS) patients. | N=102  G=100% female  A=23,4 y mean  D=Turner syndrome  R=Recruited by the BSGPE data base | - Cross–sectional quantitative study (postal). - Investigating WP was one secondary outcome. | - Study specific questionnaire concerning current medical care, health status, education, occupation and living situation. - Questions on WP: Not described. | - 45.1% were working full-time, 44,1 % students, 6.9% were unemployed, and 3.9% received state benefits. - Indicating that more TS women obtained higher education and were working compared to the general poluation. |
| ***22q11.2 deletion syndrome ( ORPHA:567)*** | | | | | |
| 57  Mosheva et al 2019. Education and employment trajectories from childhood to adulthood in individuals with 22q11.2 deletion syndrome. Journal of European Child & Adolescent Psychiatry.  Multi-national: Italia and Israel | To explore education and employment trajectories of individuals with 2q11.2deletion syndrome (DS) from childhood to adulthood in a large cohort composed of two significant samples. | N=144 adult (of 260)  G=48 % female  A=5-59y, 21,3y mean  D=22q11.2 deletion syndrome  R=The Behavioral Neurogenetics Center, Sheba Medical/patient Israel, Switzerland, France,and French-speaking Belgium | - Cross-sectional qualitative study. - Investigating WP was the primary aim/ outcome. | - Individual interviews using the Structured Clinical Interview for Axis I DSMIV. - Questions on WP: Experiences of different aspects of WP. Occupation was divided into three main groups: (1) regular employment and 2) assisted employment 3) unemployed (looking for training/job, but were to disabled to work. | - 30% were currently working, 22% assisted employment, 3% students, 37% unemployed. - Working in the open marked was significantly correlated with higher cognitive ability, having a driver licence and beeing married, compared to assisted employment or being unemployed. |
| ***Emery-Dreifuss muscular dystrophy (ORPHA:261)*** | | | | | |
| 67  Madej-Pilarczyk et al 2014. Professional activity of Emery-Dreifuss muscular dystrophy (EDMD) patients in Poland.  International Journal of Occupational Medicine & Environmental Health.  Poland | To identify factors which might influence the ability to work in EDMD patients in Poland. | N=24  G=25% female  A=18-56y, 34.4y mean  D=Emery-Dreifuss muscular dystrophy  R=Neuro­muscular Unit, Mossakowski Medical Research Centre | - Cross-sectional quantitative study. - Investigating WP was the primary aim/ outcome. | - Study specific questionnaire on sociodemographic and clinical aspects. - Question on WP: Asking about edu­cation, current and former employment, and disability certificates and pension. | - WP 54% were working, 25% never undertaken a job. - Work was associated with educational level, but not with level of physical performance or disease complications. - 23% of professionally active patients were employed in a sheltered workplace. |
| **Rare systemic and rheumatological diseases (ORPHA:98023)** | | | | | |
| ***Hereditary angioedema (HAE) (ORPHA:91378/100051)*** | | | | | |
| 164  Hews‑Girard et al 2021.  Psychosocial burden of type 1 and 2 hereditary angioedema: a single‑center Canadian cohort study.  Journal of [Allergy, Asthma & Clinical Immunology](https://aacijournal.biomedcentral.com/).  Canada | To characterize the psychosocial burden in a Canadian cohort with hereditary angioedema types 1 and 2 and to explore the impact on quality of life and work productivity. | N=17  G=76 %  A=20-63y, 43y mean  D=Hereditary angioedema type 1 or 2  R=Southern Alberta Rare Blood and Bleeding Disorders Comprehensive Care Program | - Cross-sectional quantitative study. - Investigating WP was one secondary outcome. | - Study specific questionnaire of sociodemographic and clinical aspects, including: The Depression, Anxiety, Stress Scale (DASS-21), The DSM-5 cross cutting measures, The Angioedema Quality of Life Questionnaire (AE-QoL), The Short-Form 36-item Health Survey (SF-36v2). - Questions on WP: Work Productivity and Activity Impairment Questionnaire (WPAI). | - 35% had full time work, 29% part time work, 24 % unemployed, 6% retired, 6% student. . - The disease was associated with not working, but with minmal effect. - Only one participant reported work absenteeism of 31.1% due to health problems, which translated into a work productivity loss of 27. |
| 58  Mendivil et al 2021. Clinical characteristics and burden of illness in patients with hereditary angioedema: findings from a multinational patient survey.  Orphanet Journal of Rare diseases.  Muli-national: Australia, Austria, Canada, France, Germany, Spain, Switzerland and United Kingdom | To gain a comprehensive real-world understanding of the characteristics of hereditary angioedema (HAE) and its burden from the perspective of the patient. | N=242  G=67% female  A=18.92y, 43,8y mean  D=Hereditary angioedema  R=Different medical clinics | - Cross-sectional quantitative study (online web-based). - Investigating WP was one secondary outcome. | - Study specific questionnaire on clinical and sociodemographic aspects, including: The Angioedema Quality of Life questionnaire (AE-QoL), The 12-Item Short-Form Health Survey (SF-12v2), The Angioedema Control Test (AECT), The Hospital Anxiety and Depression Scale (HADS). - Questions on WP: The Work Productivity and Impairment questionnaire (WPAI). | - The mean (SD) percentage impairment measured by the WPAI was 24.59% for presenteeism, 24.18% for work productivity loss, and 33.88% for activity impairment. |
| **Rare respiratory diseases (ORPHA:97455)** | | | | | |
| **Cystic fibrosis (ORPHA:586)** | | | | | |
| 165  Albon et al 2021. Impact of Covid-19 on social determinants of health for adults with cystic fibrosis.  Journal of Therapeutic Advances in Respiratory Disease.  USA | To characterize the impact of COVID-19 related changes in social determinants at health in adult cystic fibroris (CF) population. | N=76  G=57.9% female  A=36.4y mean  D=Cystic fibrosis  R=CF clinic in South eastern USA | - Cross-sectional quantitative study. - Investigating WP was one secondary outcome. | - Study specific questionnaire included seven domains (housing, employment health insurance, transportation, utilities, food security and access to CF-medication). - Questions on WP: Not described. | - 52.6 % were working, 28.9% disability pension, 11.8 unemployed, 3.9% retired, 2.6% students. - Patients employed priori to COVID-19 were more likely to answer affirmatively to SDH screening in all domains, and were more likely to be related to loss of employment and concern for loss of employment - The domains most endorsed were employment and food security. |
| 109  Allgood et al 2018. Descriptions of the pain experience in adults and adolescents with cystic fibrosis.  Journal of Pain Management. Nursing.  USA | To explore and describe ways adolescents and adults with cytic fibrosis (CF) experience pain. | N=10  G=50% female  A=13-46y  D=Cystic fibrosis  R=Cystic Fibrosis Care Center | - Cross-sectional qualitative study. - Investigating WP was one secondary outcome. | - Individual interviews mainly focusing on pain experiences within five domains: Pain Characteristics, Activities, Relationships, and Health Care Team. - Questions on WP: Work/School Life were themes focused in the interviews. | - Employment rate were between: 45.9 to 62.2% in the age group early and middle adulthood, and in the age of 40 years 56.6% were retired. - Individuals with CF identify the disease as being painful; express how pain negatively affects all aspects of their lives, including restrictions on work-related aspects. |
| 88  Besier et al 2012.  Growing up with cystic fibrosis: achievement, life satisfaction, and mental health.  Journal of Quality of Life Research.  Germany | To analyze the vocational and social achievement, life satisfaction, and psychological well-being of adolescents and adults with cystic fibrosis (CF). | N=670  G=47,3% female  A=12-64y, 23.1y mean  D=Cystic fibrosis  R=Thirty-one German and one Austrian CF Centre | - Cross-sectional quantitative study (registry study). - Investigating WP was one secondary outcome. | - Study specific questionnaire of sociodemographic aspects, including: The Questions on Life Satisfaction, The Hospital Anxiety and Depression Scale (HADS). - Questions on WP. Not described. | - 45,9 % in the age group age: 21-30y were working, 62,2% in the age group 31-40y, and 43,4 % in the age group 41-61 years. |
| 166  Blau et al 2003.  Cystic fibrosis in adults: a changing scene.  Israel Medical Association Journal.  Israel | To describe experience with adult cystic fibrosis (CF), stressing the importance of adult-related health and psychosocial issues. | N=25  G=52 % female  A=20-50y  D=Cystic fibrosis  R=Kathy and Lee Graub CF Center, Schneider Children's Medical Center of Israel | - Cross-sectional quantitative study. - Investigating WP was one secondary outcome. | - Study specific questionnaire about sociodemographic factors, combined with data from patients' medical charts. - Questions on WP: Not described. | - 60% were employed, although often in part-time jobs to enable the time-consuming daily CF care. 28 % were students. |
| 68  Burker et al 2005.  Vocational attainment of adults with Cystic Fibrosis success in the face of adversity.  Journal of Rehabilitation.  USA | To describe the vocational status for adult with cystic fibrosis. | N=183  G=50 ,2% female  A=Not reported  D=Cystic fibrosis  R=Clinic, University of North Carolina | - Cross-sectional quantitative study. - Investigating WP was the primary aim/ outcome. | - Study specific questionnaire with a packed of questionnaires, of sociodemographic and clinical aspects. - Questions on WP: The standard vocation preparation (SVP). | - 48% were working, of these 84% were working outside home, 16% had been working, but had stopped working, 13,3 % students. The rest not described. - Skilled work increased work status. - 73% patients were working in skilled or semiskilled jobs. Patients with skilled work were more likely to maintain their position than those unskilled. |
| 69  Burker et al 2004. Psychological and educational factors. Better predictors of work status than FEV1, in adults with cystic fibrosis.  Paediatric Pulmonology.  USA | To examine if people who were working had higher forced experienced volume in 1 sec (FEV1), years of education, optimism and lower scores of depression and anxiety. | N=183  G=50,2% female  A=not reported  D=Cystic fibrosis.  R=Adult CF center | - Cross-sectional quantitative study. - Investigating WP was the primary aim/ outcome. | - Study specific questionnaire on sociodemographic and clinical aspects, including; The Spielberger State Trial Anxiety Inventory (STAI), The Beck Depression Inventory (BDI), The Life Orientation Test (LOT). - Questions on WP: Work status, how many hours a week, type of work, and disability. | - 48 % were working, of these 84 % were employed outside home, 16 % working inside home. - People working vs. not working had significantly lower depression scores, and significantly higher educational levels. Higher scores of BDI were associated with fewer number of work hours per week. - The results showing that physical function alone cannot predict vocational rehabilitation success. |
| 70Cicutto et al 2004. Factors affecting attainment of paid employment after lung transplantation.  Cicutto et al. 2004. Factors affecting attainment of paid employment after lung transplantation,  Journal of Heart Transplantion.  Canada | To identify the issues relating to paid employment after this procedure. | N=117  G=41 % female  A= 51y mean  D=Cystic fibrosis  R=Toronto General Hospital | - Prospective study. - Investigating WP was the primary aim/ outcome. | - Study specific questionnaire on sociodemographic and clinical factors. - Questions on WP: Questions about employment/work status before and after lung transplantation. | - 56 % were in paid work before lung transplanatation:, 37% After. - Younger age, higher educational level, better health and feeling physically able to work were significantly associated with being employed. - The respodents expressed desire to enter paid work. Medical and social factors influences attainment of paid work. |
| 71  Cumming al 2016. Predictors of employment participation following lung transplant.  Australian Occupational Therapy Journal.  Australia | To identify issues relating to occupational engagement in employment after surgery in cystic firbrosis (CF) patient. | N=100  G=55% female  A=50y mean  D=Cystic fibrosis with lung transplant.  R=one transplant service in Australia | - Prospective study. - Investigating WP was the primary aim/ outcome. | - Study specific questionnaire on sociodemographic factors, including: The Duke-UNC Functional Social Support Questionnaire, The Multidimensional Health Locus of Control, The Hospital Anxiety and Depression Scale (HADS) - Questions on WP: The Community Integration Questionnaire, including questions about productive activities. Questions about “have you worked after the lung transplation”, and if yes: “how long after lung transplantation”. | - Pre/post lung transplantation 52%/34% had paid work, 10 % / 8% unpaid work, 34%/ 49% not working, 2% / 2% students, 2% / 7% not stated. - No variables were significantly associated with work participation when added to multivariate model. - 42 % of those not retired from work prior to lung transplantation, were engaged in paid work after lung transplantation. |
| 72  Demars et al 2011. Employment experiences among adolescents and young adults with cystic fibrosis.  Journal of Disability & Rehabilitation.  USA | To examine the employment experiences in a population of adolescents and young adults with cystic fibrosis (CF) | N=68  G=57% female  A=16-25, 19,7y mean  D=Cystic fibrosis  R=the Children’s Hospital Boston CF Centre | - Cross-sectional quantitative study. - Investigating WP was `the primary aim/ outcome. | - Study specific questions on clinical and sociodemographic aspects obtained through review of medical record. - Questions on WP: Study specific questionnaire including 26 questions about work-related issues | - 37 % reported working 30 h or more per week, 46% working less than 20 h per week. - 47 % reported missing at least one day per month due to doctor`s visits, hospitalization, or other CF related care. 62%/59% reported that their employers/co-workers were informed about their disease. |
| 89  Duff et al 2014. Depression and anxiety in adolescents and adults with cystic fibrosis in the UK: A cross-sectional study.  Journal of Cystic Fibrosis.  United Kingdom | To establish the prevalence of anxiety and depression amongs people with cystic fibrosis (CF) compared to a normative sample; (ii) to establish the association between mood, demographic and clinical variables; and (iii) to provide guidance for specialist-referral decision-making. | N=2065 (1779 > 18 years)  G=51 % female  A=12-60y  D=Cystic fibrosis  R=Thirty-nine CF centers I UK | - Cross-sectional quantitative study. - Investigating WP was one secondary outcome. | - Study specific questionnaire on clinical and sociodemographic aspects including: The Hospital Anxiety and Depression Scale (HADS). The International Depression/ anxiety Epidemiological Study (TIDES) in the UK. - Questions on WP: Not described. | - 41,1% were working full time, 17,2% part time, 23%, not working due to health, 18.7% others. - Full-time employment associated with less anxiety. - The majority of adolescents and young adults with CF report disclosing their diagnosis at work. |
| 167  Dury et al 2021. Identifying specific needs in adult cystic fibrosis patients: a pilot study using a custom questionnaire.  Journal of BMC Pulmonary Medicine.  France | To identify the most frequent topics that Cystic fibrosis (CF) adults need to discuss with health care teams using a custom questionnaire including 62 items. | N=50  G=30 % female  A=27.6y mean  D=Cystic fibrosis  R=University Hospital of Reims. | - Cross-sectional quantitative study. - Investigating WP was one secondary outcome. | - Study specific questionnaire to identify CF patients’ needs, including 62 predefines topics divided in nine domains and some open-ended questions. - Questions on WP: Not described. | - 49% were working, 27% students, 32% not described. - Professional worries were not associated with employment status. - The most frequently selected topics of unmet needs were fatigue (20%), job or study (professional or scholar worries) (18%), |
| 86  Edwards et al 2010. Adults with cystic fibrosis and barriers to employment.  Journal of Disability & Society.  United Kingdom | To discusses findings from a study which explored the employment experiences of adults with cystic fibrosis (CF) from a social model perspective. | N=23  G=56 % female  A=20-43y  D= Cystic fibrosis  R=Advertisement in the magazine of Cystic Fibrosis Organization | - Mixed method cross-sectional study. - Investigating WP was the primary aim/ outcome. | - Combining study specific questionnaire on sociodemographic and clinical aspects, with qualitative interviews and follow-up telephone interview. - Questions on WP: Barrier securing and maintaining employment, unemployment and the participants own solutions. | - 65 % were working, 8% unemployed, 8% voluntary work, seeking for work.13% retired due to CF, 4% students. - Some participants experienced no realistic solution related to work, only retirement with obvious consequences for current income and pension later in life. |
| 168  Flewelling et al 2019.  Male gender and unemployment are associated with lower levels of perceived social support in adults with cystic fibrosis.  Journal of Psychosomatic Research.  USA | To examine factors associated with social support in adults with CF. | N=233  G=60,1 % female  A=33.6y mean  D=Cystic fibrosis  R=10 cystic fibrosis centers | - Cross-sectional quantitative study. - Investigating WP was one secondary outcome. | - Study specific questionnaire of sociodemographic aspects, including: The Interpersonal Support Evaluation List. - Questions on WP: Questions about working or not working. | - 59,5% were working.(rest not reported). - Being female and employed were associated with greater perception of social support. Being unemployed and male with having lower social support. |
| 73  Frangolias et al 2002.  Role of exercise and lung function in predicting work status in cystic fibrosis.  American Journal of Respiratory and Critical Care Medicine.  Canada | To determine the proportion of a cohort of adult patients with cystic fibrosis (CF) who are limited in their employment/education on the basis of current ATS impairment criteria;  Determine the proportion of a cohort of adult patients with CF who are disabled from employment or schooling. | N=73  G=36% female  A=29,6y mean  D=Cystic fibrosis  R=Cystic fibrosis clinic | - Cross-sectional quantitative study, including clinical examination. - Investigating WP was the primary aim/outcome. | - Study specific questionnaire of sociodemographic factors, including VO2 max-test. - Questions on WP: Specific questions related to work/school (type of job, missing workdays etc), full-time/part-time or unemployed. | - 56% were working/studying full time, 23% working/studying part time, 5.3 % unemployed. - Pulmonary function and exercise capacity measures have limited utility in predicting work status. - The data suggest that developing employment possibilities and increasing occasions for social support as the disease progresses improve psychosocial impact of living with CF. |
| 46  Habib et al 2015.  A Systematic review of factors associated with health-related quality of life in adolescents and adults with cystic fibrosis.  Journal of Annals of the American Thoracic Society.  Canada | To systematically review the literature to identify sociodemographic and clinical factors associated with HRQoL among adolescents and adults with cystic fibrosis (CF) | N=23 articles (2 articles dealt with work related aspects)  D=Cystic fibrosis | - Systematic review with quality assessment of the included articles. - Investigating WP was one secondary outcome. | - Synthesizing of the results from two articles about WP. - Questions on WP: Not described. | - One study found that employment was associated with higher scores on all The Cystic Fibrosis Quality of Life scale domains in bivariate analysis. - Another study found that employment was positively associated with Physical Functioning, Social Functioning, and Role Functioning. |
| 74  Haverman et al 2009.  Health related quality of life in cystic fibrosis: To work or not to work?  Journal of Cystic Fibrosis.  Belgium | To investigate whether patients with cystic fibrosis (CF) who are studying or working report a better HRQoL in comparison to nonworking/  studying patients. | N= 57 (37 adults)  G=49% female  A=26,7y mean  D=Cystic fibrosis  R=Outpatient clinic at the Adult Cystic fibrosis centre at the University Hospital Leuven | - Cross-sectional quantitative study (during outpatient clinic). - Investigating WP was the primary aim/outcome. | - Study specific questionnaire on sociodemographic aspects and medical/physical condition. - Questions on WP: The Cystic Fibrosis Quality of Life scale, teen/adult-version (CFQ-14+) (categorized yes/no working, full/part time). | - 65% were working/studying, 35% not working - Work was associated with better physical functioning, role perception and social functioning. Not-working was asscoated with greater disease severity and reported lower quality of life. - The decision to stop work/study for CF patients is difficult and affects patients' personal, social and financial well-being. |
| 75 Hogg et al.2007Hogg et al 2007.  Work disability in adults with cystic fibrosis and its relationship to quality of life.  Journal of Cystic Fibrosis.  Australia | To examine the percentage of participants with cystic fibrosis (CF) currently working and explore risk factors for work disability among adults with CF. | N=50  G=42% female  A=27 y mean  D=Cystic fibrosis  R=The Alfred Hospital, adult cystic fibrosis service. | - Cross-sectional quantitative study (during clinic). - Investigating WP was the primary aim/outcome. | - Study specific questionnaire of medical/ lung function and sociodemographic aspects, including: The Chronic respiratory Disease Index Questionnaire (CRDQ). - Questions on WP: a 23 item CF specific Questionnaire examining vocational issues and work disability. A disability index (DI) with 4 questions relating to ability to work. | - 40 % were working (rest not reported), - Work participation was associated to QoL index, age and hospital admission. - 68% reported that CF had significant impediments to employment. Few had sought vocational guidance (6%). Hours worked, perceived disability attitudes, age, time in hospital are more dependence on mastery of disease had greater impact than clinical indicators such as FEV severity scores. |
| 169  [Knudsen](javascript:;) et al 2016.  Associations between adherence, depressive symptoms and health-related quality of life in young adults with cystic fibrosis.  Journa of SpringerPlus  Denmark | - To examine the relationships among treatment adherence, symptoms of depression and health-related quality of life (HRQoL) in a population of young adults with cystic fibrosis (CF). | N=67  G=59 % female  A=18-30y  D=Cystic fibrosis  R=Specialized, outpatient clinic for cystic fibrosis at the Copenhagen University Hospital, Rigshospitalet | - Cross-sectional quantitative study. - Investigating WP was one secondary outcome. | - Study specific questionnaire on sociodemographic and clinical aspects, included: The Morisky Medication Adherence Scale (MMAS-8), The Major Depression Inventory (MDI) and The Cystic Fibosis Questionnaire-Revised-Teen/Adult version (CFQ-R). - Questions on WP: The CFQ-R included som questons about work. | - 84 % were employed/studying and 13 % incapable to work (due to health problems) - Work ability scores significantly lower on all domains of the CFQ-R, except Treatment Burden Work ability associated to health on adherence. |
| 76  Krivchenia et al 2016.  Long-term work participation among cystic fibrosis patients undergoing lung transplantation.  Journal of Cystic Fibrosis.  USA | To investigate socioeconomic and clinical factors predicting long-term employment outcomes in cystic fibrosis (CF) patients receiving lung transplantation. | N=745  G=not described  A=18-59y  D=Cystic fibrosis  R=United Network for Organ Sharing registry | - Prospective study. - Investigating WP was the primary aim/outcome. | - Study specific questionnaire of sociodemographic aspects and clinical aspects. - Questions of WP: Long-term employment status was determined by center-reported follow-up with data available until the 5th transplant anniversary, “working for income” or unemployed. | - 48% were working 5 years after lung transplantation - Lack of work experience and reliance on government health insurance at the time of transplant predict lower long-term work participation. Long-term employment outcomes were not negatively affected by comorbidities at or after transplantation in this cohort. |
| 77  Laborde-Casterot et al 2012.  Employment and work disability in adults with cystic fibrosis.  Journal of Cystic Fibrosis  France | To evaluate patients with cystic fibrosis (CF) education, occupation levels and risk factors for work disability. | N=207  G=48.3% female  A=30,7y mean  D=Cystic fibrosis  R=adult CF Centre in Paris, France | - Cross-sectional quantitative study. - Investigating WP was the primary aim/ outcome | - Study specific questionnaire on sociodemographic factors and management of treatments. - Questions on WP: Jobs were classified in occupational activity according to FAP-2003. Employment status were differentiated in four groups. | - 57% were working, 19% students, 6% were seeking employment and 18% were inactive. - High level of education associated with working. Low FEV1 and low educational level were the strongest predictive factors of disability. - CF patients had a higher educational level and were more likely to hold skilled jobs and to work part time than the general population. |
| 78  Leso et al 2021  Employment status and work ability in adults with cystic fibrosis.  Interational Journal of Environmentalt Research and Public Health.  Italy | To assess employment status, type of occupation performed, and work ability in a cohort of Italian cystic fibrosis (CF) patients, and to identify individual, pathological and occupational factors affecting labor force participation and capacity for work. | N=196  G=47% female  A=34,4y mean  D=Cystic fibrosis  R=Outpatients cystic fibrosis Reference Center of the University Hospital “Federico II” in Naples. | - Cross sectional quantitative study. - Investigating WP was the primary aim/ outcome. | - Study specific questionnaire of sociodemografic factors, and clinical testing pulmonary function (FVC, FV1), including: The Cystic Fibrosis Questionnaire-revised (CFQ-R). - Questions on WP: Work ability index (WAI). The International Standard Industrial Classification of All Economic Activities was used to classify productive field of employment. Jobs were divided into 10 occupational accitivies. | - 50 % were employed outside house, of these 66% working full time, 34% (n=33) part time - Non-working were significantly associated with younger age, disagnosed with CF earlier, lower FVC level, female, being single, lower educational level and lower Quality of Life. - 52% of the working CF population reported having a permanent contract, and most (82%) were employed in white-comllar professions. |
| 43  Leso et al 2021.  The impact of cystic fibrosis on the working life patients. A systematic review.  Journal of Cystic Fibrosis.  Italy | To review and address the impact of cystic fibrosis (CF) on the occupational functioning of patients. | N=15 articles were included (all dealt with work related aspects)  D=Cystic fibrosis | - Systematic review with qualitaty assessment of the included articles. - Investigating WP was the primary aim/ outcome. | - Systematic review with literature summary of employment status, job classifications, work disability, predicative factors for employment and disability. - Description on WP: Not described. | - Beeing employed ranged from 44% to 86% in the included studies. Of the working patients 37% to 68% reported having full-time jobs. - Age, personal characteristics, education, socio-economic status, seems to play a role in determining work disability. Disease severity parameters (e.g., lung function measurements, or personal, psycho-social, or economic conditions) were reported as determinant or co-determinant factors for the development of work-related disability. |
| 79  Lian, et al 2019.  Higher levels of education are associated with full-time work in adults ith cystic fibrosis.  Respiratory care.  Australia | To explore factors in adults with cystic fibrosis (CF) that predicted whether (i) someone was engaged in full-time paid work, and if (ii) those engaged in paid  work reported problems with absenteeism and/or presenteeism. | N= 50  G= 42% female.  A=25-36y, 30y mean  D=Cystic fibrosis  R=CF team at Sir Charles Gairdner Hospital. | - Cross-sectional quantitative study (as part of secondary analysis of data collected for RCT study). - Investigating WP was the primary aim/ outcome. | - Study specific questionnaire on sociodemographics, included: The Cystic Fibrosis Questionniare –Revised (QoL), The Treatment Adherence Questionnaire-Cystic Fibrosis. - Questions on WP: The Cystic Fibrosis Questionnaire Revised. The World Health Organization Health and Work Performance Questionnaire (HPQ). | - 68% were working full time, 18 % part time, 14 % unemployed. - Full-time work were associated with higher age and higher levels of education. - More participants reported having problems with relative absenteeism than presenteeism. |
| 110  McCarrier et al 2020.  The Cystic Fibrosis Impact Questionnaire: qualitative development and cognitive evaluation of a new patient-reported outcome instrument to assess the life impacts of cystic fibrosis.  Journal of Patient-Reported Outcomes  USA | To develop a new patient-reported outcome (PRO) measure to assess the impact of cysticf ibrosis on patients’ quality of life: the Cystic Fibrosis Impact Questionnaire (CF-IQ). | N=20 adults (22 children)  G=43% female  A=>18 y  D=Cystic fibrosis  R=Eight CF treatment centers across the USA | - Cross-sectional qualitative study for devolping an instrument. - Investigating WP was one secondary outcome. | - Individual qualitative interviews on how disease-related experiences, and functional changes in daily living following treatment. - Questions on WP: Not described. | - 60% were working full/part time, 15% were students, 25% not working (of these. 5 % retired, 10% unable to work, 5% homework, 5 % out of work for one year). - Many of the patients experienced high treatment burden and substantial time to manage the disease, with lconsequent imitations in work. |
| 80  Ochman et al 2019.  Employment after lung transplantation in Poland- a single centre study.  International Journal Occupation Medicine and Environment Health.  Poland | To assess the prospects of employment, along with the quality of life and physical activity of the lung transplant rcipients treated in the Silesian Center for Heart Diseases | N= 67(15 pre-transplanted)  G=38% female  A=23-72y, 43y mean  D=Cystic fibrosis and other lung diseases  R=Silesian Center for Heart Disease | - Prospective study. - Investigating WP was the primary aim/ outcome. | - Study specific questionnaire on sociodemographic and clinical aspects. - Questions on WP: Employment, income and how work affected the quality of life after lung transplanatation. | - 31 % were employed after lung transplantion (the rest, not reported). - Gender (more males were working), and higher education was significantly associated with increased WP. - 72.2 % among the employed stated that work improved their quality of life. |
| 124  Pakhale et al 2014.  Assessment of stigma in patients with cystic fibrosis. Journal of Pulmonary Medicine.  Canada | To explore if cystic fibrosis (CF) patients experience stigma, 2) to develop/adapt a scale to measure stigma in CF, and 3) to assess the adapted stigma scale’s psychometric properties in a prospective, cross-sectional cohort of adult CF patients. | N=45 quantitative study (5 in focus group)  G=42,2% female  A=23-56y, 30,3y mean  D=Cystic fibrosis  R=Multidisciplinary adult CF outpatient clinic in Ottawa | - Mixed-method cross sectional study. - Investigating WP was one secondary outcome. | - Combining qualitative focus group intervue with study specific questionnaire about socio-demographic aspects, including: The Short-form Health Survey for Medical Outcomes Study (SF-12), The SF-specific Quality of Life Questionnaire (CF-specific Qol), The Center of Epidemiological Studies Depression (CES-D), The Generalized Anxiety Disorder Questionnaire (GAD). - Questions on WP: Not described. | - 48.9% worked full time, 11.1% part time, 15% disability pension, 24.4% unemployed/retired. In the focus groups: 3 employed fulltime, one part time and one on disability pension. - Several experienced that stigma was distorting work. |
| 81  Radtke et al 2021. Predictors of long-term employment among patients with cystic fibrosis undergoing lung transplantation.  Journal of Swiss Medical Weekly.  Switzerland | To collect data of long-term employment in people with cystic fibrosis undergoing lung transplantation in Switzerland. | N=84  G=46.4% female A=29.9y mean  D=Cystic fibrosis R=Swiss university hospital, clinical data | - Cross-sectional quantitative study. - Investigating WP was the primary aim/ outcome. | - Study specific questionnaire on sociodemographic, anthropometric and clinical aspects. - Questions of WP: Example “Are you working or not”, and other aspects related to work. | - 41% were working and 59% not working. - Time of post-lung transplantation were the main factor associated with work ability. - Pre-transplant employment is the dominant factor influencing lung transplantation employment in people with cystic fibrosis. |
| 82  Saldana et al 2018.  More than a job: Career development of individuals with cystic fibrosis.  Journal of Work.  USA | To understand the employment experiences of people with cystic fibrosis (CF) and ultimately a substantive grounded theory of career development applicable to individuals with this disease. | N=10  G=60% female  A=18-27y  D= Cystic fibrosis  R= CF Foundation and the CF care center | - Cross-sectional qualitative study. - Investigating WP was the primary aim/outcome. | - Semi-structured individual qualitative interviews exploring their work experiences and career development process. - Questions on WP: Four questions related to work guided the inquiry (personal meaning of work, how CF influence employment and WP experiences). | - 70 % worked full time, 10% part time, and 20 % unemployed. - Four broad themes that influenced employment and career development were identified through analysis of the qualitative interviews: work is therapeutic, influence of CF on employment and career development, work environment, and, career development. |
| 113  Siklosi et al 2010.  Development, validation, and implementation of a questionnaire assessing disease knowledge and understanding in adult cystic fibrosis patients.  Journal of Cystic Fibrosis.  Ireland | To develop and validate a tool to investigate general knowledge, and use it to characterize how much adult CF patients know about their disease. | N=100  G=39% female  A=17-49y, 26y mean  D=Cystic fibrosis  R=St Vincent`s University Hospital | - Validation of a preliminary developed questionnaire. - Investigating WP was one secondary outcome. | - A preliminary questionnaire was developed including: 49 fixe-response questions, 2 open-ended questions and 9 questions. The demographic section consit of 14 items. - Questions of WP: Not described. | - 54% working (full- or part-time), 27% studying, 12% neither studyingor working. - No significant associations were found between CF knowledge score and demographic aspects, including work. |
| 83  Taylor-Robinson et al 2013.  A longitudinal study of the impact of social deprivation and disease severity on employment status in the UK cystic fibrosis population.  PLoS ONE, journal.  United Kingdom | Investigated, for the first time in a UK-wide cohort, longitudinal employment status, and its association with deprivation, disease severity, and time in hospital. | N=3458  G=44,2% female  A=20- 40y  D=Cystic fibrosis  R=The collection of data into the UK database. | - Prospective study - Investigating WP was the primary aim/ outcome. | - Study specific register data of annual review data registry between 1996-2010. - Questions on WP: The primary longitudinal outcome was any employment (defined as ‘‘full’’ or ‘‘part-time’), or unemployed. | - 53.4% were working, 46.6% not working. - Male sex, higher lung function and higer body mass index, andless time in hospital, were associated with improved employment chances. Greater deprivation, disease severity, and time in hospital associated with less employment chances. - Being out of work increases the risk of poverty and social exclusion, and is likely to further damage the health of the most disadvantaged people with cystic fibrosis. |
| 84  [Targett](javascript:;) et al 2014.  Employment in adults with cystic fibrosis.  Journal of Occupational Medicine.  United Kingdom | To explore the factors associated with employment status in adults with cystic fibrosis (CF). | N=254  G=46% female  A=21-34y, 26 y mean.  D=Cystic fibrosis  R=Cystic fibrosis centers in three Unite Kingdom university teaching hospitals | - Cross-sectional quantitative study design, including reviewing clinical records. - Investigating WP was the primary aim/ outcome. | - Study specific questionnaire on medical and sociodemographic. - Questions on WP: The Stanford Presenteeism Scale, The Cystic Fibrosis Questionnaire UK, The Work and Cystic Fibrosis Questionnaire (combining questions related employment and work adaptations with work disability index- derived from Hogg et al 2007). | - 65% were working/studying, median hours worked was 37.3 h/week. - Employment appeared to be most strongly associated with educational attainment, locality and HRQoL domains and not clinical parameters of severity. - 44% reported stopping a job due to CF; 47% felt CF had affected career choice and 23% reported workplace discrimination due to CF. |
| 90  Widerman et al 2000.  Health status and sociodemographic characteristics of adults receiving a cystic fibrosis diagnosis after age 18 years. Journal of Chest.  USA | To explore how age at diagnosis may be associated with selected disease and sociodemographic characteristics. | - N=7427 (786 diagnosed after 18y (AD), 6641 diagnosed before 18y (BD) - G=50 % female - A= >18y - D=Cystic fibrosis - R=registry data | - Cross sectional quantitative study (registry data). - Investigating WP was one secondary outcome. | - Study specific registry data on medical and sociodemographic aspects. - Questions on WP: Not described. | - 48.46 % working full time in AD groups versus 34.85 % in BD group. - WP associated with age of diagnosis, those receiving late diagnosis were more likely to be college graduates, married, and employed full time. |
| **Rare genetic vascular diseases (ORPHA:97962)** | | | | | |
| ***Marfan syndrome (ORPHA:558)*** | | | | | |
| 170  Bathen et al 2014. Fatigue in adults with Marfan syndrome, occurrence and associations to pain and other factors.  American Journal of Medical Genetic A.  Norway | To investigate how fatigue affect adults with Marfan syndrome in daily life, examining fatigue level and prevalence of severe fatigue compared to the general Norwegian population and individuals with other comparable conditions. | N=72  G=57% female  A=20-71y, 44,2y mean  D=Marfan syndrome  R=TRS National Resource centre for Rare diseases | - Cross-sectional quantitative study (postal). - Investigating WP was one secondary outcome. | - Study specific questionnaire about medical and sociodemographic aspects, including: The Fatigue Severity Scale (FSS), The Nordic pain Questionnaire. - Questions on WP: Not described. | - 57 % employed/students (the rest, not reported) - WP was significantly associated to fatigue. |
| 101  Benninghoven, et al 2017. Inpatient rehabilitation for adult patients with Marfan syndrome: an observational pilot study.  Orphanet Journal of Rare Diseases.  Germany | To confirm that our rehabilitation program was feasible and medically save. To apply standardized instruments to assess the impact of our rehabilitation program on physical fitness and psychological wellbeing of participants. | N=18  G=78% female  A=46.7y mean  D=Marfan syndrome (n=17), Loeys Dietz syndrome (n=1)  R=Recruited from patient organization and cardiology clinic | - Prospective study. - Investigating WP was one secondary outcome. | - Study specific questionnaire on sociodemographic and medical aspects, including: The Somatization Subscale Symptoms Checklist, The Fatigue Severity Scale, The Nottingham Health profile, The Short-form Health Survey for Medical Outcomes Study (SF-36), The Hospital Anxiety and Depression scale (HADS). - Questions on WP: Not described. | - 100 % (n=18) working, of these 88% (n=16) white-collar jobs, 11% (n=2) blue-collar jobs. |
| 59  De Bie et al 2004. Marfan syndrome in Europe. A questionnaire study on patients perceptions.  Journal of Community Genet.  Mulit-national study (7 European countries) | To assess the association between the severity of symptoms and subjectively experienced severity of regarding impact on relationships and pregnancies, psychosocial adjustment, and differences between the seven European countries. | N=857  G=55,3% female  A=>13y  D=Marfan syndrome  R=The patient support groups in Belgium, Denmark, France, Germany, the Netherlands, Switzerland and UK | - Cross-sectional quantitative study (postal) - Investigating WP was one secondary outcome. | - Study specific questionnaire on medical and sociodemographic aspects. - Questions on WP: Not described. | - 59,1% were working (full time/part time), 1,3% student, 30,9 % not working (of these 24,1 % not physically possible, 6,8% can not find job), 8,7% missing data. |
| 171  Fusar-Poli et al 2008. Determinants of quality of life in Marfan syndrome.  Journal of Psychosomatic.  Italia | To investigate quality of life, address the sociodemographic determinants influencing QoL, review available evidence and highlight the poor research in this area of people with Marfan syndrome. | N=36  G=75 % female  A=31.7y mean  D=Marfan syndrome  R=Outpatient clinic Marfan syndrome service | - Cross-sectional quantitative study. - Investigatin WP was one secondary outcome | - Study specific questionnaire on sociodemographic and medical aspects including: The Karnofsky Index, The Short-form Health Survey for Medical Outcomes Study (SF-36). - Questions on WP: Not described. | - 61% working, and 39 % not working. |
| 172  Goldfinger et al 2017.  Marfan syndrome and quality of life in the GenTAC Registry.  Journal of the American College of Cardiology.  USA | To study the association of QOL with self-reported demographics, health behaviors, physical impairment, surgeries, comorbid medical conditions, medications and Marfan syndrome (MFS) severity. | N=389  G=49 % female  A=18-7y, 41y mean.  D=Marfan syndrome  R=GenTAC Registry | - Cross-sectional quantitative study, part of a prospective cohort study - Investigating WP was one secondary outcome. | - Study specific questionnaire on medical and sociodemographic aspects, including: The Somatization Subscale of Symptoms, The Hospital Anxiety and Depression Scale (HADS), The Fatigue Severity Scale (FSS), The Short-form Health Survey for Medical Outcomes Study (SF-36). - Questions on WP: Not described. | - 48% working full-time, 6.2% part-time, 9.4% student, 5.7% self-employed, 7.3% retired, 15.6 % unable to work, 4.5% unemployed, 3.2% homemakers. - Working was positively associated to QoL. Highest MFS severity score was associated with work disability. |
| 173  Moon et al 2016.  Structural equation modeling of the quality of life for patient with syndrome.  Journal of Health and Quality of life outcome.  Korea | To build QOL structural model of patient with MFS, verify it goodness of fit and determine the factors that affect the QoL | N=218  G=not reported  A=>20y  D=Marfan syndrome  R=Cardiology Outpatient department, Soul. | - Cross-sectional quantitative study (in clinic). - Investigating WP was one secondary outcome. | - Study specific questionnaire on clinical and sociodemographic aspects, including: The Hospital Anxiety and Depression Scale (HADS), The Fatigue Severity Scale (FSS), The Body Image State Scale (BISS), The Vas-Scale measuring pain, The Short-form Health Survey for Medical Outcomes Study (SF-36). - Questions on WP: Not described. | - 69.2 % were working (the rest, not reported). |
| 45  Nielsen et al 2019. A review of psychosocial factors of Marfan syndrome: Adolescents, adults, families and providers.  Journal of Pediatic Genetics.  USA | To review the current literature of Marfan syndrome (MFS) and its impact on adolescents, adults and important consideration for providers. | N=41 paper (8 addressed work-related aspects)  D=Marfan syndrome | - Literature review (no quality assessment of included articles)- - Investigating WP was one secondary outcome. | - Including 8 articles dealing with different aspects of work. - Questions on WP: Nor described. | - Work and possibility to build carrier influence QoL. WP was associated with higher self-esteem, higher life satisfaction and QoL |
| 174  Peters et al 2005.  Living with Marfan syndrome IV, Coping with stigma.  Journal of Clinical Genetics.  USA | To offer data on  perceptions of stigma from a cross-sectional study. | N=174  G=58% female  A=>18y  D=Marfan syndrome  R=National  Marfan Foundation | - Cross-sectional quantitative study (postal). - Investigating WP was one secondary outcome. | - Study specific questionnaire on sociodemographic and medical history, including: The Perceived Stigma Questionnaire, The Illness Perception Questionnaire, The Illness Perception Questionnaire (IPQ), The Center for Epidemiological Studies Depression Scale (CES-D), The Rosenberg Self-Esteem Scale. - Questions on WP: Not described | - 64% were working full time or part-time, (55,7% full time and 8,3% part-time work) ( the rest, not reported) - 47% reported that diagnosis affected choice of occupation or employment, 24% idiagnosis nfluenced decision to remain in dissatisfying job, and 20% experienced workplace discrimination. |
| 175  Polos et al 2020. Psychological factors affecting Marfan syndrome patients with or without cardiac surgery.  Journal of Annals Palliative Medicine.  Hungary | To assess the psychological and psychosocial aspects of Marfan syndrome (MFS) with the goal of identifying a means of improving disease management for patients. | N=66  G=52% female  A=34y mean  D=Marfan syndrome (operated and non-operated)  R=The Heart and Vascular Center at Semmelweis University in Budapest | - Cross-sectional quantitative study (in clinic). - Investigating WP was one secondary outcome. | - Study specific questionnaire on sociodemographic and clinical aspects: The Mini-Mental State Examination (MMSE), The State Trait Anxiety Inventory (STAI), The Beck Depression Inventory (BDI), The Somatic Symptom Severity Scale, Patient Health Questionnaire, PHQ15, The Devins Illness Intrusiveness Rating Scale. - Questions on WP: Not described. | - 38 1% were working, 2,4 % unemployed, 14, 3 % retired, 38.1% students, 7.1 % others. |
| 176  Rao et al. 2016. Quantifying health status and function in Marfan syndrome.  Journal of Surgical Orthopaedic Advances.  USA | To understand the self-perception of physical and mental well-being in patients with MFS. | N=230,  G=58 % female  A=14-82y, 44y mean  D=Marfan syndrome  R=The Marfan Patient Association | - Cross-sectional quantitative study. - Investigating WP was one secondary outcome. | - Study specific questionnaire on medical and sociodemographic aspect including: The Short-form Health Survey for Medical Outcomes Study (SF-36). - Questions on WP: Not described. | - 31% were working (of these 89 % had reduced weekly work hours, 45 % due to MFS), 26 % had retired. Mean age for retiring was 48,5y. - Early retiring associated to fatigue, pain and extensive medical treatment. - 82% patients lost on average 6.5 month from work because of MFS treatment. |
| 177  Speed, et al 2016. Characterization of pain, disability, and psychological burden in Marfan syndrome. American Journal of Medical Genetics.  USA | To characterize pain in a cohort of adults with Marfan syndrome and investigate demographic, physical and psychological factors associated with pain. | N=245  G=73% female  A=18-75y  D=Marfan syndrome  R=The Marfan foundation web-site | - Cross-sectional quantitative study (web-based). - Investigating WP was one secondary outcome. | - Study specific questionnaire of medical and sociodemographic aspects, including: The Pain catastrophizing scale (PCS), The Oswestry disability index (ODI), The Beck depression inventory-II (BDI), The Insomnia severity index (ISI), The Short-form Health Survey for Medical Outcomes Study (SF-36). - Questions on WP: Not described. | - 56% were employed, 44% not employed. - The unemployed had significantly greater severity across all psychological factors. Pain-related disability was associated with unemployment. |
| 85  Velvin et al 2015.  Work participation in adults with Marfan syndrome: Demographic characteristics, MFS related health symptoms, chronic pain, and fatigue.  American Journal of clinical Genetics.  Norway | To investigate WP in adults with Marfan syndrome and how health related consequencesand other factors might influence work participation, compare health problems in young adults with MFS to older, related to work. | N=70  G=57% female  A=20-67y, 43y mean  D=Marfan syndrome  R=TRS National Resource Centre for Rare diseases | - Cross-sectional quantitative study (postal). - Investigating WP was the primary aim/outcome. | - Study specific questionnaire about medical and sociodemographic aspects including: The Fatigue Severity Scale (FSS), The Nordic pain Questionnaire. - Questions on WP: Items of questions from the National Labor Force Survey, questions about present employment status, occupation, degree of disability pension and age of withdrawal from work. | - 53% were working (full time 41%,/ part time 12%), 6% students, 41 % disability pension. - Work participation were significantly associated to educational level, age and fatigue. - The average age for leaving work was low. Few had recievedany type of work adaptations prior to retiring from work. |
| 178  Velvin et al 2016.  Satisfaction with life in adults with Marfan syndrome: associations with health-related consequences of MFS, pain, fatigue, and demographic factors.  Journal of Quality of Life Research.  Norway | To explore satisfaction with life (SWL) among adults with MFS and the association between SWL and medical and demographic factors, contact with social and health service. | N=73  G=57% female  A=20-71y, 44, 2y mean  D=Marfan syndrome  R=TRS National Resource centre for Rare diseases | - Cross-sectional quantitative study (postal). - Investigating WP was one secondary outcome. | - A study specific questionnaire about medical and sociodemographic aspects including: The Fatigue Severity Scale (FSS), Nordic pain Questionnaire, Satisfaction with Life Scale. - Questions on WP: Items of questions from Norwegian National Labor Force Survey. | - 52% were working fulltime/part time, 6% students. 42% (disability pension/retired). - WP associated with higher Satisfaction with life. |
| 44  Velvin et al 2015. Systematic review of the psychosocial aspects of living with Marfan syndrome.  Journal of Clinical Genetics.  Norway | To explore the literature on psychosocial aspects of Marfan syndrome. | N=20 articles (5 papers addressed work-related aspects) | - Systematic review, with quality assessment of the included articles. - Investigating WP was one secondary outcome. | - Including 5 articles dealing with different aspects of work. - Questions on WP: Not described. | - Four studies showed that approximately 60 % of adults with MFS were working (full/ or part time) or studying. |
| ***Loeys-Dietz syndrome (ORPHA:60030) and vascular Ehlers-Danlos syndrome (ORPHA:286)*** | | | | | |
| 179  Johansen et al 2019. Adults with Loeys–Dietz syndrome and vascular Ehlers–Danlos syndrome: A cross-sectional study of health burden  Perspectives.  American journal of Medical Genetics A.  Norway | To present self-reports of socio-demographic characteristics, medical aspects and health services utilization of adults with verified vEDS and LDS, | N=52  G=58 % female  A=>18y, 43,3y mean  D=vEDS (n=18) and LDS (n=34)  R=Clinic register (TRS National Resource Center for rare diseases) | - Cross-sectional quantitative study (postal). - Investigating WP was one secondary outcome. | - Study specific questionnaire of medical and sociodemographic aspects, including: The Fatigue Severity Scale (FSS), The Hospital Anxiety and Depression scale (HADS). - Questions on WP: Items of questions from Norwegian National Labor Force Survey. | - 39% were working full or part time (vEDS39%, LDS 38%), 62% had disability pension/retirement (vEDS 61%, LDS 62%). - Higher age was associated with receiving disability pension. |
| 180  Johansen et al 2020.  Adults with Loeys-Dietz syndrome and vascular  Ehlers-Danlos syndrome: a cross-sectional study of  patient experiences with physical activity.  Journal of Disability & Rehabilitation.  Norway | To study patient experiences with physical activity among persons with Loeys-Dietz- or vascular Ehlers-Danlos syndrome. | N=52 (vEDS=18, LDS=34  G=58% female  A=18-68y  D=vEDS and LDS  R=TRS National  Resource Centre for Rare Disorders | - Cross-sectional quantitative study (postal). - Investigating WP was one secondary outcome. | - Study-specific questionnaire of medical and demographic aspects including: The Fatigue Severity Scale (FSS), The Hospital Anxiety and Depression scale (HADS). - Questions on WP: Items of questions from Norwegian National Labor force Survey. | - 27% full time employed (LDS=29%, vEDS=22%) (the rest, not reported). - Physical activity level not associated to work participation or level of disability. |
| ***Rare disease with thoracic aortic aneurysm and aortic dissection (HTAAD) (Orpha:281514)*** | | | | | |
| 181  Thijssen et al 2020.  Male–female differences in quality of life and coping style in patients with Marfan syndrome and hereditary thoracic aortic diseases.  Journal of Genetic Counselors.  Netherlands | To evaluate HRQOL in hereditary thoracic aortic diseases patients compared to the general population; assess male–female differences in HRQOL and factors associated with HRQOL; evaluate coping styles in male and female HTAD patients and identify factors associated with acceptance. | N=142 (107 working age)  G=46% female  A=42,1y mean  D=MFS (86,5%), LDS (5,6%), ACTA 2 (0,7%), others (3,5%), none (3,5%)  R= HTAD outpatient clinic | - Cross-sectional quantitative study (in clinic). - Investigating WP was one secondary outcome. | - Study specific questionnaire on sociodemographic, including: The Hospital Anxiety and Depression Scale (HADS), The Nijmegen Clinical Screening Instrument (NCSI), The Rotterdam Disease Specific Questionnaire (RDSQ), The Short-form Health Survey for Medical Outcomes Study (SF-36). - Questions on WP: Not described. | - 66% working (the rest, not reported). - Employment was strongly associated with better scores on the NCSI. Employment was associated with better quality of life. |
| 123  Thijssen et al 2020.  Health-related quality of life and lived experiences in males and females with thoracic aortic disease and their partners.  Journal of Open Heart.  Netherlands | To describe HRQOL in patients with thoracic aortic disease (TAD), cardiovascular screening participants and their partners; identified factors associated with HRQOL; and explored lived experiences and feelings of anxiety or depression using a mixed methods design. | N=261 /11 in in-depth interview  G=36,7% female  A=52,9y mean  D=Marfan syndrome (3,8%), Loeys-Dietz syndrome (3,8%), EDS (0.8%), others (4.6%), confirmed genetic mutations (23,4%)  R=HTAD outpatient clinic | - Mixed method cross sectional study ( in clinic) - Investigating WP was one secondary outcome. | - Combining quantitative study specific questionnaire on demographic and clinical aspects, included: The Short-form Health Survey for Medical Outcomes Study (SF-36), The Hospital Anxiety and Depression Scale (HADS), The Rotterdam Disease Specific Questionnaire (RDSQ), with in-depth interviews of a selected group. - Questions on WP: Not described. | - 36,8% paid work, 2,7% volunteer job, 21,8% retired, 6,1% students, 6,1% unable to work, 4,5% unemployed. |
| 111  Velvin et al 2019. Physical exercise for people with hereditable  thoracic aortic disease. A study of patient  perspectives.  Journal of Disability and Rehabilitation.  Norway | To explore aspects  related to physical exercise as highlighted by the patients themselves. | N= 36  G=56% female  A=22-71y, 48y mean  D=Marfan syndrome (n=14), Loeys Dietz syndrome (n=11), vascular Ehlers Danlos syndrome (n=11)  R=TRS National  Resource Centre for Rare Disorders | - Cross sectional qualitative study. - Investigating WP was one secondary outcome. | - Focus group interviews with semi-structured interview guide, included a short studyspecific questionnaire of sociodemographic aspects. - Questions on WP: Not described. | - 39% were working full time, 13% part time, 39% disability pension/retirement, 9% student. - Many experienced that fatigue and pain was difficult to manage in work life. |
